# Genome-Wide Characterization, Identification and Expression Profile of MYB Transcription Factor Gene Family during Abiotic and Biotic Stresses in Mango (*Mangifera indica*)

**DOI:** 10.3390/plants11223141

**Published:** 2022-11-16

**Authors:** He Zhang, Zhixin Liu, Ruixiong Luo, Yu Sun, Cuifeng Yang, Xi Li, Aiping Gao, Jinji Pu

**Affiliations:** 1 Key Laboratory of Integrated Pest Management on Tropical Grops, Ministry of Agriculture and Rural Affairs, Environment and Plant Protection Institute, Chinese Academy of Tropical Agricultural Sciences, Haikou 571101, China; 2 College of Agricultural, Guizhou University, Guiyang 550225, China; 3 Guangxi Key Laboratory of Biology for Mango, College of Agriculture and Food Engineering, Baise University, Baise 533000, China; 4 Tropical Crops Genetic Resources Institute, Chinese Academy of Tropical Agricultural Sciences, Haikou 571101, China; 5 Institute of Tropical Bioscience and Biotechnology, Chinese Academy of Tropical Agricultural Sciences, Haikou 571101, China

**Keywords:** mango, MYB transcription factor gene family, bioinformatics, phylogenetic tree, expression profile, Ka/Ks

## Abstract

Mango (*Mangifera indica*) is an economically important fruit tree, and is cultivated in tropical, subtropical, and dry-hot valley areas around the world. Mango fruits have high nutritional value, and are mainly consumed fresh and used for commercial purposes. Mango is affected by various environmental factors during its growth and development. The *MYB* transcription factors participates in various physiological activities of plants, such as phytohormone signal transduction and disease resistance. In this study, 54 *MiMYB* transcription factors were identified in the mango genome (371.6 Mb). A phylogenetic tree was drawn based on the amino acid sequences of 222 MYB proteins of mango and Arabidopsis. The phylogenetic tree showed that the members of the mango MYB gene family were divided into 7 group, including Groups 1, -3, -4, -5, -6, -8, and -9. Ka/Ks ratios generally indicated that the *MiMYBs* of mango were affected by negative or positive selection. Quantitative real-time PCR showed that the transcription levels of *MiMYBs* were different under abiotic and biotic stresses, including salicylic acid, methyl jasmonate, and H_2_O_2_ treatments, and *Colletotrichum gloeosporioides* and *Xanthomonas campestris* pv. *mangiferaeindicae* infection, respectively. The transcript levels of *MiMYB5*, *-35*, *-36*, and *-54* simultaneously responded positively to early treatments with salicylic acid, methyl jasmonate, and H_2_O_2._ The transcript level of *MiMYB54* was activated by pathogenic fungal and bacterial infection. These results are beneficial for future interested researchers aiming to understand the biological functions and molecular mechanisms of *MiMYB* genes.

## 1. Introduction

Mango (*Mangifera indica* L., 2*n* = 40) is an economically important evergreen fruit tree in the Anacardiaceae family [1,2]. In 2020, the global production of mangoes, mangosteens, and guavas yielded 5.71 million hectares, 57.3 million tons (http://www.fao.org/faostat/, accessed on 1 October 2022). In China, the cultivation of fresh mango has been practiced on a considerable scale for many years. Mango is affected by various environmental factors during its growth and development. Plant transcription factors contain special structures that can combines cis-acting elements of stress-related genes to regulate plant responses to abiotic and biotic stresses [3]. Plant growth and development are controlled by multigene families and superfamilies. The MYB transcription factor family exists in a variety of plants and is also one of the larger transcription factor families in all plants [4,5]. The first MYB gene identified in plants was the *Zea mays* C1 gene [6]. MYB transcription factors participate in various physiological activities of plants, such as phytohormone signal transduction, disease resistance, and responses to biotic and abiotic stresses [5,7,8,9]. MYB proteins contain a highly conserved MYB DNA-binding domain at their *N*-terminus, which is approximately 50–53 amino acid residues in length, and typically contains one to four imperfect MYB repeats (SONT domains), named R1, R2, R3, and R4 [10].

Bioinformatics and publicly released whole genome have widely analyzed the number, classification, genetic structure, and evolutionary and expression modes of *MYB* genes, especially in plants [11]. To date, the plant transcription factor database (PlantTFDB, http://planttfdb.gao-lab.org/index.php, accessed on 7 March 2021) contains approximately 22,032 MYB, and 15,369 MYB-related, sequences in 165 species, with 3–489 MYB members. These include Arabidopsis (*Arabidopsis thaliana*) and rice (*Oryza sativa*) [12], cotton (*Gossypium hirsutum*) [10], soybean (*Glycine max*) [13], apple (*Malus pumila*) [14], cassava (*Manihot esculenta*) [15], wheat (*Triticum aestivum*) [16], *Populus trichocarpa* [17], and *Nicotiana tabacum* [18]. However, due to the lack of a genome sequence, the MYB transcription factor of mango has not been identified. Recently, Wang et al. [2] and Li et al. [19], respectively, completed the genome-wide sequencing of mango and obtained genome-wide information with a size of 371.6 Mb and 20 chromosomes. The release of the mango chromosome-scale reference genome provides a basis for future research.

To identify the *MiMYBs* in mango, using PlantTFDB to search for proteins with the characteristic domains of the plant MYB transcription factors [20]. 54 *MiMYBs* were identified and subsequently subjected to systematic analyses, including phylogenetic tree analysis, conserved motifs identification, and expression profiles in response to abiotic and biotic stress treatments. The results of our study provide information that could be fundamental to determining the molecular and regulatory mechanisms of *MiMYB* transcription factors in mango in disease resistance and stress response.

## 2. Results

### 2.1. Identification and Bioinformatics Analysis of MYB Transcription Factor Genes in Mango

In this study, 54 *MiMYBs* were identified in mango and renamed *MiMYB1* to *MiMYB54* (Table S1, Text S1). Information on these *MiMYBs* and their corresponding proteins is shown in Table S1 and Text S2. The sizes of the deduced MiMYB proteins varied markedly from 197 amino acids (*MiMYB32*) to 1121 amino acids (*MiMYB13*). The corresponding molecular masses varied from 22.64 kDa (*MiMYB32*) to 125.84 kDa (*MiMYB13*) and the predicted isoelectric points (pI) varied from 4.87 (*MiMYB43*) to 9.67 (*MiMYB25*). The number of exons in *MiMYBs* varies from 2 to 12. Two exons interrupted by one intron and three exons interrupted by two introns were the most common structures, accounting for 44.44% and 33.33% of the total *MiMYBs*, respectively. *MiMYB26* and *MiMYB45* contain 12 exons and 11 introns (Table S1). These results show that the structures of *MiMYBs* are diverse.

### 2.2. Phylogenetic, Ka/Ks Analysis, and Synteny Analysis of MYB Transcription Factor Genes in Mango

**Figure 1:**
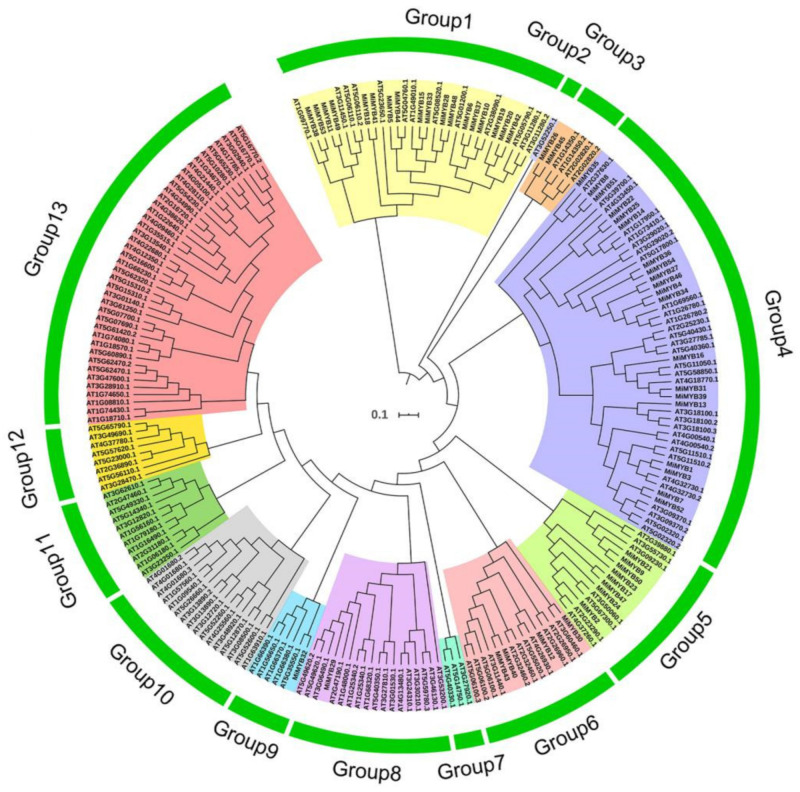
Phylogenetic analysis of MYB proteins from mango and Arabidopsis. The neighbor-joining (NJ) phylogenetic tree was constructed with MYB domains of MYBs from mango (54 members) and Arabidopsis (168 members) using ClustalX and MEGA 7.0 software with 1000 bootstrap. The MYB proteins were grouped into 13 groups (1–13). ATs are the MYB proteins from Arabidopsis. MiMYBs are the MYB proteins from mango.

To study the evolutionary relationships between mango MYB proteins and MYBs from Arabidopsis, a neighbor-joining (NJ) phylogenetic tree was created based on multiple alignments of the predicted amino acid sequences of the MYB domains from mango and Arabidopsis using ClustalX and MEGA 7.0 software (Figure 1). As shown in Figure 1, MYB proteins were classified into 13 groups with a branch length of 0.917 using MEGA 7.0. MiMYBs were distributed in Group 1, -3, -4, -5, -6, -8 and -9, respectively. Among the groups, Group 4 (20 members) and Group 1 (18 members) were the two largest groups and these two groups both represented more than 33% of the total *MiMYB* members. In contrast, six groups contained no *MiMYB* genes.

Because the ratio of Ka/Ks (synonymous/non-synonymous) is an important indicator of selection pressure occurring at the protein level, we evaluated the values of Ks and Ka as well as the ratio of Ka/Ks (Table S2). A total of 150 segmental duplicated gene pairs displayed Ka/Ks < 1, ranging from 0.32 to 1, with the mean values of Ka, Ks, and Ka/Ks being 2.26, 2.08, and 1.33, respectively. A total of 380 segmental duplicated gene pairs displayed Ka/Ks > 1, ranging from 1 to 2.08, with the mean values of Ka, Ks, and Ka/Ks being 2.70, 2.61, and 0.86, respectively. The Ka/Ks ratios generally indicated that the *MiMYBs* of mango were affected by negative or positive selection. To deduce the evolutionary relationship of MiMYB genes, syntenic analysis was performed for MiMYB genes using TBtools (Figure S1). The results showed that there are many synteny blocks between MiMYB genes in the mango genome. These data indicated that the MiMYB genes might have evolved from loss or duplication.

### 2.3. The Conserved Domains and Motifs Analysis of MYB Transcription Factors in Mango

**Figure 2:**
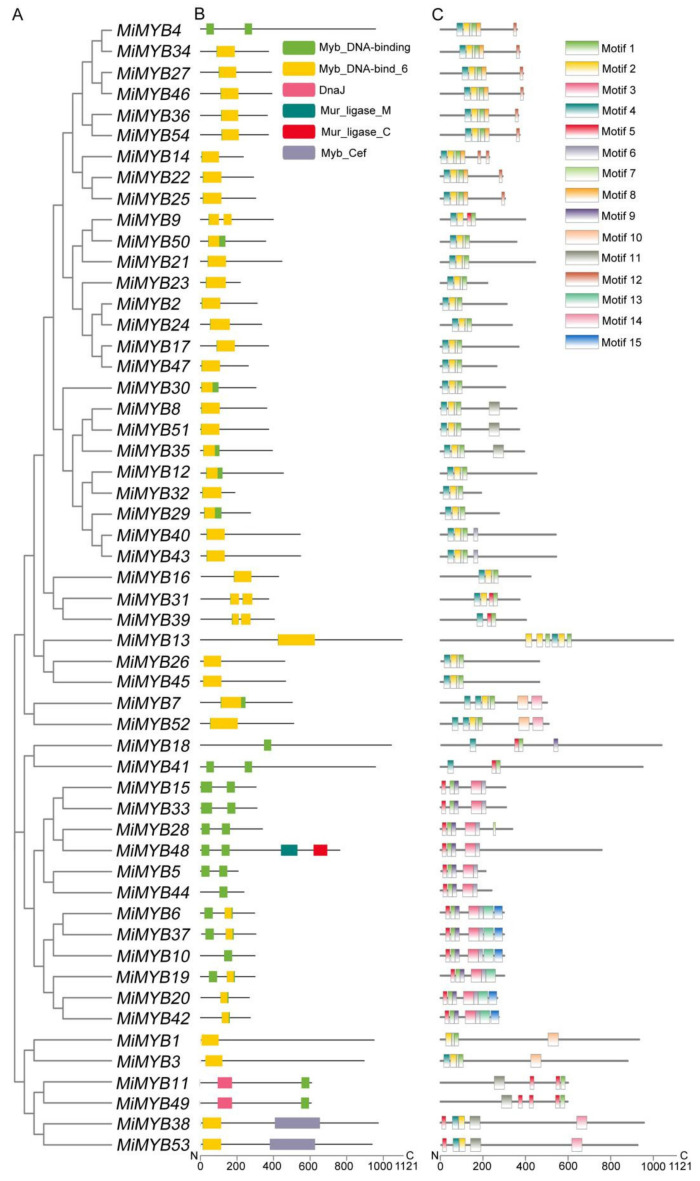
Conserved domains and motif compositions of MYB proteins in mango. (**A**) Phylogenetic tree of *MiMYB* transcription factors. (**B**) Conserved domains of *MiMYB* transcription factors. Differently colored boxes represent different domains. (**C**) Conserved motifs for MiMYB proteins in mango. Different motifs were shown with different gradient-colored boxes and numbers (1–15). The gray lines represent the non-conserved sequences. The lengths of domains and motifs can be estimated using the scale at the bottom.

To obtain detailed information about the structures of these MYB proteins in mango, the deduced amino acids collected from the NCBI were aligned, and the conserved domains were analyzed using Pfam and visualized using TBtools software. As shown in Figure 2, the MiMYB proteins contain Myb DNA binding, Myb DNA bind 6, DnaJ, Mur ligase M, Mur ligase C, and Myb Cef domains. Some of the *MiMYB* members had a single Myb DNA binding domain, such as *MiMYB10*, *-18*, or *-44*. However, *MiMYB4*, *-5*, *-15*, *-28*, *-33*, and *-41* proteins contained two separate Myb DNA binding domains, which are located in the *N*-terminus and the middle of the protein. Analyses revealed that the MYB domains of mango were divergently distributed across the gene not only in the *N*-terminal, but also in the middle or C-terminal regions of the proteins, demonstrating the high diversity of amino acids.

To further detect the structural features of mango MYBs, conserved motifs were analyzed according to their phylogenetic relationships. A total of 15 conserved motifs in mango MYBs were found with lengths of 11 to 50 amino acids, using MEME software and further visualization by TBtools. As shown in Figure 2, Motif 2 was a relatively conserved motif, being present in most of the family members which made up the core sequence of the single MYB domain. Motif 3 was a distinct motif with a conserved ‘SHAQKY’ sequence. These results indicate that although *MiMYB* transcription factors belonging to the same group are different, they also have shared identical or similar motif compositions.

### 2.4. Differential Expression of MiMYBs in Response to SA, MeJA, and H_2_O_2_ Treatments

**Figure 3:**
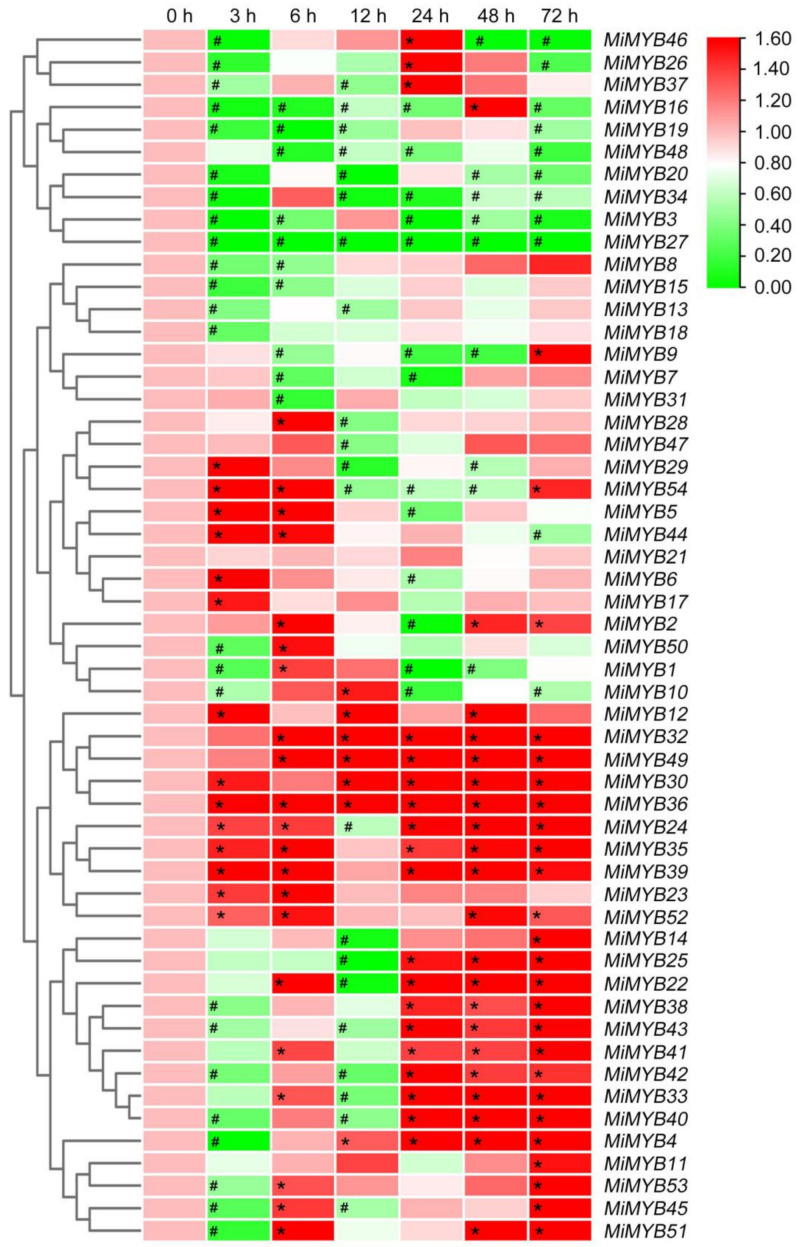
Differential expression of *MiMYBs* in response to SA treatments. Relative transcript levels of *MiMYBs* in 5 mmol/L SA treatment at 0, 3, 6, 12, 24, 48, and 72 h. The relative transcript levels of *MiMYBs* in the no-treatment mango leaves (0 h) were normalized to 1.0. The color scale represents gene expression levels with high transcript levels (red) or low transcript levels (green). The calculation method was 2^−∆∆CT^, and ‘*’ means the value exceeds 1.5, while ‘#’ means the value is below 0.5.

**Figure 4:**
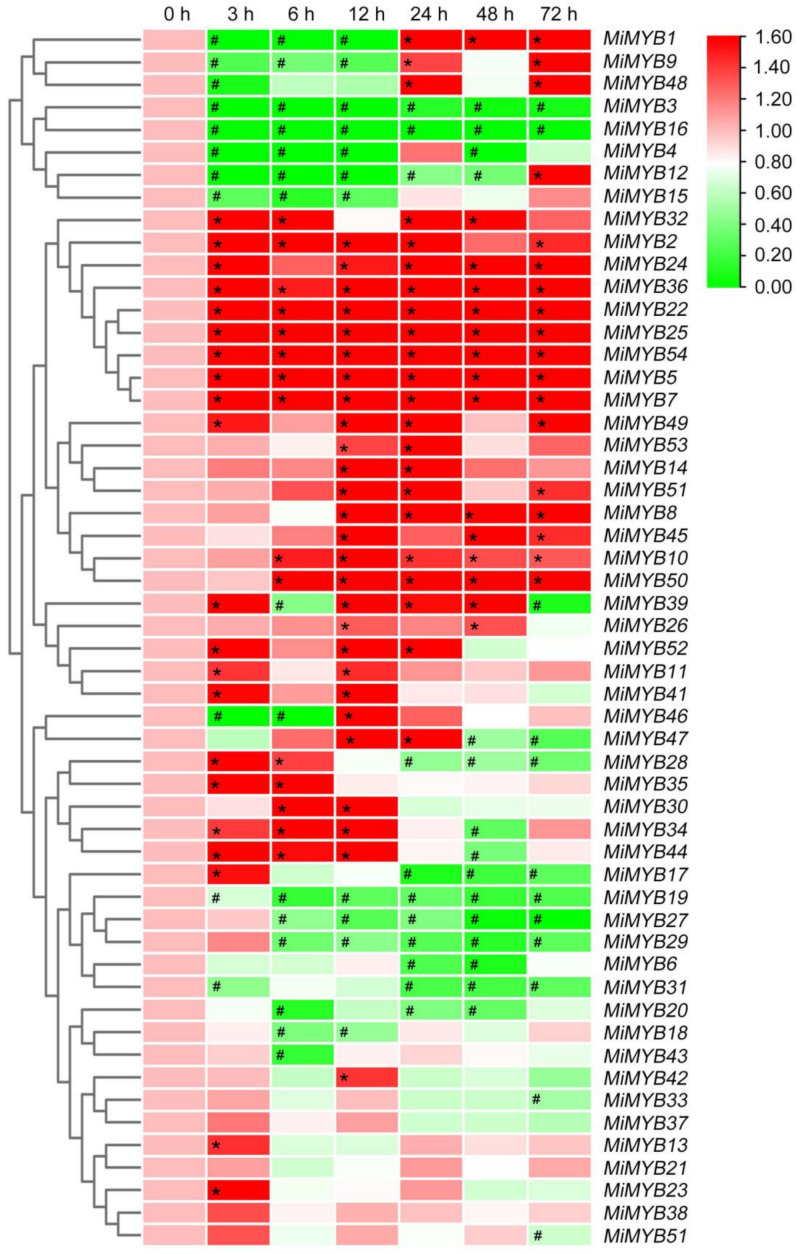
Differential expression of *MiMYBs* in response to MeJA treatments. Relative transcript levels of *MiMYBs* in 5 mmol/L MeJA treatment at 0, 3, 6, 12, 24, 48, and 72 h. The relative transcript levels of *MiMYBs* in the no-treatment mango leaves (0 h) were normalized to 1.0. The color scale represents gene expression levels with high transcript levels (red) or low transcript levels (green). The calculation method was 2^−∆∆CT^, and ‘*’ means the value exceeds 1.5, while ‘#’ means the value is below 0.5.

**Figure 5:**
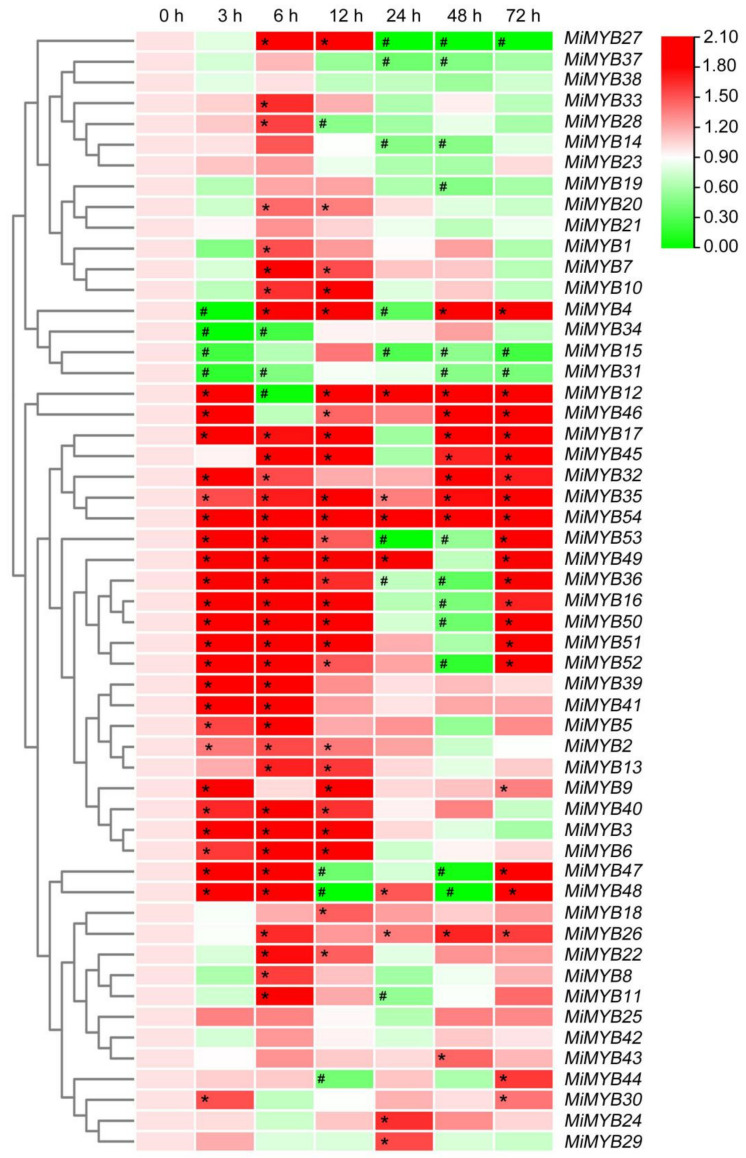
Differential expression of *MiMYBs* in response to H_2_O_2_ treatments. Relative transcript levels of *MiMYBs* in 10 mmol/L H_2_O_2_ treatment at 0, 3, 6, 12, 24, 48, and 72 h. The relative transcript levels of *MiMYBs* in the no-treatment mango leaves (0 h) were normalized to 1.0. The color scale represents gene expression levels with high transcript levels (red) or low transcript levels (green). The calculation method was 2^−∆∆CT^, and ‘*’ means the value exceeds 1.5, while ‘#’ means the value is below 0.5.

**Figure 6:**
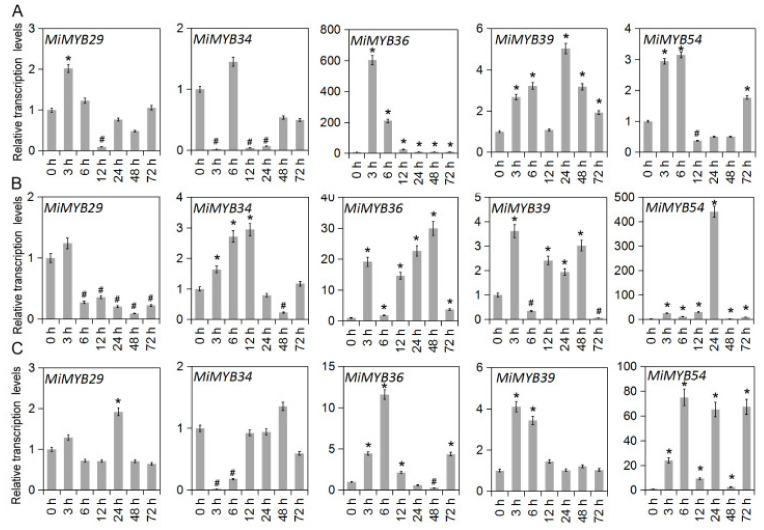
Differential expression of *MiMYB29*, *-34*, *-36*, *-39*, *-54* in response to SA, MeJA, and H_2_O_2_ treatments. (**A**) shows the results of the SA treatment, (**B**) shows the results of the MeJA treatment, and (**C**) shows the results of the H_2_O_2_ treatment. The calculation method was 2^−∆∆CT^, and ‘*’ means the value exceeds 1.5, while ‘#’ means the value is below 0.5.

The qRT-PCR primers of the *MiMYB* genes were designed via Primer 5.0 software and Primer3Plus to determine the optimal primers (Table S3, Text S1). Primer specificity was ensured through PCR and DNA agarose gel electrophoresis (Figure S2). To further investigate the potential functions of *MiMYBs* under abiotic stress conditions, cDNA samples were obtained using mango seedlings exposed to either SA, MeJA, or H_2_O_2_ stress for 0, 3, 6, 12, 24, 48, and 72 h. As shown in Figure 3 and Table S4, *MiMYB32* and *MiMYB36* showed an overall up-regulated trend at 7 time periods under SA treatment; however, *MiMYB21* was not activated during this process. *MiMYB5*, *-23*, *-24*, *-35*, *-39*, *-44*, and *-54* were up-regulated at 3–6 h after SA treatment. As shown in Figure 4 and Table S5, under MeJA treatment, seven genes (*MiMYB2*, *-5*, *-7*, *-22*, *-25*, *-36*, and *-54*) and two genes (*MiMYB3* and *MiMYB16*) transcription levels were up-regulation and down-regulation, respectively. However, *MiMYB21, -37, -38* were not activated during this process. *MiMYB28*, *-32*, *-34*, *-35*, and *-44* were up-regulated at 3–6 h after MeJA treatment. As shown in Figure 5 and Table S6, under H_2_O_2_ treatment, the expression levels of the *MiMYB35* and *-54* genes were up-regulated at all time periods. Five MiMYBs (*MiMYB21, -23, -25, -38,* and *-42)* were not activated during this process. *MiMYB3*, *-5*, *-6*, *-16*, *-17*, *-32*, *-36*, *-39*, *-40*, *-41*, and *-47* were up-regulated at 3–6 h after H_2_O_2_ treatment. In summary, *MiMYB5*, *-35*, *-36*, and *-54* simultaneously responded positively to early treatments (3–6 h) of SA, MeJA, and H_2_O_2_. There was differential expression of some *MiMYB* members (*MiMYB29*, *-34*, *-36*, *-39*, and *-54*) in response to SA, MeJA, and H_2_O_2_ treatments (Figure 6). These results indicated that some of the *MiMYBs* showed transcriptional changes under abiotic stress.

### 2.5. Differential Expression of MiMYBs Profiles in Response to Pathogen Infection

**Figure 7:**
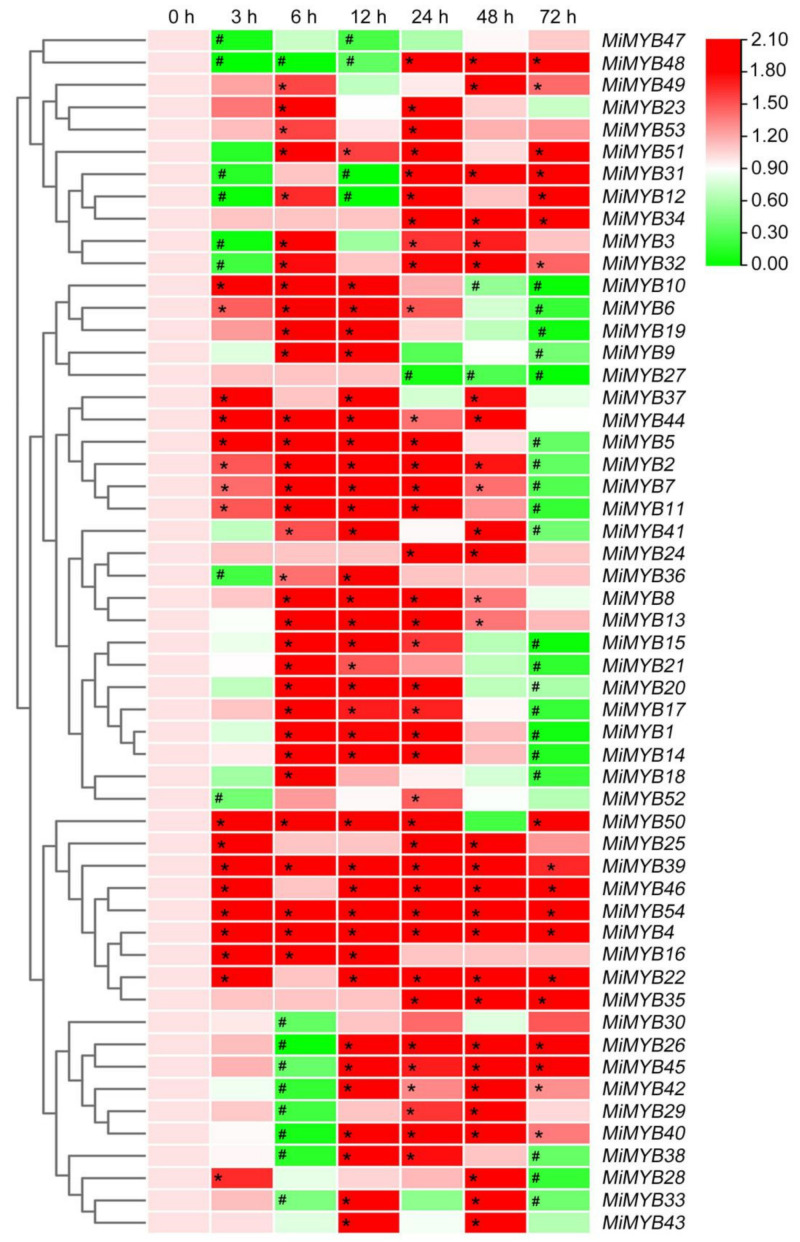
Expression patterns of *MiMYBs* in response to *C*. *gloeosporioides* infection. Relative transcript levels of *MiMYBs* with 2 × 10^6^ conidiospores/mL *C*. *gloeosporioides* infected mango leaves by spray at 0, 3, 6, 12, 24, 48, and 72 h. The relative transcript levels of *MiMYBs* in the non-sprayed mango leaves (0 h) were normalized to 1.0. The color scale represents gene expression levels with high transcript levels (red) or low transcript levels (green). The calculation method was 2^−∆∆CT^, and ‘*’ means the value exceeds 1.5, while ‘#’ means the value is below 0.5.

**Figure 8:**
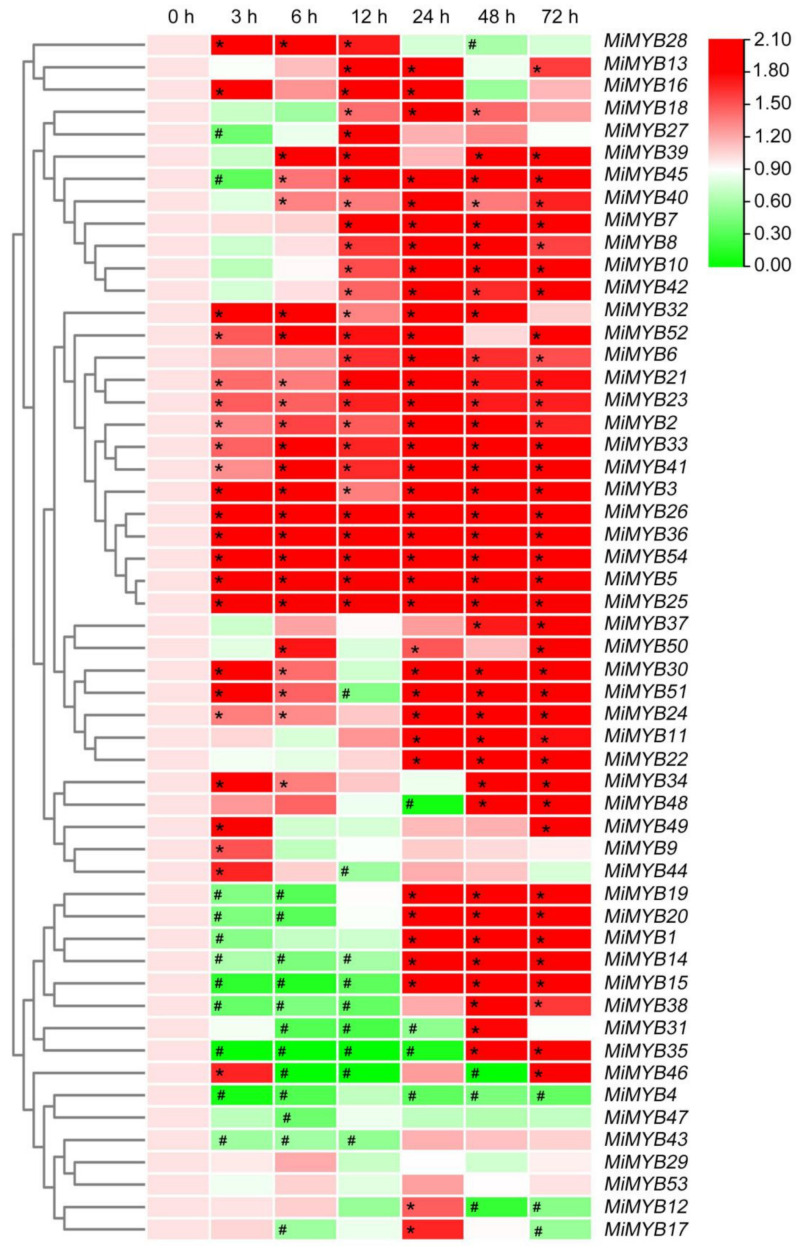
Expression patterns of *MiMYBs* in response to *X*. *campestris* pv. *mangiferaeindicae* infection. Relative transcript levels of *MiMYBs* with 2 × 10^7^ CFU/mL *X*. *campestris* pv. *mangiferaeindicae* infected mango leaves by spray at 0, 3, 6, 12, 24, 48, and 72 h. The relative transcript levels of *MiMYBs* in the non-sprayed mango leaves (0 h) were normalized to 1.0. The color scale represents gene expression levels with high transcript levels (red) or low transcript levels (green). The calculation method was 2^−∆∆CT^, and ‘*’ means the value exceeds 1.5, while ‘#’ means the value is below 0.5.

**Figure 9:**
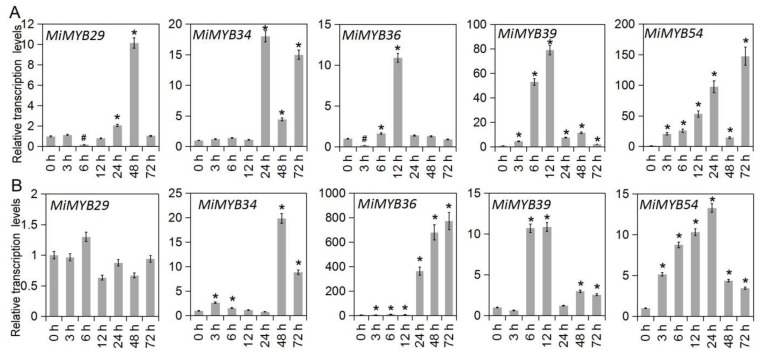
Differential expression of *MiMYB29*, *-34*, *-36*, *-39*, *-54* in response to pathogen infection. (**A**) shows the results of the *C. gloeosporioides* infection, and (**B**) shows the results of the *X*. *campestris* pv. *mangiferaeindicae* infection. The calculation method was 2^−∆∆CT^, and ‘*’ means the value exceeds 1.5, while ‘#’ means the value is below 0.5.

To investigate the possible role of *MiMYBs* in plant-pathogen interactions, qRT-PCR was used to analyze the response of mango leaves infected with *C*. *gloeosporioides* and *X*. *campestris* pv. *mangiferaeindicae* in comparison to a control. In the process of *C*. *gloeosporioides* infecting mango leaves (Figure 7 and Table S7), the expression of three genes (*MiMYB4*, *-39*, *-54*) was up-regulated overall; however, *MiMYB47* was not activated during this process. *MiMYB2*, *-5*, *-6*, *-11*, *-44*, and *-50* were up-regulated at appressoria formation on leaves. As shown in Figure 8 and Table S8, the 9 genes (*MiMYB2*, *-3*, *-5*, *-23*, *-25*, *-26*, *36*, *-41*, and *-54*) responded to the expression of *X*. *campestris* pv. *mangiferaeindicae* at all time points; however, *MiMYB29* and *MiMYB53* were not activated during this process. The transcript levels of *MiMYB54* were activated by both pathogenic fungal and bacterial infection. There was differential expression of some *MiMYB* members (*MiMYB29*, *-34*, *-36*, *-39*, and *-54*) in response to *C*. *gloeosporioides* and *X*. *campestris* pv. *mangiferaeindicae* infection (Figure 9). The results showed that *MiMYB* genes had different expression pattern responses to pathogens at different infecting times.

## 3. Discussion

The MYB transcription factor gene family is one of the largest transcription factor gene families in plants with special structures [5,21]. To date, although some studies have reported on MYB transcription factors in model plants and crops, there is still little information available on this family in the mango genome. Zheng et al. [22], Tafolla-Arellano et al. [23], and Zhang et al. [24], using different transcriptome data, analyzed for mango MYB transcription factors. Kanzaki and colleagues found that *MiMYB1* regulates the light-dependent red coloration of the ‘Irwin’ mango fruit skin [25]. Recently, large-scale data sets describing the mango genome, transcriptome, and proteome have become available [2,19,23,26,27]. In this study, 54 *MiMYB* transcription factors were identified in the mango genome (371.6 Mb) at the genome-wide level, and were named *MiMYB1* to *MiMYB54*, with 0.14 MYB per Mb genome size [2]. The phylogenetic relationships, conserved domains, motif composition, and expression profiles of *MiMYBs* were investigated systematically. In various plants, the number of *MYB* transcription factors are different; for instance, 198 *MYBs* were identified in Arabidopsis [28], 252 in *G*. *max* [29], and 524 in *G*. *hirsutum* [10]. Previous studies showed that the huge MYBs in plants are generated due to gene expansion [30]. Tandem and segmental duplications distribute and separate family members in the genome [31]. MiMYB proteins and Arabidopsis MYBs were not equally distributed into 13 different groups. In the phylogenetic tree, 54 *MiMYBs* exist in seven different groups, which indicates that *MiMYBs* are highly differentiated in the mango genome. One possible reason is that close *MiMYBs* in the phylogenetic tree are similar in MYB domains, but may not be in full-length amino acids or rest domains which may have other functions. Among them, the two largest groups (Group 1, Group 4) represented more than 33% of the total *MiMYB* members. This has been observed in sweet osmanthus (*Osmanthus fragrans*) [32] and soybeans (*G*. *max*) [29]. Members of the same group may have experienced common evolutionary origins and conserved functional domains [10]. The results for Ka/Ks further validate that most duplicated genes underwent negative or positive selection to reduce deleterious mutations, thus maintaining this gene subfamily’s members and possible expression. This is consistent with the results for other plants [33,34]. 

In general, the MYB protein has a conserved domain at the *N*-terminus, which is constituted by up to three adjacent repeats, each containing three helices. The second and third helices form the HTH structure combining cis-elements [5]. In our study, all members showed one to two MYB conserved domains at the *N*-terminus, except for *MiMYB11* and *MiMYB49*. In addition, some conserved amino acids were especially distributed in the third helix. Therefore, the conserved third helix could predict that the activity of a MYB gene bound to DNA is stable. The alterations in the third helix could result in targeting genes specifically and/or could affect DNA binding activity [32]. Most of the MiMYB proteins within the same group showed similar motif compositions, while high differences were observed between the different groups [5]. Dubos et al., expressed the opinion that groups that share similar protein motifs probably share similar functions [5]. In fact, the functions of most conserved motifs remain to be identified and described [24,35].

Gene expression patterns can provide important clues for gene function. Many *MYBs* were reported to be involved in biotic and abiotic stress responses in Arabidopsis and other plants. In our study, we analyzed the expression profiles of *MiMYBs* under various biotic and abiotic stress factors in mango. *MiMYBs* showed different expression patterns, and certain *MiMYBs* exhibited the highest expression abundance in a specific period during infestation. These *MiMYBs* may participate in specific biotic or abiotic stress responses through the regulation of different target genes. *MiMYBs* clustered in the same group did not show similar expression patterns. In this study, *MiMYB36* showed up-regulated transcription in SA stress, MeJA stress, and *X*. *campestris* pv. *mangiferaeindicae* infection, and *MiMYB54* has a certain up-regulated response to MeJA, H_2_O_2_ stress, *C*. *gloeosporioides*, and *X*. *campestris* pv. *mangiferaeindicae* infection. Thus, we speculated that these two genes participate in biotic and abiotic stress processes. A considerable amount of research demonstrated that MYB transcription factors play important roles in responses to biotic and abiotic stress [36,37]. At the early stage of *Colletotrichum* spp. conidiospores infection, appressoria formation on plants [38,39], the transcript levels of nine *MiMYB* genes (*MiMYB2*, *-4*, *-5*, *-6*, *-11*, *-39*, *-44*, *-50*, and *-54*) were up-regulated. In *Vitis davidii*, grape ripe rot pathogenic fungi (*C*. *viniferum*) invasion produces intracellular and extracellular Ca^2+^ deregulation to stimulate MYB up-regulation [40]. This led us to speculate that mango regulates the transcription of *MiMYB54* through MeJA and ROS to achieve disease-resistant immune responses. Similarly, mango regulates the transcription of *MiMYB36* through SA and MeJA, thereby achieving resistance to *X*. *campestris* pv. *mangiferaeindicae*. Evidence suggests that the transcriptional levels of *TaMYB29* in wheat [41], *FtMYB3* in Tartary buckwheat (*Fagopyrum tataricum*) [42], and *MYB34* in *Brassica oleracea* [43] were significantly induced by both SA and MeJA. *TaMYB29* positively regulates the defense response against stripe rust in wheat by H_2_O_2_ accumulation and SA-signaling-pathway-induced cell death [34]. *MYB34* is involved in the biosynthesis and breakdown of glucosinolate to resist against black rot pathogen (*X*. *campestris* pv. *campestris*) in cabbage (*Brassica oleracea* var. *capitata*) [44].

## 4. Materials and Methods

### 4.1. Identification of MYB Transcription Factor Genes in Mango

The sequences of 169 MYB proteins from Arabidopsis were downloaded from the PlantTFDB v5.0, and mango protein sequences were downloaded from the National Genome Science Data Center (BioProject: PRJCA002248, accession no. GWHABLA00000000, accessed on 7 March 2020) [19]. To identify the mango MYB members, two different approaches were used as follows: firstly, local hidden Markov model-based searches (HMMER: http://www.ebi.ac.uk/Tools/hmmer/, accessed on 7 March 2021) built from known *MYBs* to search the mango genome database; secondly, BLAST analyses with all the Arabidopsis *MYBs* as queries were employed to check the predicted *MYBs* in the mango database. With the help of the CDD (http://www.ncbi.nlm.nih.gov/cdd/, accessed on 7 March 2021) and PFAM databases (http://pfam.sanger.ac.uk/, accessed on 7 March 2021), the potential mango *MYB* members identified from HMM and BLAST searches were only accepted if they contained the MYB domain, and then multiple sequence alignments were used to confirm the conserved domains of predicted *MYB* sequences. ExPASy (http://web.expasy.org/protparam/, accessed on 7 March 2021) was used to calculate the number of amino acids, molecular weights (MW), and theoretical isoelectric points (pI) of MiMYB proteins. The MEME (http://meme-suite.org/tools/meme/, accessed on 7 March 2021) program was used to identify the conserved motifs in MiMYB protein sequences. The motif distribution type was set as zero or one occurrence per sequence. The number of motifs was set as 15, and the motif width was between 6 and 50 amino acids. These data were integrated and visualized using TBtools [45].

### 4.2. Multiple Sequence Alignments, Phylogenetic Analysis, Ka/Ks Calculation, and Synteny Analysis

The protein sequences of MYB proteins from mango and Arabidopsis were aligned by the ClustalX program and adjusted manually, and the multiple sequence alignments were used for phylogenetic analysis. Phylogenetic trees based on protein sequence alignments of *MYBs* from mango and Arabidopsis were constructed by the neighbor-joining method with 1000 bootstrap replicates in MEGA 7.0 (http://www.megasoftware.net/download_form/, accessed on 7 March 2021), and grouped according to genetic distance. of the phylogenetic tree was optimized using iTOL (https://itol.embl.de/#/, 7 March 2021). In order to analyze and calculate the pattern of gene duplication in *MiMYBs*, the non-synonymous (Ka) and synonymous substitutions (Ks), and evolutionary rates (Ka/Ks) in their paralogs and orthologs were evaluated using TBtools software [45] and MEGA 7.0. Ka/Ks < 1, Ka/Ks = 1, and Ka/Ks > 1 generally indicate negative, neutral, and positive selection, respectively [33,46]. The circle map of synteny analysis was performed for MiMYB genes in mango genome using TBtools software [45].

### 4.3. Plant Materials, Growth Conditions, and Treatments

One-year-old mango seedlings (Guifei mango) were cultured in pots containing well-mixed soil (soil:vermiculite, 3:1) in a greenhouse in Haikou, China. The newly grown tender leaves were cut out separately and quick-frozen in liquid nitrogen, stored at −80 °C, and used as a 0 h control sample. For biotic and abiotic stress treatment [47,48,49], the mango seedlings were treated with sprinkler irrigation, which included *C. gloeosporioides* (*Cg*, 2 × 10^6^ conidiospores/mL) and *X. campestris* pv. *mangiferaeindicae* (*Xcm*, 2 × 10^7^ CFU/mL), and salicylic acid (SA, 5 mmol/L), methyl jasmonate (MeJA, 5 mmol/L), and hydrogen peroxide (H_2_O_2_, 10 mmol/L), respectively. Each treatment was set to 3 repetitions, and samples were taken at the treatment time intervals of 3, 6, 12, 24, 48, and 72 h separately and quick-frozen in liquid nitrogen, then RNA was isolated and stored at −80 °C.

### 4.4. RNA Extraction and qRT-PCR Analysis

RNAprep Pure Plant Plus Kit (Tiangen, Beijing, China) was used to extract total RNA from mango leaves, determine the concentration of total RNA, and check its quality. FastKing RT Kit (with gDNase) (Tiangen, Beijing, China) was then used for reverse transcription to generate first-strand cDNA, and stored at −80 °C.

The gene-specific primer sequences were designed by Primer 5.0 software, Primer3Plus (https://www.bioinformatics.nl/cgi-bin/primer3plus/primer3plus.cgi, accessed on 7 March 2021) [50] and listed in Table S3, Text S1. Using cDNA as a template, QuantStudio 6Flex real-time fluorescent quantitative PCR was used to detect the expression of *MiMYBs* under different pathogen infections and treatments. The reaction program is 95 °C pre-denaturation for 10 min, 95 °C denaturation for 15 s, 60 °C annealing extension for 1 min, fluorescence signal collection, a total of 50 cycles; after the end of the cycle, when heating from 60 °C to 95 °C for melting curve analysis, *MiActin* [51] was employed as a reference gene. The expression level at 0 h was used as a control. The 2^−∆∆Ct^ method and SPSS software were used to perform data statistics and variance analysis on the Ct value of each sample, calculate the relative gene expression levels of *MiMYBs*, and visualize the genes’ expression heat map with TBtools software. Relative expression levels greater than 1.5-fold (1.5-fold higher than control) were considered up-regulated, whereas relative expression levels that were less than 0.5-fold (0.5-fold lower than control) were considered down-regulated.

## 5. Conclusions

In this study, 54 *MiMYB* transcription factors were identified from the mango genome and classified into 7 group, including Groups 1, -3, -4, -5, -6, -8 and -9, with high similarities in the MYB domain and motif composition within the same group. Quantitative real-time PCR showed that the transcription levels of *MiMYBs* were different under abiotic and biotic stresses, including SA, MeJA, and H_2_O_2_ treatments, and *C*. *gloeosporioides* and *X*. *campestris* pv. *mangiferaeindicae* infection, respectively. The transcript levels of *MiMYB5*, *-35*, *-36*, and *-54* simultaneously responded positively to early treatments of SA, MeJA, and H_2_O_2_. The transcript levels of *MiMYB54* were activated by both pathogenic fungal and bacterial infection. These results will be beneficial for future interested researchers aiming to understand the biological functions and molecular mechanisms of *MiMYBs*, which could be further confirmed through functional characterization by overexpression and gene silencing. In addition, the results will be of interest to scientists working on transcription factor studies of mango and tree crops, as well as to the wider plant research community.

## Data Availability

Not applicable.

## Supplementary Materials

The following are available online at https://www.mdpi.com/article/10.3390/plants11223141/s1: Table S1. Detailed information of MiMYBs genes in mango; Table S2. The Ka, Ks and Ka/Ks values of MiMYB genes; Table S3. Primer sequences for qRT-PCR; Table S4. The expression data of MiMYB genes response to SA treatment; Table S5. The expression data of MiMYB genes response to MeJA treatment; Table S6. The expression data of MiMYB genes response to H2O2 treatment; Table S7. The expression data of MiMYB genes response to C. gloeosporioides infection; Table S8. The expression data of MiMYB genes response to X. campestris pv. mangiferaeindicae infection; Figure S1. The circle map of synteny analysis was performed for MiMYB genes in mango genome by TBtools software; Figure S2. MiMYBs of agarose gel electrophoresis of products amplified by qRT-PCR; Text S1. Nucleotide sequences of 54 MiMYB; Text S2. Amino acid sequences of 54 MiMYB.
