# Supplementary material for: Genome-Wide Characterization, Identification and Expression Profile of MYB Transcription Factor Gene Family during Abiotic and Biotic Stresses in Mango (Mangifera indica)
Source: Plants (Basel). 2022 Nov 16;11(22):3141. doi: 10.3390/plants11223141 (PMC9699602; doi:10.3390/plants11223141)
Supplement: Supplementary file 1 [file plants-11-03141-s001.zip › Supplementary Materials of MiMYB-Table S1, Table S3, Figure S1,S2.pdf]

**Table S1. The detailed information of *MiMYBs* genes in mango**

| Gene name | Gene ID | Number of amino acids | DNA length | Molecular weight (kDa) | Theoretical pI | exon | sense/ antisense |
|---|---|---|---|---|---|---|---|
| MiMYB1 | GWHPABLA027279 | 957 | 3947 | 104.17 | 5.02 | 8 | - |
| MiMYB2 | GWHPABLA027993 | 320 | 1060 | 34.69 | 8.25 | 2 | - |
| MiMYB3 | GWHPABLA028858 | 902 | 6893 | 98.97 | 5.45 | 11 | - |
| MiMYB4 | GWHPABLA000111 | 369 | 1360 | 40.78 | 6.39 | 3 | + |
| MiMYB5 | GWHPABLA000367 | 215 | 899 | 24.92 | 6.62 | 2 | + |
| MiMYB6 | GWHPABLA000787 | 306 | 1185 | 34.27 | 9.27 | 2 | - |
| MiMYB7 | GWHPABLA001571 | 514 | 4881 | 57.23 | 7.87 | 8 | + |
| MiMYB8 | GWHPABLA001575 | 367 | 1638 | 41.99 | 9.22 | 2 | - |
| MiMYB9 | GWHPABLA001647 | 409 | 2281 | 44.97 | 5.41 | 3 | - |
| MiMYB10 | GWHPABLA001674 | 308 | 1370 | 35.04 | 8.76 | 2 | - |
| MiMYB11 | GWHPABLA001867 | 615 | 1932 | 70.31 | 8.69 | 2 | + |
| MiMYB12 | GWHPABLA002362 | 463 | 3408 | 50.74 | 5.13 | 3 | - |
| MiMYB13 | GWHPABLA004872 | 1121 | 6776 | 125.84 | 6.15 | 10 | - |
| MiMYB14 | GWHPABLA004942 | 236 | 1563 | 27.90 | 7.70 | 3 | + |
| MiMYB15 | GWHPABLA005064 | 314 | 1466 | 33.91 | 6.88 | 2 | - |
| MiMYB16 | GWHPABLA005313 | 435 | 2297 | 49.64 | 6.58 | 3 | + |
| MiMYB17 | GWHPABLA006538 | 377 | 2347 | 42.72 | 7.12 | 3 | - |

| | | | | | | | |
|---|---|---|---|---|---|---|---|
| MiMYB18 | GWHPABLA007103 | 1062 | 7778 | 116.78 | 6.22 | 2 | + |
| MiMYB19 | GWHPABLA007174 | 308 | 2277 | 35.38 | 9.28 | 3 | - |
| MiMYB20 | GWHPABLA007175 | 275 | 1417 | 30.98 | 7.07 | 2 | - |
| MiMYB21 | GWHPABLA007189 | 457 | 3598 | 49.49 | 5.62 | 2 | + |
| MiMYB22 | GWHPABLA008377 | 300 | 1058 | 33.78 | 9.65 | 2 | + |
| MiMYB23 | GWHPABLA009654 | 227 | 1027 | 25.23 | 9.26 | 2 | - |
| MiMYB24 | GWHPABLA009746 | 345 | 1252 | 37.89 | 6.71 | 2 | + |
| MiMYB25 | GWHPABLA010233 | 312 | 1256 | 35.58 | 9.67 | 2 | - |
| MiMYB26 | GWHPABLA011031 | 473 | 4360 | 53.35 | 6.45 | 12 | + |
| MiMYB27 | GWHPABLA011054 | 399 | 1725 | 44.84 | 6.24 | 3 | - |
| MiMYB28 | GWHPABLA011331 | 347 | 5875 | 37.48 | 8.62 | 4 | - |
| MiMYB29 | GWHPABLA012350 | 283 | 1041 | 32.90 | 5.79 | 3 | - |
| MiMYB30 | GWHPABLA012843 | 313 | 1348 | 35.53 | 6.11 | 3 | - |
| MiMYB31 | GWHPABLA013290 | 382 | 2970 | 43.36 | 7.64 | 5 | + |
| MiMYB32 | GWHPABLA013390 | 197 | 764 | 22.64 | 5.78 | 3 | - |
| MiMYB33 | GWHPABLA013713 | 317 | 1427 | 34.23 | 6.84 | 2 | + |
| MiMYB34 | GWHPABLA020355 | 383 | 1416 | 42.36 | 6.85 | 3 | - |
| MiMYB35 | GWHPABLA020548 | 404 | 2570 | 46.23 | 8.81 | 2 | - |
| MiMYB36 | GWHPABLA022091 | 376 | 1378 | 41.68 | 8.69 | 3 | + |
| MiMYB37 | GWHPABLA022650 | 307 | 1238 | 34.43 | 9.19 | 2 | + |
| MiMYB38 | GWHPABLA023284 | 980 | 5344 | 110.55 | 5.30 | 4 | + |
| MiMYB39 | GWHPABLA024160 | 413 | 1431 | 46.82 | 5.88 | 3 | + |

| | | | | | | | |
|---|---|---|---|---|---|---|---|
| MiMYB40 | GWHPABLA030438 | 556 | 3478 | 60.68 | 4.91 | 3 | + |
| MiMYB41 | GWHPABLA030681 | 974 | 3034 | 106.95 | 5.57 | 2 | + |
| MiMYB42 | GWHPABLA030846 | 282 | 1259 | 31.81 | 8.09 | 2 | + |
| MiMYB43 | GWHPABLA031111 | 558 | 3480 | 61.03 | 4.87 | 3 | - |
| MiMYB44 | GWHPABLA031168 | 247 | 1681 | 28.04 | 5.89 | 2 | + |
| MiMYB45 | GWHPABLA032359 | 477 | 4251 | 53.94 | 8.70 | 12 | - |
| MiMYB46 | GWHPABLA032392 | 401 | 1727 | 44.94 | 8.80 | 3 | - |
| MiMYB47 | GWHPABLA032959 | 271 | 1151 | 30.45 | 5.28 | 2 | + |
| MiMYB48 | GWHPABLA033543 | 777 | 9203 | 84.41 | 8.54 | 6 | - |
| MiMYB49 | GWHPABLA015853 | 613 | 1933 | 70.10 | 8.75 | 2 | - |
| MiMYB50 | GWHPABLA015906 | 367 | 1933 | 39.73 | 5.47 | 2 | - |
| MiMYB51 | GWHPABLA016167 | 380 | 1231 | 43.94 | 9.33 | 2 | + |
| MiMYB52 | GWHPABLA016177 | 522 | 3781 | 57.70 | 8.44 | 7 | - |
| MiMYB53 | GWHPABLA019417 | 946 | 5176 | 106.70 | 5.38 | 5 | - |
| MiMYB54 | GWHPABLA019508 | 382 | 1355 | 42.13 | 8.70 | 3 | + |

**Table S3. Primer sequences for qRT-PCR**

| Gene name | Forward primer（5′-3′） | Reverse primer（5′-3′） |
|---|---|---|
| *qMiMYB1* | CATGCTCTCCAGCAATTTCA | CCATGCTGAAGGGGATTCTA |
| *qMiMYB2* | CGATGGCAGAGGATGGTAAT | ATGGACTACTCGGGTTCACG |
| *qMiMYB3* | TCCTTCAAAGCCTGAAGCAT | GCATCCTCTGAAGGACAAGC |
| *qMiMYB4* | GGATCCGAAGATCGACAAAA | GCCATTACAACATGCCAGTG |
| *qMiMYB5* | GGGAAAGGAGATTGGAGGAG | TAGGTTCAGTGGCCCATTTC |
| *qMiMYB6* | CAGGTGGCTAGTCATGCTCA | TATTTGGCTGATGCCATTGA |
| *qMiMYB7* | CTCGGAATCTATGCCAAAGC | CCCTTCACACAAGGTGGACT |
| *qMiMYB8* | GTTGAGAAGGCTGGAGTTGC | TCAGCCAATTTCTGCTCCTT |
| *qMiMYB9* | GTCGGCTCAGGTGGTGTAAT | CCTAGCAATGACAGCCCATT |
| *qMiMYB10* | ATCTCCCGCAATTTTGTGAC | TCAATTTTGGTTGCTGTTGC |
| *qMiMYB11* | ATGGATGGAGAGGCAGAATG | TCTTCTTCAGCAGCCCTAGC |
| *qMiMYB12* | GAGTGTGTCGAGCAGTGGAA | TTGGCCTTGCTCATTCTCTT |

| | | |
|---|---|---|
| *qMiMYB13* | AGGAGGAAAGGGAAGCCATA | CGTCTTCGCTATCGGAACTC |
| *qMiMYB14* | CAGAAGAGCGGCTCAAAATC | CACCCGCTGGTTCTTGTTAT |
| *qMiMYB15* | AGTGGCGAGAAGAGGTCAAA | AGCAACTTGTGTTGGGGTTC |
| *qMiMYB16* | AGACTCGACTCCCAAAAGCA | GACTTGCCAATCAGCTGTGA |
| *qMiMYB17* | CGGAAGATGAAAACGACGAT | TTTCCATCGTTCCCAGTCTC |
| *qMiMYB18* | ATGCTGCTGATGACACTTGC | AACCTTGCATTGATCCTTGG |
| *qMiMYB19* | ACCATTGGTGAAAAGCTTGG | TGAGCATAGCTTGCCACTTG |
| *qMiMYB20* | AGCTGGACCTGAATGGAATG | GATTTCCGGGTTGAACTGAA |
| *qMiMYB21* | TCGACTGGTGAGTCAGTTCG | GCTATTGCTGCCCATTTGTT |
| *qMiMYB22* | ATGGCGAGAAAGCAAAGAGA | GCGAAAGTCCAAGAAAGCTG |
| *qMiMYB23* | GACCCGTCTTGTTGAACGAT | GCCAAGATGGTCTCGTCTTC |
| *qMiMYB24* | CTACGGTCCACGAAACTGGT | TAAAAGACGGGCGATGGTAG |
| *qMiMYB25* | CCGTTTCTCAGCTTTCTTGG | AAAGAGCCAAATGGACGTTG |
| *qMiMYB26* | TGAGGACTCTCCAGCCAGTT | CAATGTTGCTCGCCAATATG |
| *qMiMYB27* | ATGACCAGTGGGCAAGAAAC | TGAAGTTGGTGGTGTTGGAA |
| *qMiMYB28* | CAGCGTTAGCAATGGAGACA | CTATTGCGGTATGCCATGTG |
| *qMiMYB29* | GTTACGTTTGGATGCCTCGT | ACTGAGTCCCAGGAAGAGCA |
| *qMiMYB30* | TAGGCAAAAGAGGCTGGCTA | CTGTTGGATGACGAGCAAGA |
| *qMiMYB31* | GCATGCAGAGATAGGCAACA | GTTTTCGCCTGATGTTTGGT |
| *qMiMYB32* | ACCGTTGCTACCAAATCAGG | AATTTCGTTGTCCGTTCGTC |
| *qMiMYB33* | AGGGAGATTGGAGGAGCATT | GTTTCCCTGTTGCCCTGTTA |
| *qMiMYB34* | GATCCTGATGGGTTCGAAGA | CTCCCTTGCAACTTCTCAGC |

| | | |
|---|---|---|
| *qMiMYB35* | CGAAAGCAAAGAGGCAAAAC | CCCAGTGCCCAGTGATTAGT |
| *qMiMYB36* | ACCGGAGGACATTCAATGAG | CACTGGTGGCATTTTTGTTG |
| *qMiMYB37* | CTCCTTCCCCAGACAATCAA | GGCATTTTTAGCCCATACGA |
| *qMiMYB38* | ATGATAAAGGGAGGCGTGTG | TTTTTGATGGAGGGATCGAG |
| *qMiMYB39* | CGACTGGTTGAACAACATGG | CTTCCAGGCAATCTCTTTGC |
| *qMiMYB40* | TATGTTGGGTGCAAGGTGAA | TGCTGTCGAAGTTCCTGTTG |
| *qMiMYB41* | TCATCCAAAATGGAGCATCA | GGCTGTGGGAGAGTTGAGAG |
| *qMiMYB42* | GTGCTTCCACAGCAACAGAA | TCGGGGTTTGAAATGAACTC |
| *qMiMYB43* | CCAAAATGGGAAACAAATGG | CTGCAATGCTTGGAAAGACA |
| *qMiMYB44* | CGCGATGTCAAGGAACACTA | CCAGGGAGTACCCTTCTTCC |
| *qMiMYB45* | GCCAATGGAGAATCCAAGAA | TCTTCCTCAGGTGACCATCC |
| *qMiMYB46* | ATGTTCAGCCAGGTGAGGTC | GCTCCTACCCCGAGAAAATC |
| *qMiMYB47* | TGTTGGGACGTTTGTTGAAA | CCAAGGGAGGTTTCACAGAA |
| *qMiMYB48* | AATTCACCGATTTGGACTGC | GCCAACACAGCCTTTACCAT |
| *qMiMYB49* | AGCCACTTTAAGGCAGTCCA | CCACCTTCCGTTCCTCATAA |
| *qMiMYB50* | GGCTGTCATTGCTAGGCTTC | AACGAATTTGCATCACCACA |
| *qMiMYB51* | CATGTCGAAGGAGGGAAAAA | ACACCGATTATTGGGCTCTG |
| *qMiMYB52* | AATGGGCTGAAATTGCAAAG | CCTGATGAAGTTTGGGCAGT |
| *qMiMYB53* | GGAAGGGAAGAGGAGGATTG | TGCAGGAAGCATCAGTTTTG |
| *qMiMYB54* | GGATAACAAGGGGAAGCACA | GCATTCTCATTTTCCCCAGA |
| *qMiActin* | GTTTCCCAGTATTGTGGGTAGG | AGATCTTTTCCATATCATCCCAGTT |

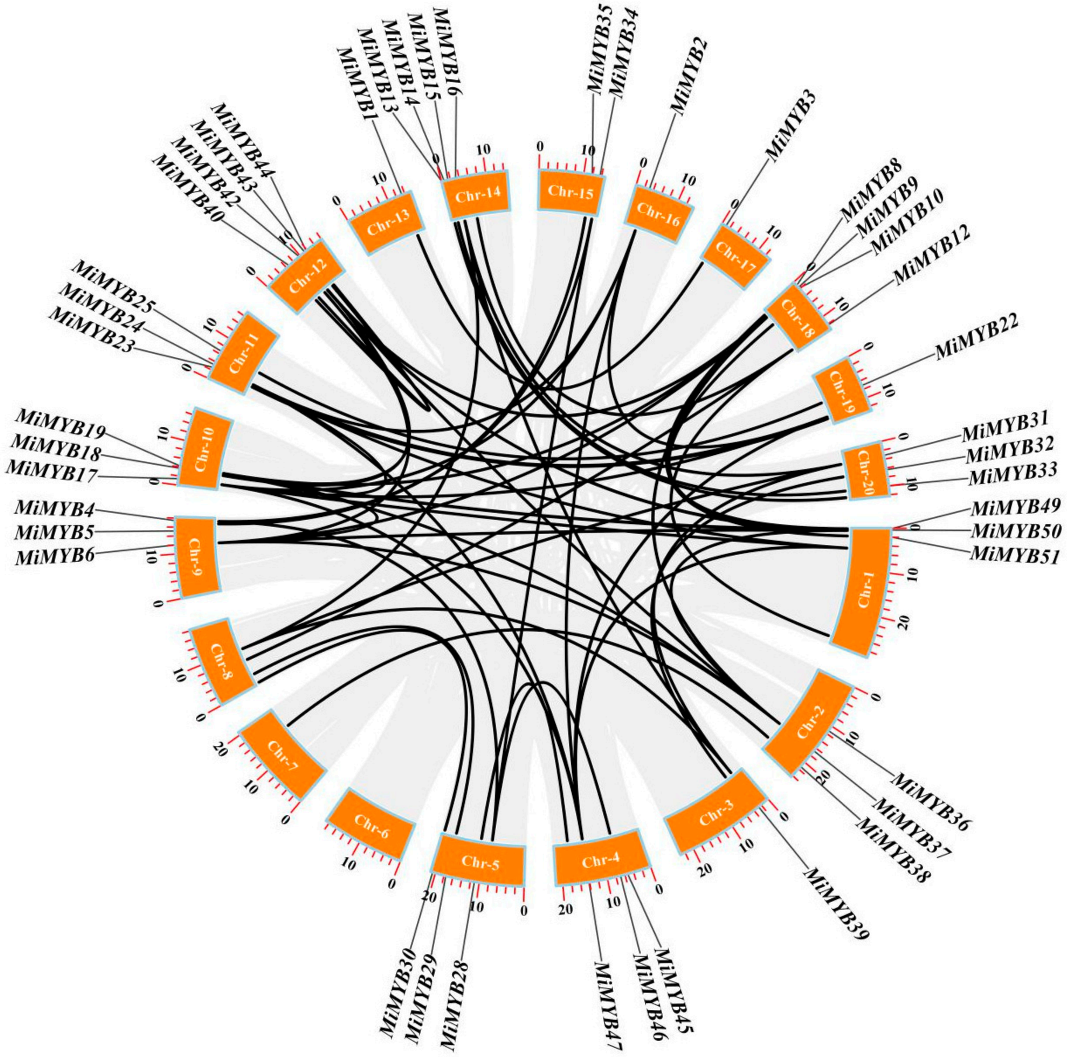


Figure S1 The circle map of synteny analysis was performed for MiMYB genes in mango genome by TBtools software.

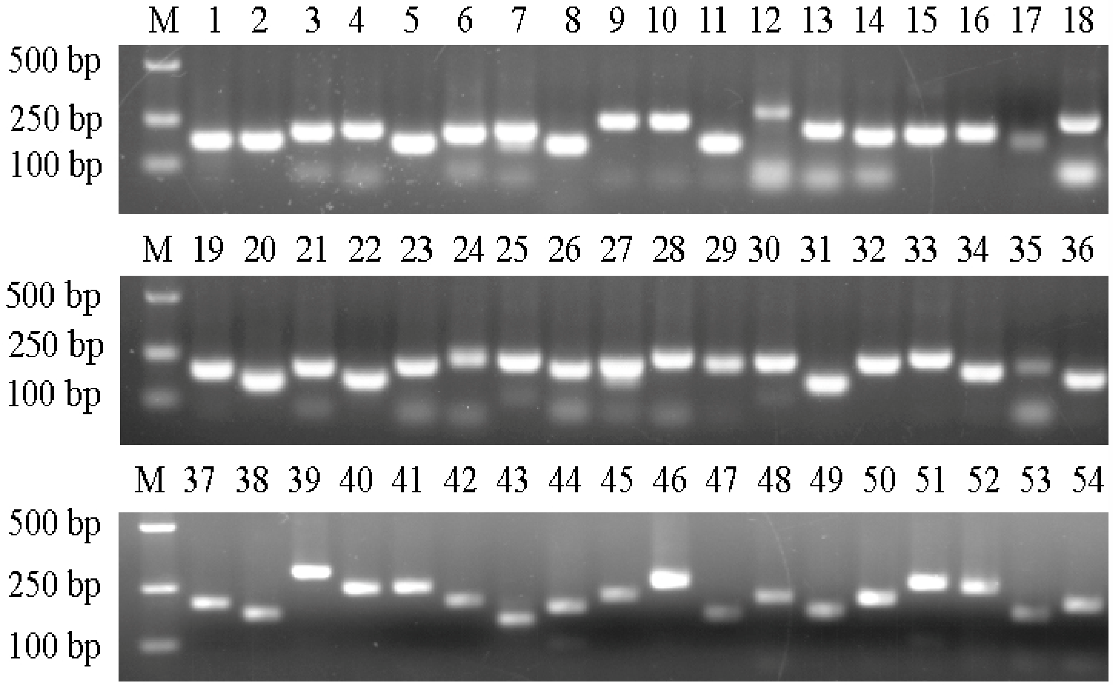


Figure S2 *MiMYBs* of agarose gel electrophoresis of products amplified by qRT-PCR. M represents marker 2000 DL, and members 1—54 represent *MiMYB1*—*MiMYB54*.
