# Supplementary material for: Genome-Wide Characterization, Identification and Expression Profile of MYB Transcription Factor Gene Family during Abiotic and Biotic Stresses in Mango (Mangifera indica)
Source: Plants (Basel). 2022 Nov 16;11(22):3141. doi: 10.3390/plants11223141 (PMC9699602; doi:10.3390/plants11223141)
Supplement: Supplementary file 1 [file plants-11-03141-s001.zip › Text S1 Nucleotide sequences of 54 MiMYB.pdf]

**Text S1 Nucleotide sequences of 54 MiMYB**

In the Text S1, the green, highlighted sequences are qRT-PCR primer sequences by Primer 5.0 software and online Primer3Plus (https://www.bioinformatics.nl/cgi-bin/primer3plus/primer3plus.cgi)

>MiMYB1

ATGGAGGATGAAATAATAATAGAATTGGTGAACAAGTTTGGACCAAAAAAGTGGTCCACGA
TTGCTCAGCATTTACCAGGTCGAATTGGAAAACAATGCAGGGAAAGGTGGCATAATCACCT
TAACCCCTCCATAAACAAAGAGGCATGGACTCAAGAAGAAGAGTTGGCTTTGATTCGTGCA
CATCAGATTTATGGGAACAAATGGGCGGAGCTAACAAAGTTCTTGCCAGGAAGGACAGAC
AATGCTATAAAAAATCACTGGAACAGCTCTGTGAAAAAGAAATTGGAGTCTTACTTGGCAT
CAGGATTATTAGAGCAGTTTCAGGGCCTGCCTCTTGTTGGGCATCAGAATCAACCTGTGCCC
TCATCATCTTTGGGGATGCAGAGCAGTGGAGATGACAGTTGTCCTAAAGGTGGAACTGCAG
CTGAGGAAATATCAGAATGCAGCCAAGATTCAAATGTTCTAGGTCTCTCTCATTCAACAGGT
AATGCAGTTATGCATACAAGAGAGCAGCTCCTTGAAACTGAAGAGTCCAATCCACCCAAGG
ATCACAGCTCCAGCCCAGCATCTTGTTCAGAACAATATTACACATCTCTGGAAGATGTCACG
TTTTCCATCCCTGAAATACCTTGTGAAGTGGGTTGCTCTTCTAAGTTCCTTGAGCAAAATTTT
GTGCATGATGCTGGGACTTTTGCTGGGGATTACCAGTTCAATTTACAGGATTTACCTAATGTG
TCTTCCCTGGGCTGGGGGCATCAATCATTGGGGTTGCCAGCTAATTGTATGGATTCTCATGA
AAGTCATGAAATGGTCAATGTTCCATATCAAAATTCCACGGAATTGAGTGTTCCTAGCTCTAT
AGGAAATCTAGCTGCAGGTTCTGATAAAGCAGAGCACATCTTGATAACAGATGATGAATGTT
GCAGGATCTTGTTCACAGAGGCAATGAAAGATGGATGTTTTTCCTTGGAAAATTCTGCACA
AGGACTGAATATGGTTGAATCTTCACTTTGCCGCTCTCTGGGCATTCAGATTTCTGAAGCTG
GCAGAACCTCACCTTCCCAAGCTTATTGTCCTTCAAAGCCTGAAGTATTAGGGACTTCATGT
AGTCAATCTTTTCTATCTGCTCCAATGGTACTTTCAACTGACAATGGTGCATTTGTATATGTT
GGTGAATCTAGTGAGTTGAATTGCCACACCTATGGAACCCAAGATCAAGGTTTCATTACAA
GTGGGTATGATGCCTTTGTCTATACCAATGAATCTTCCAATTCCCCTTCTGATGATGGTAAGG
ATGTTTTAGGCCAGCAAGAGCCATCACATCTTTCAAAGGATTCTTTGAAACTAGTTCCTGTA
AATAGTTTCAGTTTGAGATCAGATGCCATGATAGCTTATTCTTCAGAGGATGCCAAACCCAA
CACACAAACAGAACAGCAGGATGCAGGATCCCTATGTTATGAACCTCCTCGTTTTCCAAGT
TTGGATATTCCTTTTTTCAGTTGTGATCTTATACAATCTGGTAGTGATACACAGCAAGAATAC
AGTCCGCTTGGCATACGTCAGCTAATGATGTCATCAATGAGCTGCATTACTCCATTTAGATTA
TGGGATTCTCCATCTCTGGATGACAGCCCTGAAGCTGTGCTGAAAAGTGCTGCTAAAACTT
TTACAGGAACACCATCCATATTGAAGAAACGACACCGGGAGTTGTTGTCACCTTTGTCAGA
TAGAAGGAACGATAAAAAGCTTGAAATTGACATGACTTCCAGTTTGGCCCGGGATTTTTCT
CGTCTAGATGTCATGTTTGATGAGGCTGGTGGGATCCACAGAGCATCTTTGCTGTCTCCATT
GTCAAACCAAAAGAGGAACTCTGGAGCTTTCATTGACGAGAAGGAAAATTTGGGTCATGC
TTTTGATGGTGGACAAGAAAAGGAAAAAGATCTGTCTGCAGTCTCACATGACAGAACTTCT
GAGAAAGATTGTCTTTGCAGTAATTCCCATGCCAATGAAACGAAAGATTCTGTTGATATTGA
TTCTAAGACCAATGCTAATGCTGTTGCCACTACCAAAACTGAGAAGCAACCTGCAGGAGTC
CTTGTTGAACATAATGTGAATGATCTGCTATTTTCTCCTGATCAAACTGGTTTAAAAGCTGAA
AGATCACTGGCCACTGCTGCTCGAACACCAAGAAATCAGTATTTTAAAAGCTTTGGAGCTA
ATCAAGGTGCTGCACCCGAACAATCACCTGCAATTGCATGCTCTCCAGCAATTTCAAAAAAT
AACAATGGGGGTAATTCTAGCGGAATTAAGACAGTAAGACCCATTCCTTCATTGGCACCACC
AGGAAGTACGGTTCTTAATGCTGGGAGTGATGTTGGCATCGAAAACTATAACATTTTTGGTG
AAACTCCATTCAAGAGAAGTATAGAATCCCCTTCAGCATGGAAATCCCCTTGGTTTATGAAC
GCTTTCATGCCTGGTCCAAGGATTGACACTGATATAACAATTGAGGATATTGGTTATTTTCTG
AGCCCAGGAGAAAGAAGCTATGATGCCATTGGATTGATGAAACAGTTGAGCGAGCACACT
GCTGCTGCATATGCTGATGCCTTGGAGGTCTTGGGAAATGAAACTTCTGAGAAAACAGTGA
ACGAAGGAAACAGTAAAAAATCAAGTTTGGATGAAGAGAACAATGGTGTCACGCATAGTG
AGCACAAGAGCGACCATTTGGCTTCAACTATTTTGGTGGAGCGGCGTACCCTTGACTTCAG
TGAATGTGGAACACCTGGGAAAGGAAAAGAGAATAGGAAATCCTCAACTGCCACAGATTT
CTCAAGCCCCTCCTCATATCTATTGAAAGGTTGCAGGTAG

>MiMYB2

ATGGCGTTTAATAGAAAAGAAGTGGATCGGATCAAGGGCCCGTGGAGCCCAGAGGAGGAC
GAGGCGTTACAACAGCTTGTGCAAAAGCATGGCCCAAGAAACTGGTCGTTGATAAGCAAG
TCAATTCCGGGGCGATCCGGCAAGTCTTGCCGTCTCCGGTGGTGCAATCAGCTCTCTCCGC
AAGTTGAGCACCGCTCCTTCACACCTGAAGAAGACGAGACTATATTACGCGCTCATGCTCG
CTTCGGCAACAAGTGGGCGACAATCGCCCGGCTTCTCAACGGCCGTACAGACAACGCGAT
AAAAAACCACTGGAACTCGACCTTGAAACGGAAGTGTTCAGGTTCGATGGCAGAGGATGG
TAATTTTATCAACTGTAATGGGTACGACGGGGATTTAGGTCATAATAATCAAAATCAGCCGTT
GAAAAGATCTGTAAGTGCAGGATCAGGTGTGCCGCTGACCACGGGGCTTTACGTGAACCC
GAGTAGTCCATCTGGATCTGACGTAAGCGATTCCAGCGTACACGTATTTTCGTCGAATCACA
ACATATTTAAACCCGTTGCGAGAGCCGGCGGTGTTAATGTGGATGTGAATGTAATCCCCGAA
ACGACATCGTCGAGCAACGATCCGCCAACTTCACTGAGTCTGTCACTTCCAGGCGCCGATT
CAAGCGAAGATACGAACCAGAAGGCAGCGGAGTCAAGTAAGGCTATAAATACTAAGAGTT
TGTTCCCTGAGAGTGGAGCGAAAGGCGGTGGCTTTCTAGGGTTTAGTCAAGAGTTCATGGG
AGTGATGCAGGAGATGATAAGGCAGGAGGTGAGGAATTACATGGCTTCCCTTGAACAACAG
AGAGGTGGAGTTTGTTATCAGAGTAGTGGCGGTGGCGGCGCCGACGGTGTCTTTAGAAATA
GAGGTAGAGGTGGGCAATGGATGATAACTGAGTTAGGGGCTTTAGATTGA

>MiMYB3

ATGCGCACCGTTTTGTTCTCTGCTCCACAACCGACTTCTTCTAAATCTCGAATCTCGAAGCT
AAGTAAGGAGGATGAAATAATAATAGAATTGGTGAACAAGTTTGGGCCAAAAAAGTGGTCC
ACAATTGCTCAGCATTTACCAGGGCGAATTGGGAAGCAATGCAGGGAAAGGTGGCATAATC
ATCTTAACCCCTCCATAAACAAAGAGGCATGGACTCAAGAAGAGGAGCTGGCTTTGATTCG
TGCGCATCAGATTTATGGGAACAGATGGGCAGAGCTAACAAAGTTCTTGCCAGGAAGGACA
GACAATTCCATAAAAAATCACTGGAACAGCTCTGTGAAAAAGAAATTGGATTCTTACTTGG
CATCAGGACTACTAGAGCAGTTTCAAGGCCTCCCTCTTGCTGGGCATCCTAATCAACCCATG
CCCTCATCGTCTTCCAGGATGCAGAGTAGTGGAGATGACAGTTGTCCGAAAGGTGGAGTTG
AAGCTGAGGAAATATCAGAATGCAGTCAAGATTCGAATGTTCTTGGTCCCTCTCATTCAACA
GGTAATGTAGTTCTGCATACAAGAGAACAGGTCCTTATACCTGAAGAGTGCAATCCAGCTAA
GGATCAGAACTCCAGCCCAGCATCTTGTTCAGAACAATATTACACATCTCTGGAAGATGTCA
CATTTTCCATCCCAGAAATACCTTGTGAAGTGGGTGGCTCTTCTAAGTTCCCGAGCAAAATT
TTGTGCATGATGCTGGGACTTTTGCGAAGAGTTACCAGTTCTGATAAAACAGAGAACATCTT
GATAACAGATGATGAATGCTGCAGGGTCTTGTTTGCAGAGGCAATGAAAGACGGATGTTTT
TCCTTGGAAAATTTTGGTCAAGAGTTGAATATGGTTGAATCATCACATTGTCACCCCTTGGA
CATTCAGATTTCTGAAGGTGGCAGAACCTCACTTTCCCAAGCTTATTGTCCTTCAAAGCCTG
AAGCATGCAAGGCCTTTATCTATACCAATGAATCTGCCAATTCCCCTTGTGATGATGGTGCA
GATAATTCAGGCCTGCAAGGGCCATCATATCTTTCAAAGGATTCTTTCAAACTAGTTCCTGTA
AATAGTTTCAGTTCAAAATCAGATGCCATGATAGCTTGTCCTTCAGAGGATGCCAAGCCCAA
CATGCAAACAGAACATCAGGATGCAGGATCCCTATGTTATGAACCTCCTCGTTTTCCAAGTT
GGGATATTCCTTTTTTTAGTTGTGATCTTATACAGTCTGGTAGTGATATGCAGCAAGAATACA
GTCCACTTGGCATACGTCAACTAATGATGTCATCAACAAACTGCATTACTCCATTTAGGTTAT
GGGATTCTCCCTCTCGTGATGATAGTCCTGAAGCTGTGCTGAAAAGTGCTGCCAAAACTTTT
ACAGGAACACCATCTATATTGAAGAAGCGACACCGTGACTTGTTGTCACCTTTGTCAGATA
GAAGAAATGATAAGAAACTTGAAATTGACATGACTTCCAGTTTGGCCCAGGATTTTTCTCGT
CTGCTGTCTCCATTGTCAAACCAAAAGAGGAAATCTGGAGCCTTCGTTGAAGATAAAGAAA
ATTTGGGTCATGCTTTTGATGGTGGACAAGAAAAGGATAAAGATCAGTCTGCTGTCTTGCAT
GAAAGAACTTCTGAGGAAGATTCTCATTGCAGTAATTCTAATGCCAACGTAAAGAAAGATA
ATGTTGATGCTGATACTAAGACCAATGCAAATGCTGGTTCCACTACCAAAACTGAGAAACA
GACTGCAGGAGTCCTTGTTGAACATAATGTGAATGATCTGCTATTCTCTCCTGATCAAGCTG
GTCTAAAAGATGAAACAGCACTGGCCACTACTTCTCCAACACCAAGAAATCAGCATTTTAA
AAGCTTTGGAGCTAATCAAGGTGTTGCACCCGAGCATTCACCTGCAAGTGCATGCTCTCCA
GCAATTTCCAAAAAGAACAATGGAAGTAATTCTAGTGCAATTAAGACAGTAAAACCTATTC
CTTCATTGACACCGCCAGGAAATACGCTTCTTAATGTTGGGAGTGATGTTGGCTTCGAAAAC
TATAACCTATTTGGTGAAACTCCATTCAAAAGAAGTATAGAATCCCCTTCAGCGTGGAAGTC
CCCTTCAGCTTGGAAGTCTCCTTGGTTTATGAACTCTTTCGTACCTGGTCCAAGGATTGACA
CTGAAATAACAATTGAGGATATGGGTTATTTTATGAGCCCAGGAGACAGAAGCTATGATGCC
ATTGGACTGATGAAACAGTTAAGTGAGCACACTGCTGCTGCATATGCTGATGCCATGGAGG
TTTTGGGAAATGACACTTCTGGAAAATCAGTGAACGAAGGAAACACTAAAACTTTAAGTTT
GGACGAAGAAAGCAATAATGTCACCCATAATGAGCCGGAGAGCGACCATTTGGCTTCAAAT

ATTTTGATTGAGCGGCGCACACTTGACTTTAGCGAATGTGGAACACCTGGGAAAGGAATAG
ACAAGGGGAAATCCTCAACTGCCATAGACTTCTCAAGCCCCTCCTCATATCTATTGAAAGGA
TGCAGAAGGAAAGTCTTTATATTTCTCAGCGTTACCTAGTTTGTGAAGAGGTTCTTGAATA
TGTTAACCTTGGAACTGGTTTGAAAGGAATTTTGAGTCTTAGGGTATTTGAGGTATGA

>MiMYB4

ATGACAGCCAGCTCCGATATTTATTCCACTACTACTACTTGCCTCAATAACTCATCAATGGAG
GTGTTTTCCCACATGGGTTCTCTCCATGCTGCTCCAACTACTACAAATTCTGAGAAAAATAG
TCTTAGTAACCTAGAAGATTCTGATGGGTTTGAAGAAAACACTTATAACTTGAGTGAGGATC
ATCATGTTGTGAAAGAAATGCATAGTGGTGGGCAGTCAAAGAGTAGTTCCAGAGGGCATTG
GAGACCTGCAGAGGACACTAAACTCAAGGAGCTTGTTGCATTTTATGGTCCTCAAAACTGG
AACCTTATAGCTAAGAAGTTGCAAGGGAGATCTGGTAAAAGTTGCAGATTGAGGTGGTTTA
ATCAGTTGGATCCGAAGATCGACAAAAGAGCTTTCAGTGAAGATGAGGAAGAGAGACTAA
TGGCGGCTCATAGAGTTTATGGCAACAAATGGGCTATGATTTCGAGGCTTTTCCCAGGTAGA
ACTGATAATGCAGTTAAAAATCACTGGCATGTTGTAATGGCGAGGAAGTTTAGAGAACAAT
CCAGCGCTTATAGGAGGAGAAAACTGAACCAAGCTGCTGTTCATAATACTGACCAAAACTG
TGCTCAAAATATGAGCAATGTCAGCTTCATAAATCCAGCAAAAAGTCTTGACTCATTTGCAC
CTCCCATTGCTGCTGGTGGTGGAGATTTGTTTCTTGGATCTACAAGCAGCTGCAATAACTTT
AACTTTCTCCCTGTTCATGATGATCAAAATCAAAGACCTAATTTTGCCGCTTGTGGAAATCA
GAAACCTCCTTTTGGTTACAATGGAGATTACATGAGTTTAGGCCTGGAACAAGACAGCTCA
AGCTTCAATATTTTCAAGCCTTATACCGTAGACAATAATTTTCCAATGCAACAATCAAACTAC
CAGCACCATTCTGCAGCTTTCTCAGAGACTATGGCATCGGCATCGGCATCGGCGGCATCTCC
AGTCTCAATCACTGAGCCATCATCTTCATCACTGTCACTTGCAGATACCACAGCAACCAGCC
ATTTTGAGACTAACATTTCACCACCATTTATAGATTTTCTTGGCATTGGAGCCGCTTCATGA

>MiMYB5

ATGGTTCATAATTCGTCGCTGTTTTCCTCATGGACTCGGGAGGAAGACAAGCTCTTCGAGAA
CGCTCTCGTGTTGTTTCCCGAGGAAACGCCGGAGCGATGGGAAAAGATCGCGAGTAAAGT
CCCTGGAAAGAGTTCCGATGATGTGCAAAAGCATTACGAGGATTTGGTTCGTGATTTGAGA
GAGATCGACTCCGGGATGGTTGAATTGCCGAATTACGAGGACGAAATGGACTCACCGCGTT
GGAGCAGTGAGTCTGGAACGAATCAGATGTGGCCTGGGGGTAAGTCTAAAGAAAGGGAGA
CCGAGAGAAGGAAGGGCGTTCCATGGACTCCTGAAGAGCATAGGTTGTTTTTATATGGTCT
AGACAAATTCGGGAAAGGAGATTGGAGGAGCATATCGAGAAACGCCGTCGTAACACGAAC
ACCAACTCAAGTGGCAAGCCATGCTCAAAAGTATTTTCTTCGTTTAAACTCGATGAAGAAA
GAGAAAAAGAGAGCTAGCATTCATGACATTACCGGCACCGTTGATTCCAAGTCATTGGGTC
AGTCCAACGATGAGAAATGGGCCACTGAACCTAGAAACCAACAATACAACAAAAATCATG
GCACCTCAATTGGCTATCAAGGTTTCGGATATCCAATGTAA

>MiMYB6

ATGAAGTGGGAAACAGGAGTAATCTCCCCTCCAACATATCTTTCAAGCAACAATCGGGCTC
TTGAAGATAGCAAGACCACAAAATGGACAGCCCAAGAGAACAAGATGTTTGAGAATGCTT
TAGCAATATATGATAAGGATACTCCTGATCGATGGCACAAAGTTGCGGCGATGATCCCCGGG
AAGACAGTAGTAGATGTAATTAAGCAGTATAAAGAATTGGAAGATGATGTGAGCAATATAGA
AGCAGGGCTGATTCCAATTCCAGGGTATAATAATACTACTAACTCTCAATTCACATTGGAGTG
GGTTAACAATCATAATAGCTATGATGGGTTCAAACAATCATATGGAATTGGTGGAAAGAGGT
CCTCTTCTGTTCGCCCTGCCGAGCAAGAAAGGAAGAAAGGAGTTCCATGGACTGAAGAGG
AGCATAAGCTGTTTCTCTTGGGATTGAAAAAGCATGGCAAAGGAGATTGGCGAAACATCTC
TCGCAATTTTGTTACCAGTAGAACACCGACCCAGGTGGCTAGTCATGCTCAAAAGTACTTTA
TCCGGCAGCTCTCCGGGGGAAAAGATAAGAGGAGAGCTAGCATTCACGACATAACAACCG
TAAACCTCAATGACACAAGAACTCCTCTGGACAATCAAAGCCCTCCTTCACCGGATCAGTC
TGCAGGACTTTCTCAGCAACCCAGCTCAGGCGCCTTGTCTAGTTCACAATTTCAATGGCATC
AGCCAAATAGTGGATCAGCCATGTCCTTCCTTTCAACACAAGGAAATATGTTAATGGCATCG
CCTTATGCGATGGCCTCATATGGGCTAAAAATGCCAGGACAGAATCTGCAAAGAGGTGCAG
TGCATGAGCCTTACTTTGGACTTCAAAATCTGGCTTTTCAAATGCAATACCCTCATGGATAA

>MiMYB7

ATGAACAGTGACTTCATTACAGGACTCAAATTCCTTATCGACAACCGAAATACAGGACTTAG
GGTTTCCGTTCTTTGCCGAAATCTCCTGAGAGAGGTCACTACTACTACCCTCACCTTGGAGA
TGGAATTCGAGATATTAGGTCTTGAAGGAGCGTTTAGTGAGACGAAGGAGATGACAGAATT
GCAAGTAGACGAGTGTTGCCTTGAGAACAAACGATTAACAATCGCTTCAAGCTCTTCTGTA
TCTGAAGGTAGTGGCAGTGCTTTTCTAAAGTCCCCTGGAGTATCTAGCCCTGCAACTACATC
ACGGACTCATAGGAGGGCCACAGGACCGATTAGGCGAGCTAAAGGAGGTTGGACACTAGA
GGAGGATGAGACATTAAGAAATACTGTAGCAACTTTTAAAGGGAAGAGTTGGAAGAAAATT
GCTGAGTATTTTCCTGATAGATCAGAAGTACAATGTCTGCATCGATGGCAGAAAGTTCTCAA
TCCAGACCTTGTTAAAGGACCTTGGACTCAGGAGGAGGATGATAAAATAATTGAACTAGTG
TCAAGATATGGGCCCACAAAATGGTCTGTCATTGCAAAGTCTTTACCTGGTCGTATAGGAAA
ACAATGTCGAGAGAGGTGGCACAATCATTTAAATCCTGATATAAAGAAGGATGCCTGGACT
CTAGAGGAGGAATTAGCACTAATGAATGCCCACAGGAGATATGGGAACAAATGGGCTGAAA
TTGCCAAGGTTTTACCTGGAAGGGACTCAGATTCAACTGCCCAAACTTCATCAGAAAGTAG
AGACTTAGGCAAGCTGAATGAAGATGGCAAGGATCAAGTGGAGTCCTCAACTCTAGTTCTA
GATATGGCTGCTTCATCAAGTGTTCGTCCAAATGATTCTCTCAATTCTGATTTTGTTGAATGT
AAGCCACAGTCAGCTAAGATAGATCTTAGCTGCTCGGAATCTATGCCAAAGCTTGAGAATTG
TGCAATCAATTCTGAACTTGTTGACAACCAAGTAATTGGGTCACAACAGCAATTTGGAACT
CCAACCTATGGTTCTTTGTGCTATGTTCCATTATATTCAGATCCTTTAAACAAATGTCTTCTGC
AGCATGAGTGCAACTCTACTCCCATTACTTCACCGATTAGCTACTTCAGTCCACCTTGTGTG
AAGGGCAGTGGTTTAAGTGCTAGAAGTCCTGAATCTATATTGAAAATTGCTTCTGAGACTTT
CCCGTATACTCCTCCTATTTTAAGAAAGAGAAAGTCAGAAGGTCAAGCTTCATCTGATACTA
AAATAATTGGAAAAGTAGATGGGGAGACAACAGATACAAGTACTCCTATTGAGTCTACTGG
AAAGGCTTTCAATGCATCTCCTCTATATCGGTTAAGATCCAAACGCACTGCTGTTTTCAAGT
CTGTGGAAAGACAACTTGAATTCACTTTCAACAAGGGGAAGCATCATGATAATACCAAATC
TAGGGAATTGTCTGTGAATGCAAGTTGTCCTATTACCGAAGATTTGCACACAACAAAACTC
AGGTGA

>MiMYB8

ATGAAGGAGAGACAGCGTTGGAAAGCTGAAGAGGATGCTTTATTATGTGCTTATGTAAAGC
AATATGGCCCAAGGGAGTGGAGCCTTGTATCACAGCGCATGAACACACCCCTAAACAGGGA
CGCAAAATCGTGCTTAGAAAGGTGGAAGAACTACCTCAAACCAGGCATCAAGAAGGGATC
TCTTACTGAAGAGGAGCAGCACCTTGTCATCCGTCTTCAGGCCAAACACGGCAACAAGTG
GAAGAAAATTGCAGCTGAAGTCCCTGGCCGCACTGCTAAGAGACTTGGTAAGTGGTGGGA
AGTGTTCAAAGAGAAGCAGCAGAGGGAACAGAAGGATAACAGCACTCCAGTTAATCCAAT
TGAAGAGCGCAAGTACGATCAGATTCTAGAGACTTTTGCGGAGAAGCTAGTGAAAGGCGG
TGCATTTGTCATGGCTGCTTCAAATGCAGGGTTTCTTCACACTGACTCTCCTACCCCTTCAA
CTTTGCTACCCCCCCTGGCTTTCAAATTCCAATGGATCCTCCACTGTCAGGCCACCATCACCT
TCTATAACTCTGTCTCTGTCTCCCTCAACAGTGGCAACTACTCCTACAATCCCATGGCTGCA
GCCTGAGAGGGGGCCAGATAGTGCCTCTCTAGTTCTCGGAAATTTGCCACCTCATGGCTCA
GTTCCCTCTTATGGAGAGAACCTCTTCATAACTGAGCTGGTGGAATGCTGCAGAGAGTTGG
AAGAAGGGCATCATGCTTGGGCAGCACATAAGAAGGAAGCAACATGGAGGTTGAGAAGGC
TGGAGTTGCAGCTGGAATCAGAGAAGGCATGTCGAAGGAGGGAGAAAATGGAAGAGATAG
AGGCGAAGGTGAAAGCTATTCGAGAAGAGCAGAGAGCTACTTTGGATAGAATTGAAGCTG
AATACAGAGAACAAATTGCTGGACTGAGGAGGGAAGCAGAAGCCAAGGAGCAGAAATTG
GCTGAGCAATGGGCTGCCAAGCACCTGCATCTTGCCAAGTTTCTTGAGCAGATGGGGTGCC
GGCCCCGGCTAGCGGAGCCCAATGGCCGGTTGGCCAATGTCAAATCTAGTCTTAAATATGCA
TTTTGTGATTAG

>MiMYB9

ATGGAAACACAAAATATAACGGACATCGAAGTCGACGGCGACGACACCGAAGACGGTGGA
GAAGTTGAAGCTGATGGCGGCGGAAGCGGCGAAGATTCTGCTGTAGTTGTCGGAGAGGGA
AGCAAAAGGGGCAACAAAGACCGAGTGAAGGGGCCTTGGTCGCCCGAAGAGGACGCAAT
ATTAAGCAACCTTGTGAGCAAGTTTGGCGCTAGGAATTGGAGCTTGATCGCCCGAGGAATA

TCCGGTCGCTCCGGCAAGTCTTGTCGGCTCAGGTGGTGTAATCAGCTCGATCCAGCTGTTA
AACGGAAGCCATTTACTGAGAAATTGAAGTTGGGTCTTGGTAGATATAGACATACGCCCTCT
AGCATTAGACAGACTGACAGAAGCAAATTCATTTCAGATGAGGAAGATAAAATTATAATTTC
AGCTCATGCTATCCATGGAAACAAATGGGCTGTCATTGCTAGGCTTCTACCTGGGCGGACAG
ATAATGCTATTAAGAACCACTGGAATTCCACTCTAAGGCGTCGGGCCATGGAACTTGACAG
GGTAAAGAAGTTCGAATCTGGAAATATCATTGAGGATACCAGCATGGATAGGACCAAAGCA
TCATCTGAGGAAACTCTATCTTGTGGTGATGTAAATTCATTTAAATCCTTAGAGGGAAAAGA
TGTATGTTCTTTGGAAATTTTGGATAATCAATGTGAAGACAACACCATAACAGAGGTTCCAT
ACAATCCTGATCATGAATTAAAAGAACAACCTACTCTTTTCCGTCCGCAGGCATGTGTAAGT
GCTTTCAATGTTTATAAAGCTGTGGATGGCCCTGAGATTGCTTCACCAAATTCAAGGCAAAC
CCCAATGCAAAGACCTCTAGTTCAAGCATCATTGCCTGATGCAGGGATCTGCAAATTGATTG
AAGGAGTCTATGGTGAGAGGATGGTGCCTCACATGTGTGGCTTCGGCTGTTCTGGGACACA
ATGTGGTGGTAATGGTCAGAACTCTTTGTTGGGACCTGAATTTTTGGAATTCTCAGAGCCTC
CAAGCTTTCCAAGTTTTGAATTGGCTGCCATAGCCACAGAAATAAGCAACCTTGCTTGGCT
GAAAAGTGGCTTGGAGAATAGCAATATGAGACTGATGGATGATGCAGCAGATAGGATAAGA
ACAAATGGATCTCAGGTGCAAATGGGGCATTTGGAAGAAAGAAAGAGCAAGTTAACAGGG
CAATAA

>MiMYB10

ATGAATCGAGGAGTAGAAGTTCTGTCTCCGGCCTCATATCTACAGAATTCAAACTGGTTGTT
TCAAGAAAGCAAAGGAACAAGATGGACTCGACAAGAGAACAAGCTGTTTGAGAATGCTTT
GGCTTTGTATGATAAAGATACTCCTGATCGATGGATTAAGGTAGCAGCAATGATTCCAGGCA
AGACAGTCGGTGATGTCATCAAACAATACAAGGAATTGGAAGAGGATGTTAGTGATATTGA
AGCTGGTTTAATTCCAATTCCTGTCTATTGTAACGATTCTTTCACTTTGGAGTGGGTTAATTG
TGATCAAGAATTTGATGGATTGAGGCAATTGTATAGTCCTGGTGGGAAGAGAGGCACCACA
ACTCGGCCTTCTGATCAAGAGAGAAAGAAAGGGATTCCATGGACAGAAGAAGAACACAGG
CAATTTCTAATGGGTCTTAAAAGGTTTGGAAAAGGGGACTGGAGAAATATCTCCCGCAATTT
TGTGACCACTAGAACACCAACTCAGGTTGCAAGCCATGCTCAAAAGTACTTCAATCGGCAG
CTTACTGGGGGGAAGGATAAGAGGAGATCTAGCATTCATGACATTACTATTGTCAGTTTGGA
AGTCACAGGATATTCTTCGCCAGAAAATAGTAAACCTTCATCTGCAAATTCTTCTACAGCTG
TCATTCAGCTGCAACAGCAACCAAAATTGACTAGCATAAGAAATGAGCGATTTGATTGGAA
ACCACCAAATGAAGGAGCAGCCATGGTTTTCAACCCAACAAATAGCAACGTTTTCACACCC
CCTTTTTGTGGGATATCTTCATATGCAGCTAAGTTGCAGGAGCAAAATCTGCTTGGTGGAAC
TCTTCATGGATCTCAGTGTGACATGTACAGCACATTTTATCCGATGCAATCAATGCAACGTCA
GTAA

>MiMYB11

ATGGTAATCCATGCTAGTTATCGTTTAATTTCCTACTCAAAAGAGATTGTAGATGGACAGCAG
ATTTTTGTTTCTTCAAATTGTCTTCCTGTAAAGTCTTCAAAATATGAACCTGCTGGTCATTCT
TTTCATGCTGCTGCACTTAAACTCCTTGGGTGTGAGGAATATACTGATTCCGATGATCAGAA
AGTTTCCAATGATAAGGAACATACATCTTTGCCACAATATGAGTCTTATAGTAGCAAAGGTA
AAAAGAAGTCTGGTACAGGAAGCAATCAGCAAGATCATTATGCACTTTTGGGATTGAGTCA
TTTGAGATACCTTGCCACTGAGGATCAGATAAGAAAAGCTTACCGTGAGACTGCCTTGAAA
TACCATCCTGACAAGCAGGCTGCACTTCTTCTTGCCGAGGAAACTGAAGCTGCAAAACAG
GCAAGGAAGGATGACATAGAAAGTCACTTTAAGGCTGTCCAAGAAGCATATGAGGTCTTGA
TTGACCCTGTGAAGAGAAGGATATATGATTCCACAGATGAGTTTGATGATGAAATCCCATCT
GACTGTACCCCACAAGATTTTTTCAAAGTCTTTGGTCCAGCTTTTATGAGGAATGGAAGGTG
CTTTAAAAGCTGGAGAGAGTTCCCACATGCAGACGAGTTTGATCTTGAACAGGCCGAGTCT
CGTGATCATAAGAGATGGATGGAGAGGCAGAATGCTAAACTTTCAGAAAAAGCTAGGAGG
GAAGAACATTCACGGGTGCGTTCTCTTGTTGACAATGCATACAAACGAGACCCTAGAATTC
TGAAGAGAAAGGAGGAGGAGAAAGTTGAGAAGCAAAGGAAAAAGGAAGCTAAATTTCTG
GCAAAGAAGTTGCAGGAAGAAGAAGCTGCTAGGGCTGCTGAAGAAGAGAAACGCCGAAA
AGAGGAGGAGGAAAAACTAGCAGCCGAAGCTGCATTACAACAGAAGAAGCTGAAGGAAA
AAGAGAAGAAGTTCTTACGCAGAGAGCGGACTCGACTTAGAACACTTTCAGCATCCATTGT
ATCTCAGCATTTGCTTGATCTATCTGAGGAGGTTGTGGAAAGTCTGTGTATGTCCCTTGATAC
TGAGAAGCTGAGAAGTTTGTGTGATAAATTGGAAAACAAGGAAGGCCTGGAACAAGCCAA

AGTTATAAGGAATGCACTAGGGTGTGTTCATGAATGTGAGGGAAAGAACCAAGATGAAAA
AAATAATTTACAGCAGAATGGTTCTGCAGAGGCTAATGGAATTATCCATTTAAAAAGCTTTG
AGAAGAAGGAGAAGCCTTGGGGAAAGGAAGAGATTGAGCTATTAAGAAAAGGAATCCAA
AAGTATCCCAAAGGAACATCTAGAAGGTGGGAGGTTATTTCAGAGTACATTGGTACAGGAA
GAATTGTGGAAGAAATTCTGAAGGCAACCAAAACAGTTCTCCTCCAGAAGCCTGATGGTG
CAAAAGCTTTTGATTTTTTTCTTGAGAAGAGGAAACCTGCACAGTCCATTGCTTCTCCCCTT
ACAACTAGGGAAGATGTGGAAGGGTTATCGATCCCCCAGGGGCCTCAGGAAACAGCTGCA
AAGGTGGATATCCCAGAAGGGTCTACAAGTTCAAAGAGTCCTGTGGTGTCTGCTTTAGATG
GGGTTTCTTCGAGTTCTGATCAAGATGTGTGGTCTGCTGTACAAGAAAGAGCACTGGTTCA
GGCTCTAAAAACCTTCCCCAAGGAAACCAGTCAGCGCTGGGAGAGAGTTGCAGCTGCTGT
TCCTGGAAAGACTGTAAATCAGTGCAAGAAAAAGTTTGCACTACTGAAGGAGAACTTTAG
AAACAAGAAAAGTTCAGTTTAG

>MiMYB12

ATGTTGATCGATGACAAAAGTAATGATCAGCAACAACAACTTGAAAATGGATCCTCGGGAA
TGAATGTAGAGGGAGGAAGAGATGGAGGAGTTCAGTTGAAGAAGGGGCCGTGGACGGCG
GCGGAGGATAGTATTTTAACGGAGTATGTAAGGAGACACGGCGAGGGTAACTGGAATGCCG
TGCAGAGGAACAGCGGATTGGCCCGTTGTGGCAAAAGTTGCAGGCTCCGTTGGGCTAACC
ATTTGAGGCCAAATTTGAAGAAAGGCGCTTTCTCTCCTGAAGAAGAAAGACTTGTTGTCGA
ATTGCACGCTCAGCTTGGTAACAAATGGGCTCGCATGGCTGCTCAGTGCTTCAGCCGCAGC
CGCACCCAAATTCCCACCTCTCTCTCCCCAACCCCTCCACAACTCTCCCCTACCATCCAGTC
TCCAACTTTCCCAACTCTTTCTCTCTTTAACTCCACAAATGGCTATCAAACCAACCATCCAA
ACTCAGCTACAAATATGAGCAACGCCTTTATTATCTCTAGAGCTCCTCCTATTCTCCAAAACC
CAATTCGTTTCAAACGTTTTCATCCTTCTAACAACAGCCAAAACATGGAGAATAGTCATAAT
ACTATGAATTTGAGTTATCAAAATAAGAACTTTTCGCCTCCTTTTAGTCCTCAGTTCTTGAGA
AACCCTTCATCTCCTATACTAACTCCCCAGAGTACAAACAGTAATATTTTCTCAAATAATTCT
ACGTTGTTGTCACAGCCTTCGTTTTCATCTCTTCCCGTTAACTTCAATGCTCAAAACTCATCA
ATGATTCCAAGTCTGGTGGAGAAGGATGGGTTTTTGAGCAGTGATTCAGTTGGGTTTAGTTT
GAATCCGGAGCTCCCTTCAATTCAAGCGTTGTCTCAGCAAAATGGAAATGGTTTTGATAATG
AGAAGTTGAGTGTGTCGAGCAGTGGAAGTGGGTTGTTGGAGGATCTTTTAGAGGAGGCGC
AGGCCATGGCTAGCAATTATGGCAATAGTTCAAAGCAGCTAAGCGGTGTGGATTCACTTGA
AGATCAGAACAAGCCTGAGTTTGGTGGGTTCAGTCAATGGAGTGATTCAAACTCTGTGAAT
ATATCATCAGGAATGAAGCCGAAGGAAGAGAATGAGCAAGGCCAAATGAGTACAATGCATG
AAGACTTATCAAAATACCTGAATGAGATTCCCTCCACGCAAGTTCCAGAGTGGTATAATGAC
AGTGGCGAAGCCTCAAATGGCCAATCTCCAGTAGTAACCGACGATAATTTTGGACTTGACA
TGCAGCAAATAGCCTCATTATTTCGAGTTGACTCTAACACAGGCCAAGTCAGTGACAATCA
GGGCTCCTGCCCTTTTGATAATTTGCTAGGAATCTGCTAA

>MiMYB13

ATGCCATTTCACGGTGAAGACGACGGAGAAGAATTTTCCGGCAGCGAGAACGACGACGGT
TTTGATGAGGAGGAAAGGGAAGCCATAAGGAGAGCCTGTATGATCTCAGGCACCGACCCC
AACGACCTCGATAACACCAACAAACTTCAGCTGACCGTCGCTGCCGATTCCTCCGGCGGCG
TCAGTGCTGATTACTGGAGTTCCGATAGCGAAGACGATCGTGAATTGGTCCGGAAAATTCA
GAACCGGTTGGCGTTGTCGGATGACTCGTGCCAGCCATTGTGTGCCCTTCCACCGGTTCTTT
TGGATGATGATGAAGAGGATGATTTCGAGACTCTTCGCGCTATTCAGCTGCGCTTTTCAGCT
TATAATTCAGCTGATACTACAAAAGGCAGCTGGAAGGATTCTTTACAGACTCCAAATCAGGT
TTTTGCATCTAGTGTAGCATCAAAGAATGAGACTTCCAATGGTTTGTTTGTAAATAGAATTAA
TTCTTGTGAAGGGTTTCCAGATTCTGAAGAAGCTTGTAACAACCACCATTTATCGAAGGTCA
ATGTGGAGACACTGCCTTCTGGTTCCATTGAGTGGCAACAGTCAGAAAAATATAATTTGTCT
ATGTTGTCACAAAATAATTCTAACTTCCCAAAATCTGCTCAGATGTTTATTGATGCCATTAAG
AAGAATAGGTCGTATCAGAATTTTATCAGAAGTAAGTTGACTCAGATTGAATCAAGAATAGA
GGAGAACAAGAAATTGAAAGAACGTGTAAAAATTCTGAAAGACTTTCAGGTTTCTTGCAG
ACGAATAACTGGTCGAGCATTATCACAGAAGAAGGATCCCCGTGTCCAATTAATATCCTCAC
AGAAGTTAAGAAATGGCAATGACTCAGAGGTTTTTGATAAGAAATCCTCTGCCATTCACTAT
GGCCCATCAGAAAATTCCCATGTTTCTAACTATAGAATGGCCTTGTCAAGATATCCACTTTCA
TTGCATCGGAAAAAAATGGTCAAAGACAGAAAAGGAAAATCTTGGAAAGGGAATAAGACAG

CAATTTCAAGAAATGTTGCTGCAAGTGTCAATGGATAGACTAAGTGGTTCAGAGGGATCTT
CTATGGATACAAATGGTTTGGATAACATTTTTGCATCTATCAAGGATCTTGATGTCACCCCAG
AATTGATCAGGGAATTTCTACCTAAAGTTAACTGGAATCAGCTAGCTTCCATGTATGTTAAG
GGCCGTTCTGGTGGAGAATGTGAAGCACAGTGGTTGAATTTTGAAGACCCCTGTATTAATC
ACAATCCATGGACTATCGAAGAGGATAAGAAGCTTTTGCTTATCATTCAAGAAAAGGGGAT
CAGTAATTGGTTTGATATTTCAGTCTCATTAGGGACTAACAGGATTCCATTTCAATGTTTGGC
TCGTTATCAAAGGAGTTTGAATGCATCCATATTGAAAAGGGAATGGATTGAGGATGAGGATG
AACAACTGTGCATGGCTGTAGAAGCTTTTGGTGAGAGTAACTGGCAGTCTGTAGCTTCAAC
TTTAAAAGGCCGGACTGGTCCTCAATGTTCTAATAGATGGAAGAAAACTCTTCACCCCATG
AGGCAAAGAGTTGGGAAATGGACACCAGATGAAGATAAACGCCTGACAGTAGCTGCAATT
CTTTTTGGGCCCAAGAACTGGAAGAAAATTGCTCAATTTGTGCCAGGCCGAACTCAAGTGC
AGTGTAGAGAAAGATGGGTCAATTCTTTGGATCCTTCTGTGAATAGGGGTGAGTGGACTGA
AGAAGAGGATTTGATGTTGGAAGATGCAATCAAAAAACATGGATTTAGCTGGTCCAAGGTT
GCTGCTGCATTGCCTGCACGGACTGATAACCAGTGCTGGAGGAGATGGAAGTATCTGCATC
CAGATGAAGTACATGTTCTTCAAGCACGTAGAAAGATGCAAAAGGCTGCTCTAATGAGCAA
CTTTGTAGATCGGGAGCGGGAGCGCCCTGCCCTTGGTCCAAATGATTTTATGCCTTTAGCAT
TGACAAATCCAGCATCTGAACATGGCGACGTAATTGCATCCCAGAAACATGAAAGAAAATC
AAGAAGAAAGGCAGAATCTGGGAACAAGGAAGATGCTGCTCCTTGCGAAAATCAGAAAA
CAAGAAAATCCCAGAGAACTAGAAAAAAGACACAAATTTGTTCTGAGGTTTTGGAGATAA
CTTACGGTGATGACACTGAAACCTCTAATCAACGTGGTGTAATTCTTAAGAAGAAAATTGTG
AAGCTTCATTCAGGGAAGAAAAAAGTTAATAGTGAGCCCAATACTGTGAAGAAAGTATCTA
AAAGATGTTCAAAGATGCCATCATGTGCAGAATTGGATGAGATGAGTATTCTTCTGCCACCC
CCTGAAAGTGTGGAAGCAGAGATAACTTTCACTGACAGTAGCAATACTCCTTGTGGCAACA
CCAATGTGTCAAATTGTGACAGTATAGATCCTATTTGGAATTCTGCAGGCAATTTATGCTCTA
TATCTTGTCAAGAACAAGATGCACCATACTGTTCTGAAGTGGGCATTTTGCGGGGAACTATT
GATGATATTGAATTATTGCAGAATCACTCAGATTATCTTGATTCGACTTTGTCAACAACAATC
AACAGTGAAGATGGCGACTTACTGGGTGGAATTGATGCTTCTACAAAGAAGAGGCCTTCTA
AATTGCTACCAAAAAAGAAATCAGGCATGAAGTCAGGTAAGGAGAGAACTGAAGGTGAAA
ACATCTCTTTGGAGAGTGTTTGTGAGACTGTAAAGCGAAATAACAAAAGGAAAATATGTAA
TGAACCATCAGGGAAACGCCAGGATGTTGCAAGCATTTCTTGTGAGCAAGCTGGGTCTAAA
ACACTTTTAGAAACATCAGATGGAGAAGACATAACACTTGAGTGCTTTCTTTGTAATAAGTC
AAAGAAGAGAAAGCTTAAAGCTGGTTCTTCCTCTGAGTCATTGCTGCTTTCCAAACCTGTA
GATCAGCACATCAGTGAAGGGAAAATATGTAATGAACCATCAGGGGAACTTCAGGATGTGG
CAAGCATTTCTTGTGAGCAAGATGGGTCTAAACAATGTTTAGAAACATCAGTCGGAGAAGA
CATAACACTTGCTTGCTTTCTTCGTAATAAGTTAAAGAAGAGGAAGCTTAAAGCCGGTTCTT
CCTCCAAGTCATTGGTTTCCAAATTGGTAAATCAACACATCAATGAAGATCATATGCCAGCC
CACTTGCAAAATGGTGAAGCTGAGACCACCAATGGGAGCTCTGATGAACCAGTGACAAAA
CTGAAGAGCATTAATTTGGAAGGTGATGGTGAAGTTGGTGAAATTATTTGA

>MiMYB14

ATGTGCACCAGAGGACATTGGAGGCCGGCAGAAGATGAAAAACTCAGAGAGTTGGTGGAA
CGTTACGGCCCTCATAACTGGAACGCCATAGCTGAAAAACTTCAAGGCAGATCAGGGAAA
AGTTGTAGGTTAAGATGGTTTAATCAACTGGATCCCAGAATCAACAGAAGCCCTTTCACAG
AAGAAGAAGAGGAGCGGCTTTTAGCATCTCATCGGATCCATGGAAATCGATGGGCAGTGAT
CGCCAGATTGTTCCCCGGACGAACTGATAATGCAGTGAAGAATCATTGGCATGTCATCATGG
CTCGAAGATGCAGAGAAAGATCAAGACTTTACGCCAGAAGAGCGGCTCAAAATCTCCTGA
ATAATGAGCAGAAATCGCCTTCAAATCCAGAAATCTTCTCCGCCTATTGCCAGAAAAATCTC
TACAGTTTTGCTCAGAATCATAACCATCTTCTTCATCAGTGTTATCCGGTGCAGGTTTTCAAA
GAAGTGGAAGCCGAAAATACAAGCCATTGTATCACCATGAATCAAGATAACAAGAACCAGC
GGGTGGAGTTTTACGATTTTCTTCAAGTGAATACAGATTCAACCAAGAGTGAGGTGATAGAT
AATGCGCGAAGGGACGATGAGGAAGTGGATCAAGAAGCGACGGAGCAGCAGAAAACAAG
GGCACAATTTATCGACTTCTTATCAGTAGGAGGAGATTAA

>MiMYB15

ATGTCCAATAATTCAGCCTCTTCTTGGAGTAGAGATGAAGAAAAAGCTTTTGAAAACGCCA
TTGCAATGCATTGGAATAGTAGTAGTAGCACTGCTACTACTACAAGTGAAGTAGGCGACTCA
TCATCATTAGAAGAGCAATGGCAAAAGATTGCTTCAATGGTTCCAAGTAAAAGTATAGAAG
AATTAAAGCAACATTATCAGTTGTTAATGGAGGATTTGCGGGAAATAGAGGCTGGACACGT

GCCGCTACCAAATTATGTTGGAGAATTAGAAGGTGCATCATTGGCTACATCAAGTAAAGATT
TTCTTGGCTTTTCAGGCTCAGTAAGTGGCGAGAAGAGGTCAAATTGTGCCTATGGAAGTGG
GTTCACAGGGCTGAGCCATGAGTCAAGTGGGCATGGAGGCAAAGGAGGCTCAACGGCTGA
CCAAGAAAGGAGAAAAGGAATTCCATGGACAGAAGAAGAGCATAGGTTATTTCTACTGGG
TTTGGACAAATTTGGGAAAGGGGATTGGAGAAGTATTTCAAGAAACTTTGTTATATCAAGA
ACCCCAACACAAGTTGCTAGCCATGCGCAGAAGTATTTCATACGGTTGAACTCAATGAATAG
AGACAGGAGGAGGTCGAGCATACACGATATAACAAGTGTGAACAATGGAGAGGTGTCATC
TCATCTTCATCATCATCAAACTCCAGGGCAACAGGGGGACAGAAACCCAGGAGGGGGTCC
AGCAATGGGAGCATCGGTGAAGCACAGGGGGGGTCAGCCACATTTGCTTGGCTTGGGAAT
GTATGGAGCTCCTGTTGGGCACCCAATTGCAGCTCCTGGGCATATGGCTGTTGGGACTCCTG
TTATGCTTCCTCCACCAGGCCATCATCCTCATCCTCCTCATGTTGTTCCGGTGACTTATCCAA
TGGCAGCGCCACCGGCAATGCACCAATAA

>MiMYB16

ATGGAGTTCGATTCAAGTTTCAGAGAGGATTACCCTTTTCTTTCTAGCCTTCTTGCTGATAAT
CCTCTCAGTAATCCTGAGTTTAAAAATGGGTTTTCTTCTCTTATTGATAACACATCATCGCCT
TCTTTGAACAGGGGTTTATTTGAGCTTGATCATGACTCGCTGATGAACCCTGAAAGTTTTAA
CCAACTTAACATCGAGGGTTCGTCGAAAAATCCCTTTAGGGTTTCGAGGCCTAGTTTCGATC
CCTTCGAGGTTAATACAAATGTTTTTTCAGCTGATCATGTTGTTGACTGCAATCCTTTGATGG
CAAATGGACTCTTGCATGACTCAGAAAGAAGCGCGTTTTGGGCTCAGTCTGTGCCGGAAGC
TCAGATTTATGAGCCTACCAAATTTCAAGAGTTTGGATCAGCCGCTGCAAGGCTTCCAGATG
AAGTCTCGTGCATTACTGCCGATCAAAACGCTACGTATCATCAGAAAGTTGAGCAGAAGAG
GCACAAAAAGATACAAACAAGAAGAAGCGGCAAAGCTCCGAAGAAGCACAGCCTAATCA
AAGGCCAATGGACAGCTCAGGAGGACAAGACGCTGGTGCGGCTTGTAGGTCAATATGGTA
CAAAGAAATGGGCACAAATTTCTAAGATGATGAATGGGAGAGTGGGGAAACAGTGTAGAG
AAAGATGGTACAACCATTTGAGGCCTGACATCAAGAAAGAAGCCTGGAGTGAAAATGAAG
ACATTATACTGATTGAATCACACAAGCAATTAGGGAACAGGTGGGCGGAGATAGCCCGGAG
ACTGCCAGGTCGAACTGAGAATACTATAAAGAATCATTGGAATGCTACCAAAAGAAGGCAG
CAATCAAAGAGAAAGAACAAAGACTCGACTCCCAAAAGCACCCTCTTGCAGAGCTACATC
AACACTGTCACCTATTCAACTTCATTTATCAAAAAGGGCAAAAAACCCATGAAGAAAACCA
ACGTACAACAACTGGTTAGCAACGCCATCATGCCCACTCCACAAATTCATCAAATGGAGAC
CTCAGATTTCACAGCTGATTGGCAAGTCCCATCAGCACCAGCAGCATATGATCAAAATGAA
GCCATGGGGTACTCGTTTGATGCTAGCATGTTGTGCGAGGGGTTCAACTTTGGGTCGATGCT
GGATGAGGCGACTTGTGGGTCGATGGTTGATGAGAGCAACAATGAGTTTGAGCTGCCATTG
GAAATTGATAGCTTCTCGCAGGAGGATCAGCTGAGGAAGGAAATGGAAATGATGGAGATGG
TGTGTAAAGGGCTTGCTGGATAA

>MiMYB17

ATGGATCGGGTTTCGTCGTCTGATAGGATCAAGGGGCCTTGGAGTCCTGAGGAGGACCAGC
TGTTGCACCAACTCGTGCAACGTCACGGCGCGAGGAACTGGTCTCTTTTATCCAAATTGATT
CCGGGTCGGTCCGGGAAATCCTGTCGGTTACGATGGTGTAATCAGCTCTCTCCTGAGGTCC
ACCACCGCGCCTTCACGTCCGACGAGGACGAGATCATCGTTAAAGCGCATGCCAAGTACGG
CAACAAATGGGCTACCATAGCGAGACTCTTGAACGGCCGTACCGATAACGCGATAAAGAAC
CACTGGAACTCGACGTTGAAACGGAAGTACGCGACGATGACGACGGAAGATGAAAACGA
CGATACCAACGAGAGAGAAAGTTTTCTTCATCAAGAGAAGAAAAGATTAGCGGGTGTTTCG
TTTTCTGATATTTCTTCTTGTCCGTCTGGATCCGACGTGAGCGATTCGGGTCTACCCATGATG
TCTGGTTCGGATATGTGTGTGCCTACAGGAAGGTTTGTTGGAGCCGAGACTGGGAACGATG
GAAACAATGGCAATGATGATGCTGTGTTCAATGTCTCGACTGAACTTACTTTGAATTTCCCT
GGTAACAAGTCAACCGAGTTGAGGGAGAACGATTTCACTCGGTGTTGGGTGCGGGGGGAG
GTTTCTCAGAAGGAGAATAACAGCGAGTATGATAATCATAAAACGACGGCGTTTGGGCCGG
AGTTGATGGCTTTGATGCAAGAAATGATAAGAAAAGAACGTCTTTTGATTCCCTCCGGTGA
GGAACCGGTCCTTGACCTGACACATGTCGGTTTCATTATATTATCATCCGAAACCTATCTTCT
GTGTATGATTTCAGAAAAAAATCGAGTACCCGACAGTTTTGAGTTTGCATGGGTAAAATACT
CAGCCCCTTATAGAGCTGAAGAAGATCTCCGATCAGAGCCAAATTTAAAATCGACGAAGAA
GAGGATAACAAATAGCGAAAGAGCTTTACAGAGAGAAGAGATGCTGCCAACTTCAGTCTCT
AAAGGACGTTCTTCGTCCGCCACGTCACGATCAAACTCCATGCTCCCACAGTACCTTCGTC

GAATCATCAAGGTCCTCGCTAAAAATTTCTAA

>MiMYB18

ATGCCTCCAGAACCACAGCCCTGGGATCGGAAAGACTTCTTCAAAGAGAGGAAGCACCAT
AACAATAATAACCACCACGACAGGACGGCGTCTGACTCCTTGCTAGGAGGTGGCGGCGGT
CCCCTGACCAGATGGAAAGATTACTCCTCGTTCTCTTCTCACAGTCGAGAGCTCCCCCGCT
GGGGATCTGTCGACTCTCGCAGACCTACAGCAACAGGAAACCTAAGCCTTGTTTCGACCAC
AGAGATAATTAATTTCACTAGCAAACTGCTTTCAGATTCCCAGATCAAGGTTTATAGGGATTT
TTTGAAGATGCCTGCATTGATATTGGACAAGAAGGAGAAGATGGTGTCAAGGTTTATCTCTA
GTAATGGACTGGTTGAAGATCCATGTGCTGTGGAGAAGGAAAGAGCTATGATCAATCCCTG
GACAGTGGAAGAGAGAGAAATTTTCATGAATAAATTAGCCACTTTTGGGAAAGATTTCAGG
AAAATTGCATCATTTCTTGATTATAAGACGACTGCTGATTGTGTTGAGTTCTACTATAAGAAC
CACAAGTCTGATTGTTTTGAGAAAATAAAGAAAAGGCCTGAACAAATGAAGCAGTGCTCTA
ATACCTATTTAATTGCATCAGGCAAAAAATGCGATCGGCAGATGAATGCAGCATCCCTTGATA
TATTGGGTGAAGCTTCTGAGATGGCAGCTACATTCCAAGCCAACAATGCTCGTCAGATTTGT
TCTGTTAGAATCTCTTTGGGAGGGCGTAGTGATTCAAAATTGTCTTTGGGTGATGATGGCAT
GACAGAAAGGTCAAGCAGTTTTGATGTGCATGGGAATGAGAGAGAAACTGCTGCTGCAGA
TGTTTTAGCTGGAATATGTGGTTCCTTGTCGTCTGAGGCAATGAGTTCTTGCATCACAAGCT
CTGTTGATCCAGGGGAGGGCCAACGAGAGTGGATGCACCAGAAAACGGATTCTGTGAGGA
GATGGCATTCAACATCTGATGTTTCTCAGAATGCTGCTGATGACACTTGCTCAGATGACAGT
TGTGGGGAAATGGATCCTGCTGATTGGACAGATGAGGAGAAATCCATCTTTATACAGGCGG
TGTCATCCTATGGTAAGGATTTTGCCATGATCTCACGATGTGTTAGAACAAGGTCCAAGGAT
CAATGCAAGGTTTTTATAGCAAGGCACGAAGGTGCCTTGGGCTGGATTTGATACATACCGG
ATGTGGAATTGCAGGAACCACTGTAAGTGATGATGCCAATGGTGGAGGGAGTGATTGTGAA
GATGCATGTGTCTTGGAGGCTAGCTCAGTCATCTGCAGTGATAAGGTGGACTCTCAAATGAA
TGACTTGCCATCCTCTGGGGTTAACAAAAATCAGGACCCATCCTGTGCAGGACCCATGAGC
TTACAAACTGACCTGAATAAAACAGAGGATGACAATGGGGTGAGACATTTTGATGATCCCA
GATCCAAGGATGTAGGTCCTTTGGTTTCTGATGAATGTAAGATACTGCATAGTCCAGAGGTA
GTTTCTGAATTTGAGAGAAGGAAAATGGATGATGTTGATAGGCAGTCCAAGTCACTGCAGG
CCCAAAAACTTCCTGTCTTATTGGTTGAGAAGGAAGTTGAAAAAGATAAGTTGACAGAACA
AGCTGTATGTGTACCAGTACCAGCAATGATTAGAGAAGCCTTGAAATCATACCCGCCTGGTT
TAAATGCTGTAGTTGAGACAACTGAGGCTCCTGTTAAGGGATTCCAAAATGGTTTGGAAGA
ACAAAATGAACATAAAACATGCTCAGCTGATAGTAGTGACAGAGATATGATGCGAGGTTCA
AATGTTGGGAATATTTTTGATCTGGCGATAGAGACAAGTTCTTGTTCTGTTCCTCTGAAGTT
GGATAGTGGGGATAAGCTTCCTGTAATGTCATTGCCACCGGAGAACTCTCTTGCTCCTGCAA
CTTCTGTGGCACATGATTCTGCTGCCATTGAATGCAAAAAAACTGCTAAACAAAATAGATTG
TCTACCAAACTTGACTTTGAGGGGAATAAAGATAAGAATGCTGATATATCTGTTGGCAGTGA
AGACTATCACCATAACCGTATGGGGCATTCCATAGTGAACCATGTTGAACACTCCCAGATTC
TCAAGAGGTACCTGTCGCAGATTTCAGCAAAGAAAGGGATGAATGGTGATATTGGCTGCAG
ACAACTTTCTGAAGTTCAGGGCATCTCTAAACCAGACAGGAGTAACAGTGTGCACGTGGCT
CAGGGTTGCCACGTTCAGAAGGGTACTAGTTCTCTGTCTCATGCAGCAGTTACTGAGTTTCC
TCTTGTGTTGCCAAACTTAGACCAAAAGAATGATCCACCAAGAAGCCATTTGCTCGGGTTA
ACAGAAGTGGATAGACCATGCAAGAATGGTGATGTTAAATTATTTGGTAAGATTCTTAGTCA
TCCCTCATCTTCACAAAAGCCAAATTCCTGTAGTCATGAGACTGAAGAAAAGGGGACTCAT
CATCACAAACAGAGCTGCAAGGTATCAAATATGAAATTAACTGCTCCTCGCCCAGCTGAGA
TGTTGAAGTTTGATTGTAATAACTATCCAGGTCTTGAGAATGTTCCTCTGGGGAGTTACAGA
TTTTGGGACGGGAACAGAATACAAACTGGCTGTTCATCTTTGCCTGATTCGGCTGTTTTACT
GGCCAAGTATCCAGCAGCCTTGGGCAATTATCCTGCTGCCTGCTCATCCAAACTGGAGCAG
CAGGCTTTGCAAGTCAAGAGTAGTGAGTGCAATATGAATGGTGTAGCAGTTCTTCCTCCAA
GGGAAATTAGCAGCAGTAGTAATGGAGTAGTGGACTATCAGGTGTATAGGAGTCGTGATAAT
AGTAAAGTACAGCCATTTACCGTAGATGTAAAGCAGAGGCAGGACTTGTTATTCTCTGAGAT
GCAGAGACGAAATGGATTTGAAGGTCTCCCGACTCTTCCACAGACAGGACGAGGGATGGT
TGCGTTGAATGTTGTAAGAAGAGGAGGGATACTGGTAGGTGGACCATGCCAAGGTGTTTCA
GACCCAACGGCAGCCATTAAAATGCACTATGCTAAAGCTGATCAGTATGCTGGGCAGAGTG
GGACCATTATTCGGGAGGAAGAATCTTGGAGAAGCAATGGGGACTTAGGAAGGTAG

>MiMYB19

ATGCAATTGTTTCTTGATGAGGTATTGGAGAATGGTTGCAGCTTACAAGAGGTCTTCTGTCA
TGGCAAAATAAAACTTCTTAATATGGGAACTCTGTATCCAGCATTATATATGTTGGATTCAAG
TTGGATTCTTCAGGAAAGCCACAGCACAAGCTGGACTAAAGAAGAGAACAAGAGGTTTGA
GAGTGCTCTTGCAATTTACAATGAGGGCACGCCGGATAGATGGATCAAAGTGGCAGCTCTA
ATACCAGGAAAGACAGTGCTTGATGTCATTAAACAGTACAAAGAATTGGAAGAAGATGTGA
AGAATATAGAAGCTGGAAAGGTTCCAATTCCACGTTATTATGGCTCATCTTTCACATTAGAGT
TTGTTTCTGAACGTGATTTTGATGCTAATAAGAAGAGACCATTGGTGAAAAGCTTGGATCAA
GACAGGAAGAAGGGTGTGCCTTGGACTGAAGAAGAGCACAGGCTGTTTTTGAAAGGACTT
CTTAAGTATGGTAAAGGAGACTGGAGAAATATCTCCAGGCATTTTGTAATCACTAGGACCCC
TACTCAAGTGGCAAGCTATGCTCAGAAATACTTTATAAGGCAGCTATCAGGAGGGAAAGAC
AAGAGGAGACCAAGCATCCACGGCATTACTACCATCAATCTTGCTGATCCAAATTCATCAGA
CAATCGAAAACCTTGTTCTTTTGATCTATCCGATGTGCTTCTGCAGCAACAGAAGTCTTCTG
ACTCGCCAGAAGTTGGACTCAAATGGAATGATTCAAACAAAGGAGCAGTGAATTTAATACG
TCGTGTGATAACCTTTTTGTGCCGTCTGCCTTTGACATTGGTTCAAATGGCCTCAAACTTCA
GGGAAAAACTTTGTATGCCACTGTTTATCATGGGATTCAGTTCAACCCGAAAATCAGTGTTC
TGA

>MiMYB20

ATGTCGGGTTCAAGTTGGTTTCTTCAGGAAAGCCAGAGCACAAGCTGGACTAAAGAAGAG
AACAAGAGGTTTGAGAGTGCTCTTGCAATTTACAGTGATGGCACGCCGGATAGATGGCTCA
AAGTTGCAGCTCTAATACCGGGAAAGACAGTGCTTGATGTTATGAAACAGTACAAAGAATT
GGAAGAAGATGTGAAGGATATAGAAGCTGGAAAGTTTCCAATTCCGGGTTATTATGGCTCAT
CTTTCACATTAGAGCTGGTTTCTGAACGTGATTTTGATGCTGATAGGAAGACACCATTGGTG
AAAAGCTCGGATCAGGAGAGGAAGAAGGGTGTGCCTTGGACTGAAGAAGAGCACAGGCT
GTTTTTAAAAGGGCTTCTTCAGTATGGTAAAGGGGACTGGAGAAATATCTCCAGGCATTTTG
TAATCACTAAGACTCCTACTCAAGTGGCAAGCCATGCTCAGAAATACTTCATAAGGCAGCTA
TCAGGAGGGAAAGACAAGAGGAGACCAAGCATCCATGACATCACTACTGTCAATCTTGCT
GATCCAAATTTATCAGACAATCGAAAACCTTGTTCCTTCAATCTATCCCATGAGCTTCCACA
GCAACAGAAGTCTTCTGGCTTGCTGAAAGCTGGACCTGAATGGAATGATTCAAACAATGGA
GCAGTGATTTTTGATACATCTCACGATAACCTGTTTGTGCCATCTGCGTTTGACATTGGTTCA
AATGGCCTTAAACTTCAGGGAAAAAGTTTGTATGGCACTGCTTATCATGGGGTTCAGTTCAA
CCCGGAAATCAGTGTTCTGAATCCAATTTAG

>MiMYB21

ATGACCGATAAAGAGAACGAAAGCAACGGTGACGCGGAGGTTGTTGCGGAGGAAACGGT
GGAGGTCGGCTGCGGCGGAGGTGGTGGAGGTGGAGGTGCGGAGGGGAGTCGTAATGGAG
GAGCTAGTAGAGTGAAGGGGCCGTGGTCGCCGGAAGAGGACGCGGTGCTGAGTCGACTG
GTGAGTCAGTTCGGGGCGAGGAATTGGGGGATGATCGCGCGAGGAATTCCTGGTCGGTCTG
GAAAATCGTGCCGACTTCGCTGGTGTAATCAGCTTGACCCTTGCCTTAAGCGCAAACCTTTC
ACTGATGAAGAGGATCGTGTTATAATTCAAGCCCATTCAATCCATGGGAACAAATGGGCAGC
AATAGCAAGGCAACTGCCAGGTAGAACAGACAATGCAATCAAGAACCACTGGAACTCTAC
CTTAAGGCGCAGGTACGCAGAGATTGGAAGGTTTAAACCAGGAAGTGGTGACATGATGGA
AGACGGGAGTCATGACAGAACAAAGGTATCCTCAGAAGAGACCCTATCTGCTGGAGATGTT
AATTCATTCAGGCCCCCAGAAGGAAGAGATGTCATGATGGATGATAGTCCTAACCAGCATG
AAGACATTGCTCAAACAAAGGAGTGTCAGTTATTTGCAGGACCAAATCATCGTCCAGCCTT
TTCACGTCCAGTTGGTGATATGAGTCCAATCAGTCTCCCAGAAGGAAGAGACATAACAATG
AATGATGGACCAAATATGCATGAAGGGATAGCTCACAGAAAGGAAACTGAAGGTGATTCTG
GACCAAAACATCCTACACTTCCTCGTCCAGTTCCTCGTGTTAGTGCTTTTAGTTTCTATAACC
CTCCAAGTGGCCCAACATCTAGCTCTGAGATTCCATGGACAATCCCAACTCAGGGACCTTT
GGTTCAAGTACCCAGACCAGACTTTGGTCTCAAATTCGTAGAAGATGTTCATAGTGAGCCT
GTTATTCCTTCGCAGTGTGGTTATGGTTGCTGCACATCTCCATTAGGGCACCATTCACATGGC
TCCCTACTGGGACCTGAATTTGTGGACTATGAGGAGCCTCCTGCTTTCTCGAGTCATGAATT
GATCTCTATAGCTACAGATTTGAACAACATTGCATGGATTAAGAGTGGCCTCGAAAGTTGCA
GTGTCCTTGTACCAGGCAATGCTGCAGCAAGCCACAAAATGAGTGAAGGGACTACTGACA
GCTCGCAAATAAGAATATCAGAACGAGGGGTAAGAAAAGATATGCACTCTGATGAGGGACG
AAACAAGCTGATGGGCATGATGACAGACGTCCTATCTGTTCAGGTGCCGGCACAAACTTAT

GCCGGGCGACCTGAGGTTGAGGGTTTGAGCTAA

>MiMYB22

ATGGACGATTCTGGAGCTGCTTCTGGTGATGATGCCACCAAGACTTGCCCTAGAGGCCACT
GGAGACCCGCTGAAGATGAAAAACTCCGCCAACTTGTTGAACAATACGGTGCTCAAAACT
GGAATTCTATTGCTGAGAAGCTCCAAGGAAGATCAGGGAAGAGTTGTAGATTAAGGTGGTT
TAATCAGCTTGACCCCAGAATCAACAGAAGACCATTTACAGAAGAGGAAGAAGAAAGACT
TCTAGCGGCTCATCGAATTCATGGAAACAAATGGGCATTAATCGCCAGACTATTTCCAGGAA
GAACCGATAATGCTGTGAAGAATCACTGGCATGTTATAATGGCGAGAAAGCAAAGAGAACA
ATCAAAGCTATGCGGAAAGAGAACTTATTCAGAAAGTCTTAGCAGCTCCAACGATATCAGT
TTCGATCATTTTAGCAGTAATCACATTTCAAGAAAATTAAGACCCCAGGACATGTTTATTGGC
TCAAGAAATATCGGGTTTGCAGCCTCACCTGCTGTTTCTCAGCTTTCTTGGACTTTCGCCCC
GCCAATGGTTGCAACTTCAAACAGTAATATTTCATCTTCAGCAGTGGAGTTTAAAAAAAAA
GAAGGAAGAGATTATTTAACTTCTACTTCTAATTACTATTCGAGTTCTTCAGTTCGTAGTTCT
AGTTCTATAATTGGACTCACAAACAGCAGGAGAATCGTTAGTCCATTTGGTTCTTTTAGAGT
TGGTGATCATCTTGATTATGAAAGCCATGGGAAAAAGAAGGAGCTGATTAAATTTTCAGATA
ACTCCATGAGAGTGAGTGCAGTTCAGCAAGCTCAAGGAGACGAATCTATTCATCACAAGAC
TGTTCCTTTCATAGATTTTCTCGGTGTGGGTATCTCTTCTTGA

>MiMYB23

ATGTGTTCTTCTTCTACTTCATCCTCGGATTCTTCTTCTTCAGAATCATCTTTTTCTGGTAATA
ATAGAATACCAAGAGAAACCCACAAAGCTGAAAGAATTAAAGGTCCATGGAGTGCGGAAG
AAGACCGTATTTTGACCCGTCTTGTTGAACGATATGGACCGAGAAACTGGTCTCTTATAAGC
CGATACATTAAGGGAAGGTCGGGGAAATCTTGCAGGCTTAGATGGTGTAACCAGCTAAGCC
CAAATGTTGAGCATAGGCCTTTCTCTCCGGCAGAAGACGAGACCATCTTGGCCGCCCATGC
CCGGTTCGGCAACCGTTGGGCCACCATCGCACGGTTGCTCCCTGGTCGAACAGATAATGCG
GTCAAGAATCACTGGAACTCCACGTTGAAGAGAAGAGCAAAAGAACAACAATTACAACAA
CAGCAACAGCCGCTAGAACAACAACAAATGGATTTTTTTGATAATGGAAATAATAACATAGG
GCATGCAGCTTCAGGATCGGGTCAGCAGTGTGTGGGATTGGAAGATGATTCGTTGACTGCA
TTGACTTTGGGTCCTCCAGGGAGTAGTAGTGCTGCGGTGACGGAAAGGAGAACGGAGGGT
TTTCCGGCGGGGTTCTGGGATGCGATGAGAGGTGTTATAGCAAGAGAAGAAAGTGGTCGCC
CTGGCATCTGA

>MiMYB24

ATGGCAGTTTTGATGGAGAGTGATTATACGTGTAAGTTGGGTAGACCAGGAGATGACGCGT
GTCTATTTGACAGCTGTATACTAGTAACTGCTCTCAACGGCTATGACCGGTTTTTTTTCTTA
AAAACCTTAATTCCATGGCTTCAACAAGAAAGGATGTGGATCGGATCAAGGGTCCATGGAG
CCCTGAAGAAGACGAAGCGTTGCAAAGGTTGGTTCAAAGCTACGGTCCACGAAACTGGTC
TTTGATCAGCAAGTCGATTCCTGGTAGATCGGGAAAGTCCTGCCGTTTACGGTGGTGCAATC
AACTATCACCTGAGGTAGAACACCGGCCATTCACAGCTGAGGAGGACGATACTATAATCCG
GGCCCACGCTCATTTCGGTAACAAGTGGGCTACCATCGCCCGTCTTTTAAACGGCCGGACC
GATAACGCTGTTAAAAATCATTGGAACTCTACTCTAAAGAGGAAATGTTCTTCGCTCAGCGA
TGATTTGAATGAAGATGCCAACAATATCCAACCATTGAAAAGGTCGGCCAGTGTTGGGCCG
GCTAATAATATTTCGGGACTTTGCTTGAACCCGAGTAGCCCATCCGGATCCGATTTGAGTGAT
TCCAGTTTGCCGGGTGTTCAATCTCCAGTTTACCGACCATTGTTGAGAACCGGGTCGCTTGC
TCCAGTTGAAGCCTCTTCATCCACCACGGATCCTCCCACTTATCTCAGCCTCTCGCTTCCTG
GATCAGGACTGGATTCGTGTGAGTTCTCAAATCATGGATCTGGATCCGCATCCAGACCCGTT
TTGAGTTCAAATAATATGGTACAGCTCCAGGTGGCACAGCCACCTTTGGTACAGAGTCACA
GTGAGGGTTTTGAAAAGCAGTTCTTTAGCGCAGAGTTCATGGCTGTCATGCAAGAGATGAT
AAGAAAAGAAGTGAGGAATTACATGTCTGGAATTGAACAGAACGGGCTGTGTATACATACT
GAAGCTATTAGGAATGCTGTGGTGAAGCATATGGGTGTTAGCAAGATCGATTAG

>MiMYB25

ATGGACGATTCTGGAGCTGCTTCTAGTGATGATGCCACCAAGACTTGCCCTAGAGGACATTG
GAGACCAGCTGAAGATGAAAAACTTCGCCAACTTGTTGAACAATATGGTGCACAAAACTG
GAACTCTATTGCTGAAAAGCTCCAAGGAAGATCAGGGAAGAGTTGTAGATTAAGGTGGTTT
AATCAGCTTGACCCCAGAATCAACAGAAGACCGTTTACAGAAGAGGAAGAAGAAAGACTA
CTGGCGGCTCATCGGATTCATGGAAACAAATGGGCACTAATAGCCAGACTATTTCCAGGTAG
AACTGATAATGCTGTGAAGAATCATTGGCATGTTATAATGGCAAGAAAGCAAAGAGAACAA
TCAAAGCTATGCGGAAAGAGAACTTATTCAGACACTCTTACTAATTCCAATCAATTTAGTAAT
AATCATATTTCTAGAAAATCGAGACCCGAAGACATGTTTTTAGCTCGAGAAATATTGGGTT
TCAGAATAGTAGAATCTTTGATTTCCGAAACACCAATTCAGATATTAACAGAACGTTTGCAG
TCTCGCCTTCCGTTTCTCAGCTTTCTTGGAGTTTCGCCCCACCAATGGTTGCAACTTCAAAC
AGTATTATTTCACCGTCATCAGTGGATTTCTCAAGAAGAGAAGGAAGAGATTATTTGACTTA
TCCGAGTTCTTCAGTTTATGGTAGCAATTGGAGTTCTTCTATAATTGGACTCACAAACAACA
GGAGAATAGTTCAACGTCCATTTGGCTCTTTTAGAGTTGGTGATCTTCATTATGATAGCCACG
GAAAGAAGGAGCTGATTAAATTTTCTGATAACTCCTCGTTGAGAGTGAGTGCAGTGCAGCA
AGATCAGGGAGATGAATCTATCAAACATGATAGTGTTCCATTCATAGATTTTCTCGGTGTGG
GTATCTCTTCTTGA

>MiMYB26

ATGCAAGAAATCACGAAGAAGATTGGAGGTGCTAATGAAGACTCCAAGAAGAAGGAACGA
CATATTGAGGATAACATACTGAGGGAGCAGATTACTATACATGGAACTGATAATTGGTCAATT
ATTGCATCTAAATTTAAAGATAAAACAACGAGACAGTGTAGAAGAAGATGGTACACATACTT
GAACTCGGATTTCAAGAAGGGGGGATGGTCACCAGAAGAAGATATGCTTTTATGTGAGGCC
CAGAAAATATTTGGTAATAGATGGACGGAAATAGCCAAGGTGGTTTCTGGGAGAACTGATA
ATGCTGTGAAGAACCGGTTTTCCACATTGTGCAAGAAGAGAGCAAAATATGAAGCTTTAGC
TAAAGAGAATAACAATCCATATATGAACCAAAACAACAAGAGAGTTTTATTCCAAAATGGG
TTCAATGCAGATGGAACACGAGAAAATACAGAACTCATTAAGAGAGCTAGGAGGTCACAC
ATCCCTGATCTCGCAGAAAGCCACAACATTGCAAACAGATCACATAGGGAATCTGGAACAA
CAATGAATCCACGGTTCAGACCTCCATTTACAGTGTTGGTTCAAAACTTACCCAGTGTAAAC
GGCTTGCCAGCCTCACATTGTGATATGAAGGAGGTGTCAAATAATGCAGCTCAGAACAATA
AGACTCAAGGAATGTTTCTTAGAAAGGATGATCCAAAGATAACTGCTTTGATGCAACAAGC
AGAACTTCTTAGCTCACTTGCGCTAAAAGTTAACACAGAGGACACAGAACAGAGTTTGGA
AGATGCATGGAAGGTTCTTCAAGATTTCCTGAACCGAAGCAAAGAAAATGATATCCTAAGA
TATACAATCTCTGACATCGATTTTCAGTTTGAAGATTTTAAAGATTTGATAGAGGACTTAAGG
AGTACTAATGAGGAAAGTCATCTGTCCTGGAGGCAACCAGATCTATATGAGGACTCTCCAG
CCAGTTCTGAATATAGTACTGGAGCAACTCTAATTCCTCATCCAGCCTGTGATAAACCAGAT
CAAATTCAAGCTGAGGTAGATGCACTGCATCGGGATATTGTCGCTGAATTGCAATCAATTCA
TATTGGCGAGCAACATTGCTTGGGCCAGCAAAATAAAGGGAGTGCAAACACAAATGAAGT
GGTGTTATTCCCCTCTGGTGGTGAAGACACGAACAACACTGGAGCTGTCTCTGTCTCATCA
AGTACAGCGTTCAGTTCCCCCCTCCAGGTTACTCCACTATTCAGATCCTTAGCAGCAGGGAT
CCCCAGCCCAAAATTCTCAGAAAGTGAGCGGAATTTCTTACTAAGAACACTTGGAATTGAG
TCACCTTCCCCCAATCCAAGCACCAATTCTGTCCAACCACCTCCCTGCAAAAGAGCCCTTC
TCCAAAGTCTATAA

>MiMYB27

ATGGCTTCTCTATCTCTTAGTCCCAATTATGGAAGTGCAGTTTCGGTTGTTTCTGCTTCACTA
GAGACTCATATTTGCAGGAACACCGATATGGAAAATGCGGGAGCTTGCTGGGGTTTTCCTTT
CATGGGTAACAGCTCAACCAGAAGCTTCGAGGGACAACATAATATTGATGCTAATGCTGAG
GTTAAGTGCTCTGATTATGATGATAGATTGGGAGAAGCCAGTGAGACTATTATCCACAATGT
CAACTTGAATGAAGAGTTTAACCCTAACGAGAAGGAAATAGATAGTGGGCAGGCAAAGCT
TTGTGCTAGAGGCCACTGGAGGCCTGCGGAAGATACCAAGCTCAAGGAACTTGTTGCTCTT
TATGGCCCCCAAAACTGGAACCTTATTGCCGAGAAGTTAGAAGGTAGATCAGGTAAAAGCT
GTAGGCTGAGGTGGTTCAACCAGTTGGACCCAAGAATCAACAGAAGAGCTTTTACCGAAG
AAGAAGAAGAAAGGTTAATGCAAGCTCATAGACTATATGGTAACAAATGGGCTATGATAGC
CAGGCTCTTCCCTGGAAGAACTGATAACGCAGTCAAGAACCATTGGCATGTTATAATGGCTA
GGAAGTATAGAGAACAATCCAATGCGTATAGAAGGCGGAAAATGAGCCAGTCTATTTACAA
AAGAATGGAGGATATCCCAGGGGTTATTTGCAGAGATGCAACCGCCAGAACTGAACCACAA

CCCTACTGTTTCAGTGTCCCCAATGGAGGATTTGGTAACACCTCTTCTTTCTGTGGTTCTGG
TGGGGTTGAATTCGCCTTCAATGGTTCACCCCACATGACCAGTGGGCAAGAAACAATCTTA
AGCAACAAGGATCCTTACAGTGGGTTTTGTGTACAGCAGACACCTAGTGATTTCTTTCCTGG
CCCCAAGGGCAGTGACATGATGGCCATGTTCAGCCAGGTGAGATCTTGGGATGGGCCAATT
GATGAACCTCACGTTTATGGCTTCAATCTTCACCACCACCCTCAAAACTTGATGGCAATGCA
ACAGTCAAATTTCCAACACCACCAACTTCACAGTTTCTTTGGTTCCACGGCATCCACACCTC
AAGTCTCAGCCAGTGAAGCTTCATCATCAGTAGACAACCATTTTGACCCCGTTCCACCACG
CTTCTTTGATTTTCTCGGGGTAGGAGCCACATAA

>MiMYB28

ATGACTGTGGATGAAGTGGGCAGTAGCTCCGAATGGACAAGAGAGCAGGATAAGGCATTT
GAAAATGCTCTGGCAACTTATCCTGAAGATGCTTCCGATCGATGGGAGAAAATTGTAGCTGA
TATACCTGGGAAAACTCTGGAAGAGATTAAGCATCACTATGAGCTTTTAGTGGATGATGTTA
ACCGCATTGAATCAGGTTGTATTCCTCTGCCTAGCTATAAATCATCTTCTGATGGATCTACAG
GCCATGGTGGTGATGAAGGAACTGGAAAGAAAGGTGGCCACTATGGGCATTATAACAGTGA
GTCCAACCATGGAAGTAAGTCTTCACGGTCAGATCAGGAACGCCGTAAAGGGATTGCTTGG
ACAGAGGATGAGCACAGGTTATTTCTTCTTGGTTTGGACAAATACGGTAAAGGTGACTGGC
GCAGTATATCTCGCAACTTTGTGGTTACAAGAACACCAACACAGGTGGCAAGCCATGCACA
AAAATACTTCATCCGATTGAACTCAATGAACAAAGATAGGAGGCGATCAAGCATTCATGATA
TCACCAGCGTTAGCAATGGAGACATTACAGCAGCCCAAGGACCAATAACAGGTCAAACCA
ATGGTTTGGCTTCAGGAGCTTCTTCTGGCAAATCAGCTAAACAGCAACCCCAACATCCAGC
TGGGCCACCAGCTGTTGGCATGTATGCTACTCCTACCATAGGTCAGCCTATAGGAGGACCCC
TTGTCTCAACCGTTGGCACCCCGGTGAATCTTCCTGCTCCAGCACACATGGCATACCGCAAT
AGAGCTCCAGGACCTGGAGCAGTGGTTCCTGGTGCACCAATGAACGCTGGTGCTATGCCTT
ACCCGATGCCGCACACATCTGCTCATAGAAGTTGTAAAGAGCCTGTCTCTCATAAGTCTATT
TACATTACTGTTTTTGAATTGCCTTGCATGATTGGTGGGCTGTCAGGCATCATTTTCTCCATT
GAACTTGGGATTTCAACAACTCGCTTTCTTACTATAAAAAATACCTTTCGTTTCCACTAA

>MiMYB29

ATGGAAGCTCACATGGGAGGTTTTAGTTCTTCAACTTGCGATCAACGAAGTGAAGAAGAGG
TAGATGTGAGAAAAGGGCCTTGGACTTTTGAAGAGGACTCATTGCTTATGAATTACGTTACC
ATTCATGGCGAAGGTCGTTGGAACTCTGTCGCTCGCTGTGCAGGACTAAAGCGAACGGGA
AAAAGCTGCAGGTTAAGGTGGTTAAATTACTTGCGCCCGAATGTTCGAAGAGGAAACATCA
CTCTGCAAGAACAACTAATGATTCTTGAACTCCACTCCCGCTGGGGAAATAGATGGTCAAA
AATAGCACAATACTTGCCGGGAAGAACAGACAATGAGATAAAAAATTACTGGAGAACAAG
AGTTCAAAAGCAGGCAAAGCAGCTCAAATGTGACGTTAACAGTAAGCAATTTAGAGATGC
CATGCGTTACGTTTGGATGCCTCGTTTACTTGAGCGGATCCGAGCAACAACAACCGATTCAT
CAACAATTCAACCCACTTCAAACCTCACCACCACCACAGAGAGTACAAGTACTCATGAAGA
CAAGTCCACGAGTCAAGTTCATCACTCCACTGAGTTGTCTTGCTCTTCCTGGGACTCAGTG
GATGCCCACGTTTCTCCTGCCTCAGATGCCAATCTGGACAATTCACAAAACTGGTCGGAGC
TTTACCTAAACAACATGTCAGGCTGTGTCTCCGGGTCATGGGACTGGTCAAAACCGGGCTT
GGATATCAACGGATTCGAAACTCAGAACAACAACGAGTGGATAGGAAGCGATGAGTCATTC
GAGAGCTTGTGGAACGACGAGAATAACTGGTTGTGGCAACAGCAGCAATATGATCATTAG

>MiMYB30

ATGGAAGGAAGAAGAGAAGAAATAAGAAAGGGGCCATGGAAAGCAGAGGAAGATGAAGT
TTTGATAAACCATGTGAAAAAGTATGGCCCAAGAGACTGGAGTTCCATTCGATCCAAAGGA
CTTTTACAGAGAACTGGCAAGTCTTGTCGACTTCGTTGGGTAAACAAACTTAGACCCAATTT
AAAGAATGGGTGTAAATTTTCACTAGAGGAAGAGAGAGTGGTGATAGAGTTGCAGGCACA
GTTTGGGAACAAATGGGCAAGAATAGCCACGTACTTGCCTGGGAGAACTGACAATGATGTT
AAGAATTTTTGGAGTAGTAGGCAAAAGAGGCTGGCTAGAATTTTGCAGAATTCAGCGACAC
CCTCTTCCTCTTCCAACTCCAAATCCCACAGGGCTACTAGGGAAGTTTCTACTTTCAATGAT
GTTCCCACTTTACTGGCTCCAAAATTGAGTTCTTCAATGGATGAAGAGTCATCCTCAAAGGA
TCATTCTTGCTCGTCATCCAACAGAGAGAAAGCTGGGACAATTAAGATGGTTCCAGTGCCG

GCTCTAGTTAATCCTAAGTTGCTTCATTTCGATGCCCATTCTGTGCTTACTCCATTTGAGAAT
AATCCTTGCACTGATTCTCAATTACAAATTCCTTTTCCTGACATTACACAAACTCAGCATGAT
CTTACATTTTCACCAGAAAGCCAAGAGCTATTGGCCGGGCTTGAGGACCCTCACTTTTTCG
GTGTGTTTGGACCGGTGGATGCTTCTCAACTTAGTAATGAAGTGCCGCTTCCTGTTGAAGA
ACCATTTTTAAAGCCAATGAGAAGTTTTAGGAACGCTGTCAAGGACGAAAACGATAACCTG
GTGAACCCAGATGCCTTCTTTGAGGACTTCCCAACCGATATGTTTGATCATATCGAGCCACT
TCCCAGCCCATCAGACTGGTGA

>MiMYB31

ATGAAACCCTGCATAAAAACTGAGATTCCTTTTGATGAAGGACCTTCTTCCAAAGGCTACCT
TCAAGATTTTCACCATCTTGATACCCATTTTCGAGCAAATGGGTGTTCCTCAAATCCTATGTT
TGGAGTCCAGACTCCATATTTGGACTCTTTGGAAGCTTTCTCTGCTGCATATCCTTGCTCCTT
ATCAAACTTTGATGTTTATGAGTGTAATCCATTTGCTGATAATTTTCACAGTGGAGGATCCTT
GGAGCATCATCAAGTTATAAATCCTCAGCTGGTTGATATTACGGGATCCAGTCAGAGCCGCA
TGCCAATAAGTTATCAGGAAATAAAACCCGTGAATTTTGCAGGATCAGATGAAGTTTCATGC
GTAAGTGCAGATGATATTGCTAGTTACTATAAGAAAGCTAACATGGATACGATTAATAGTAAG
GCCTATCTTTCGACAAGGTCACTGAAAGCTGGTAAGAAATACAAAGTAGTGAAGGGGCAA
CGGACAATTGATGAAGACAGGCTCTTGATTAGACTGGTTGAACAGCATGGCATGAAAAAAT
GGTCTCATATTGCGCAGATGTTGCCTGGAAGAATCGGGAAGCAGTGCAGAGAAAGATGGCA
TAATCATCTGCGGCCTGATAGCAAGTTTACTTTCTTTCATTTGCAACATGGTATCAGAGGAGG
GGCTCTCTCCTTTCTTCTCAAGGATACTTGGAGTGAAGAAGAGGATAAGGCGCTAATAGAG
GCGCATGCAGAGATAGGCAACAAATGGGCAGAAATAGCAAAGAGGTTGCCTGGAAGAACA
GAAAACTCTATCAAAAACCACTGGAATGCAACCAAGAGAAGGCAATTCTCCAAGCGAAAA
TGTCATTCAAAAAACCAGACAACCTCCCTTCTACAAGATTATATTAAGACCTTAAATTTTACT
TCAACCTCTACCAAACATCAGGCGAAAACTTCCTCCAAGTTAGTACTAGAGAAGACACCTG
CCGATGATGACTTCTGCTTGAGTGATCACTTGGTGTCTTGTGCTCCTGTTGATGCTGATAAG
AAAAGTCTTGAAATGGAGTTGCCATTAATAGACACGGAATATCAAGTGAAGAAGGACCTGG
ATTTGGTGGAGATGATTGTTGAAGCTAATAATCTGTAA

>MiMYB32

ATGGCACCCAAAAATGACGGATCCGCAAAGAAAGTAATGAACAAAGGCGCTTGGACGGCC
GAGGAAGACAGAAAACTAGCTGAGTATATTGAAATTCACGGAGCCAAGAGGTGGAAAACC
GTTGCTACCAAATCAGGTCTGAATCGATGCGGAAAGAGCTGCAGATTAAGATGGCTGAATT
ACTTGCGACCCAACATCAAAAGAGGCAACATTTCGGACGAAGAAGAAGACTTGATTCTTC
GACTCCATAAATTACTTGGCAACAGATGGGCGTTGATTGCTGGGAGACTTCCAGGACGAAC
GGACAACGAAATTAAGAATTACTGGAATTCTCATTTGAGCAAGAAAATAAATAAGGTGAAG
CCGCCACAACCAGAAATTGTGCAAGAAGCTGCCCTTCAAGACACCTCCAACATAAATGAGT
TTAAAGAGGAAGGTGAAGATAGTAAAGGAATTGGAAACTTTGAAGTCGACTTTGATGTGA
ACGAGTTATTCGATTTCTCCACTGAAGGATCCTACGGTTTGGAGTGGGTTAATGAGTTTCTT
GAACTGGGTGAGGAATCTTGGCTCACTGAAAACAAGATGAGCATATAG

>MiMYB33

ATGTCCAATAATTCAGCTTCTTCTTGGAGTAAAGAAGAAGAAAAAGCTTTTGAAAACGCCA
TAGCAATGCATTGGAATAGTAGTACTAGTATTGCTACAAGTGAAGAAGACTCATCATCATCAT
CATCAGAGGAGCAATGGCAAAAGATTGCTTCAATGGTTCCAACTAAAGGTATTGAAGAATT
AAAGCAACATTACCAGTTTTTAATGGAGGATTTGCAGGCTATAGAGGCCGGAAGGGTGCCA
CTACCAGATTATGTAGGAGAATTGGAAGGTGCAACAATGGCAACAACTTCATCAAGTAAAG
ATTTTCATGGATTCTCAGGCTCAGTGGGTGGAGATAAGAGATCAAATTGTGGCTATGGAAGT
GGGTTCACAGGGCTCAGCCATGAGTCCAGCGGGCATGGAGGCAAAGGAGGCTCAAAGTCT
GACCAAGAAAGGAGGAAAGGAATTCCGTGGACGGAAGAAGAGCATAGGTTATTCCTACTT
GGTTTAGACAAATTTGGGAAGGGAGATTGGAGGAGCATTTCAAGAAACTTAGTGATAACAA
GAACCCCAACACAAGTGGCTAGCCATGCACAGAAGTATTTCATACGGTTGAACTCAATGAA

CAGAGACAGGAGGCGGTCGAGCATTCATGATATAACAAGTGTGAACAACAGGGAGGTATC
GTCTCATCATCATCAAGCTTTGATAACAGGGCAACAGGGAAACACTAATCTGGGAGAGGGT
TCGGCAATGGGGGCATCTGGGAAGCACAGAGGGGGTCAGCCACATATGCCTGGATTGGGG
ATATTTGGAGCTCCTGTTGGCCACCCAATTGCAGCTCCTGTGCATATGGCTTCTGCTGTTGG
GACTCCTGTTATGCTTCCCCCTCCAGGCCATCATCCTCATACTTCGTATGTTCTTCCGGTGGC
TTATCCAATGGCACCGCCAACGACAACACACCAATAA

>MiMYB34

ATGACTTTGCAACAGTTCCACAAAAGCTCCTTCTGTGACCAAGACAATGATAGCTCCAATAT
TTATTCTACTTCTTCTATCCCCAATAACTCATCAATGGAGGTGTTTTCCCACATGGGTTCTCTC
CATGCGGCTCCAACTAATACAAATTCTGTGAAAAATAGTCTTAGTAACCTGAAAGATCCTGA
TGGGTTCGAAGAAAACGTTCATAGTTTGAGTAGTGATAGCAATGGTGTGAAAGAAATGACT
GTTAGTGGTGGACAGTCAAAGATTTGTTCAAGAGGGCATTGGAGACCTGCAGAGGACATC
AAACTGAAGGAGCTTGTAGCTCTTTATGGTCCACAAAACTGGAACCTTATAGCTGAGAAGT
TGCAAGGGAGATCAGGAAAAAGTTGCAGACTTAGATGGTTTAACCAGTTGGATCCAAAGAT
CAACAAAGGAGCTTTCAGTGAAGAGGAGGAAGAGAAGCTCATGGCAGCTCATAGAGTTTA
TGGCAACAAATGGGCTATGATTGCGAGGCTTTTCCCTGGAAGAACTGATAATGCTGTTAAA
AATCACTGGCATGTTGTAATGGCAAGGAAATTTAGAGAGCAGTCAAGTGCTTATAGGAGGA
GAAAACTGAGCCAAGTTGTTGTTCATAACACAGCAGTCAGCTTCCTGAATCCATCATCCAA
AAGTTTCAACTTATTTGCACCTCCCATTGATGGAAATGGAGCTGATGATGGTTTTAGTGATCA
TCATCATCAACCTGCTAATCTGATGGATGGTAGAGATTTGTTTCTTGGATCTACAAGCAGCTG
CAACTTCAACTTTCTCCCTCTTCATGATCAAAACCAAACACCTAATTGTGCTTCTTGTGGAA
ATCAGAAAGCTCACATTGATTTGCTTTCTGGTTACAAGCACTGTGATATGGAAAAAGACAG
CTCAAGCTTCAATATTTTCAAGCCTTACACCCTAAATAATAATTTTCCAATGCAACAACCAAA
CCACCATCACCATTCTGCAACTTTCTCAGACACCATGGCATTGGCATCTGCATCTGCATCTG
CATCTTCATCACTATCACTTGCAGACGCCACAGCTACCAGCCATTTTGAGACTAATATATCAC
CACCATTTATAGATTTTCTTGGCACAGGAGCTGCTTCTTGA

>MiMYB35

ATGCAGATCCATAAGGATCCCAAAGCCCCACCAGGAAACGATAATGATGAAATGGAGTGGC
AACGCTGGCAACCAGAAGAAGAGGCCATTTTGAGAGAATACGTGAAGCAATACGGCCCAA
AAGAATGGAACCTCATTTCTCAACGCATGCCAAAGCCTCTCAATCGAGACCCCAAATCCTG
TCTCGAGCGCTGCAAAAACTACCTCAAACCCGGCATCAGAAAGGGCTCTTTAACCCCGGA
AGAACAGTCGTTAGTCATTTCTCTACAGGCTAAGTGCGGCAACAAGTGGAAAAAGATTGCC
GGTGAAGTCCCCGGAAGAACCGCCAAGAGACTCGGCAAGTGGTGGGAAGTTTTCAAGGA
GAAGCAATGGGAACAGTTGCAGAAACAGAGACGGGATTATTCGGATGAAGAAGGGAATAA
TGTTGTTCGAGTAACTTCAGCGTCGGTTTCTTCGCCCGAAAAGGTTGCTCAGGGAAGGTAC
GATCATATTTTAGAGACTTTTGCGGAGAAGTACGTTCAACCGAAGCTGTCATTGCCGGATCC
TGAACCGATTCTTTCACTTGGGTCGGGTCCTTCATCAGCTTCCAGAAATGTGCTTCCTTTATG
GATGAATAGTCATAGTACTTGTTCATCTTTATCATCTTCAGCTTCTTCCACAACCACAGCTTC
TCCTTCTGTGAGTTTATCTCTTTTTCCGTCTGAACCTCCTACTCTTGACCCGGTTGACATGAC
CTGGTTCATACCTGGTCAACAAATGGGCACCTTGATACAGTGGTGTAAGGAGGTTAAAGAA
GGTGAACAAAGCCGGCTGCAACACAAGAAGGAAGCAAAATGGAGACTGAGTAGATTGGA
GCAGCAATTGGAGTCAGAGAAAGCCAGGAAAAGAAGAGAAAAAACGGAGGAAGCCGAG
GCAAAGATTAGATCTTTAAGAGAAGAAGAAATGGCGTTTCTTGGAAAGCTGGAGAGTGAAT
ATAGAGAACAGTCAACTAGTCTGCAGAGAGATGCCGAAAGCAAAGAGGCAAAACTAATGA
AAGCTTGGTGTAATAAACATGTTAAACTGGTGACACTCATTGAGCAGTTTGGTGTTCACAGT
TGCCATGGAAATGGATTCTGTACAGAGAAAGAGGGAAAGAAAGATGCTACGCTAGCTATTC
AAAGGCAAGCCAATGCTACTTCGTACTCTGAAATGACTAATCACTGGGCACTGGGATAG

>MiMYB36

ATGAGTTTACAACGTTTGATGGGTGATGCTCATGGCTTCAGTGTCGTTCACCAAGACTTGAA
CTTTGTGCCTCCTCCTACTTCTCCTCCTCAGCTTTCTCTGTCTGAGTCTTTTGGAGTTTTGGG
TCATCATGCCATGGGAATAGAGACAAATAATCCTGCAATAATGGGGCTTCAGATTTTGAACC
CTTCTGGCCTTAAGCCAGAGGAGAACAGGGGAGCAAAAAAAACAGTTGAAGAAAGAGAC
GGGCTTTTTGGGAGTGAAAAGAAGGGGCTTTCTTTGAACTTAGATGAGGAAATAGAGGAG
GTTAACACCTCTGTCTCTGTCAAAACTAAGCACACTAAGCTTTGTACTAGAGGCCATTGGA
GACCTGCTGAAGATGCTAAACTCAAAGAGCTTGTCGCCCAATATGGCCCTCAAAACTGGAA
CTTAATAGCTGAAAATCTTGAAGGAAGATCAGGGAAGAGTTGCAGATTGAGATGGTTTAAT
CAGCTAGATCCAAGAATCAACCGGAGGACATTCAATGAGGAAGAAGAAGAAAGGCTGTTG
GCTGCTCATAGATTTTATGGAAACAAATGGGCGTTAATTGCAAGGCTATTTCCTGGGAGAAC
TGATAATGCAGTGAAGAATCATTGGCATGTGATAATGGCTAGAAAACTGAGAGAACAATCA
AGCATTTACAGAAGGAGAAAGCCAAATTCTGCCTCTCAAACTGGACTTGAAAACATGATTA
TCAACAAAAATGCCACCAGTGAATCAACCAACTCCAGCAATGAATCCGCCTCAACCATCAC
TGATCTCTCTCTCACTCCATCTTCAACCAAATTTTCACCTGCTATTTTCACTAGGTTCTCACT
CATGGGTTCATCTAAAGAAAAGGAGATAACAAGGGGAAGTATTGGAGATGTTGACAAATTT
TATGGATATTACAAAGCAGGTAAAGTGGAAGTGGTTATGGGAGTGGACCAATCTGTGCATTC
ATCAGATTCAAATTCAGAAGTTTCAGCAACAGACTCTGTAACCAACAACAACACCAACACC
AACACCAATCTATCTCTCGCAGGGCAAAATACTTCTCAGAAGAACATTAGCATGGCCTTCAT
TGATTTTCTTGGAGTAGGAGCTTCTTAG

>MiMYB37

ATGAAGTGGGAAACGGAGGTAATATCCCCTTCACCGTATCTTTCAACGAACAATTGGGTTCC
TGAAGATGGCCAGACCACAAAATGGACAGCCCAAGAGAACAAAATGTTTGAGAATGCTTT
AGCTATATATGATAAGGATGCTCCTGATCGATGGCACAAAGTTGCAGCAATGATCCCGGGGA
AGACAGCAGTAGATGTAATTAAGCAGTATAAAGAATTGGAGGCTGATGTGAGCAATATAGA
GGCAGGACTGATTCCAATTCCAGGATATAATAATACCACTTCCTCTCCATTCACATTGGATTG
GGTTAACAATCATAATAGCTATGATGGGTTCAAGCAATCATATGGTATTGGTGGAAAGAGATC
CTCTCTGGTTCGCCCTGCTGAGCAGGAAAGGAAGAAAGGAGTTCCATGGACAGAAGAGGA
GCATAAACTGTTTCTGTTGGGATTGAAAAAGCATGGCAAAGGAGATTGGAGAAACATCTCA
CGTAATTTTGTCACCAGTAGAACGCCAACCCAAGTGGCCAGTCATGCTCAAAAGTATTTCAT
CAGACAGCTGTCTGGAGGTAAAGATAAGAGGAGAGCTAGCATTCATGACATAACAACTGTG
AATCTCAACGACACAAGAACTCCTTCCCCAGACAATCAAAGCTCTCTTTCACCAGATCAAT
CCACAGGGCTTTCTCAGCAACCTGGTTCTGGTGGCTTGTCCAGAACACAATTTCAGTGGCA
TCAGCCAAATCATGGATCAACCACATCCTTTAGTTCAACACAAGGAAGTATGTTAATGACAT
CCCCTTATGGGATGAACTCGTATGGGCTAAAAATGCCAGGACACAACCTGCAAAGAAGTGC
AGTGCATGAGCCTTTCTTTGGACTTCAAAATCTGGCTTTTCAGATGCAATACCCTCATGGAT
AA

>MiMYB38

ATGAGGATTATGATAAAGGGAGGCGTGTGGAAGAACACGGAGGATGAGATCCTTAAAGCTG
CCGTTATGAAATATGGTAAAAACCAGTGGGCCCGTATCTCCTCCCTCTTGGTTCGTAAATCC
GCAAAACAGTGCAAGGCTCGCTGGTACGAGTGGCTCGATCCCTCCATCAAAAAAACTGAA
TGGACACGAGAAGAGGATGAGAAGTTACTTCATCTTGCTAAGCTTATGCCTACTCAGTGGA
GAACAATTGCCCCAATTGTTGGCAGAACTCCATCTCAGTGCCTTGAAAGATATGAGAAACT
CCTTGATGCAGCTTGTGCCAAAGATGAGAACTATGAACCAGGTGACGATCCACGGAAATTG
CGTCCTGGAGAGATTGACCCAAACCCAGAATCAAAGCCTGCTCGTCCAGATCCAGTTGATA
TGGATGAAGATGAGAAGGAAATGCTTTCTGAAGCGCGAGCTCGTTTAGCTAACACTAGGGG
TAAAAAGGCAAAAAGAAAGGCTAGGGAGAAACAGCTTGAAGAGGCTAGGAGGCTTGCTT
CTTTGCAAAAAAGGAGAGAACTGAAAGCTGCCGGAATTGATACTAGGCAAAGGAAAAGGA
AAAGGAGGGGAATTGATTATAATGCAGAAATTCCTTTTGAGAAAAAGCCTCCTCCTGGCTTT
TTTGATGTTACTGATGAAGACAGGCCTGTAGAACAAGTTAAATTCCCAACGACCATTGAAG
AACTTGAAGGGAAGAGGAGGATTGATGTAGAAGCTCAGTTAAGAAGGCAAGATATTGCAA
AGAACAAAATTGCACAAAGGCAGGATGCTCCTTTGGCCATACTGCAGGCAAACAAGATGA
ATGATCCAGAATCAGTTAGGAAGAGGTCAAAACTCATGCTTCCTGCACCTCAGATTTCAGA

CCATGAATTGGAGGAAATTGCAAAAATGGGTTATGCTAGTGATCTTCTTGCTGGGAACGAG
GATCTGTCAGAAGGCAGTGGCGCAACTCGTGCTCTTCTTGCAAATTATGCACAGACACCGC
AGCAGGGAATGACACCATTACGAACCCCACAAAGAACACCAGCTGGTAAGGGTGATGCTA
TTATGATGGAGGCTGAAAATCTGGCCAGGTTGAGAGAGTCACAGACACCATTACTAGGAGG
AGAGAATCCAGAGTTGCACCCTTCAGATTTTTCTGGGGTCACTCCTAAGAAAAGGGAAATC
CAAACACCTAACCCTATGCTGACTCCTTCCGCAACGCCTGGTGGTGTGGGACTTACTCCCA
GGATTGGCATGACACCTTCAAGGGATGGCTATTCTTTTGGTGTGACCCCTAAAGGAACTCCC
ATAAGGGATGAACTCCACATTAATGAAGAAATGGATATGCATGATAGTGCAAAACTTGAACA
GAGAAGACAAGCTGATCTGAGAAGAAACTTACGCTCTGGTTTGAGTAATCTCCCACAGCCC
AAGAATGAGTACCAAATAGTAATCCAGCCACCTGCAGAAGATAGTGAAGAACCAGAAGAA
AAGATTGAAGAAGACATGTCTGATAGGTTAGCCAGAGAAAGGGCTGAGGAAGAAGCAAGG
CAGCAGGCATTACTTCGGAAGAGATCAAAAGTGCTGCAGAGAGAGCTTCCTCGGCCACCA
GTTGCTTCATTGGAACTAATTAGAAATTCTTTGTTGAGGGCTGACGGAGACAAGAGTTCCTT
CGTTCCTCCCATGTCTATTGAGCAGGCTGATGAAATGATAAGAAAGGAACTTCTAACACTAC
TAGAGCATGATAATGCTAAATACCCCCTTGAAAAAGTGGAGAAGAAAAAGAAGGGCTCCA
AGCGTTCTGCTAATGGATCTGCTGCTCCTATACCTGTGATCGAAGATTTTGAAGAAGAAGAG
CTGAAGGAGGCTGATAATTTGATAAAGGAAGAGACTCAATATCTTCGTGTGGCAATGGGGC
ACGAAAATGAATCCCTCGATGAATTTGTGGAAGCACACAATACCTGTTTAAATGATCTCATG
TACTTCCCCACCCGAAATGCTTATGGTCTCTCAAGTGTAGCTGGGAGCATGGACAAACTTGC
AGCCTTGCAGAGTGAATTTGAGGTTGTGAAGAGGAAGATGGATGATGATAAGGAGAAGGC
ATTGCACCTTGAGAAAAAAGTCAAACTTCTCACACAAGGTTATGAGAAACGGGCTGAAAA
TCTTCGAAACCAAATACAGTCAACTGTCAAACAGATGGAAACTGCTGGAACAGAACTAGA
GTGTTTCCAAGCTTTGCAGAAGCAAGAGCAACTGGCAGCATCAAACAGAATAAATGGTCT
GTGGGAAGAAGTTCAGAAGCAAAAAAAGCTTGAGCAAACATTACAAAAGCGCTATGGTGA
TGTCTTGGCTGAGCTGGAGAGACTAAGCCAACGCATAGATGAATACAGGGTACAGGCACAA
AAACAAGAAGAAATTGCTGCAGAGAAGCTTGCACTTAAGGCAGCAGAAGAAAATCACATA
ATTGAGCAAAATTCAGGAGCCTCCGAAGCTCTACGTTCAGAGAAACTTGGAAGCTCTGTGC
CTGCTGAGCAATCTCATGATGAAAATCCTGGCCCACAAATTGATGCTGTTCACATGGACGTT
GATTCAGGGAAAGATCATATAACAATTGATGTAGATGTGAGGCAAAATGTTGTGGAGGCCA
ATCCAGATGCGGTTGCCCCGGAAACTGTAATACATAGAGGCAGTTCTGCTAATGAGGATGTC
ATGGAAGTTCCCAGTGCTGAAGTTGATAATGCATCCTTGGCATCCAAAGAGGCAGAGGCCA
ATGATAATCTGTCTATCTTGAATGGAGACTCTACCAACAAACAAACTGGAGAAGATGTTGCT
ATTTCCGAACCGGTAAACACAGAATTGGATGGGAAACAAGACAATCAAGAGAATACGGTTA
TATTAGCCGATGATGATTAA

>MiMYB39

ATGGAGCTTGAAACTGGTACAAACCTTAAACAAAATCATGCTCCATTTTTCTTCTCTCATGA
AAATTACATTAAATCCTTCATAAAATCTGAGATTCTTTTCGATGAAGGATCTTCTTCCAAAGG
TTACCTTCAAGATTTTCATCATCTTAGCCATCACTTTAATGCTAATGGGTGTTCCTCAAATCCT
ATGTTTGGAGTCCACATTCCATGTTTCGACTCTTTGGATGTCTCTGCACATGCTTACTCATCG
TTAAACTTCGATTTTCATGAGTATAAATTTCAAAGTGGAGGATACTTGGAGAATCATCACCA
AATTATGAATCCCCTGGTTGATATTACAGGATCCAATCAGAGCCACATGCCATTAAGTTTCAT
AGAGCCTGTGAATTTTGTTGTATCAGATGAAGTCTCATCTGTAAGTGCTGATAATATTAGTAG
TTACCATAAGAAAGCTGGCATGAATAAGAAGAACAGGCCATATCTTTCGACAAGGACAGGG
AAAGTTGGGAAGAAGTACAATGTAGTGAAGGGGCAATGGACAATTGATGAAGATAGGCTC
TTAATTCGACTGGTTGAACAACATGGCATGAAAAAATGGTCTCATATAGCACAGATGTTGCC
TGGGAGAATCGGAAGCAGTGTAGAGAGAGATGGCATAACCATCTGCGGCCTGATATCAAGT
TGGAGCGAAGAAGAGGACAAGGCACTGATAGAGGCACATGCAGAGATAGGCAACAAATG
GGCAGAGATCGCAAAGAGATTGCCTGGAAGAACAGAAAACTCTATCAAAAACCATTGGAA
TGCAACAAAGAGAAGGCAGTTCTCTAAGAGAAAATGTCGTTCCAAAAACCAGAGAGCCTC
AGTCCTGCAAGACTATATTAAGACCTTGAATTTAACTTCAACCGCCACCAAACATCGGACAA
AAACTTCCCTCAAGGTTAATAACAAAACCAAAATATTATCAAACCAGGCACCTGTTGATAAT
GACGAGGTCTCCCCGTGCGATCACTTGGTCCTTGATTTTGATTTAGTGAAGTCCCGGATTT
TGATTTTGATGATGATAAGTTATTCGAAGATGACTGTAGCCTTGATTCTCTTATTGAGCAGAT
GCCTTGCGGTTCTGCTGTTGGTGATGATGATAAGAAAAGCTTTGAAATGGAGTTGTCACCA
CTAGATGAGAAATCTCAAGTGGGGGACATGGATTTGGTGGAGATGATTTGTCAAGCTAATAA
TATGTAA

>MiMYB40

ATGAGTCACACAACAAGTGATAGTGATGATAGGGTGCTTTCCAGAGGTCAGACTGATTTGC
CGTTAGTGGATGAAGGTGGCAATTCTGTTCAAGGAGTTATACTGAAGAAAGGACCATGGAC
ATCTGCTGAAGATGCAATTTTAATTGACTATGTTAAGAAGCATGGGGAAGGGAACTGGAAT
GCTGTTCAGAAGAACTCAGGGCTGTTCCGTTGTGGCAAAAGCTGCCGATTGAGATGGGCA
AATCACCTGAGGCCAAACTTGAAGAAGGGCACATTTACTCAAGAAGAAGAGCAGCTGATT
GTTGAGCTCCATGCCAAAATGGGAAACAAGTGGGCACGCATGGCTGCACATTTGCCGGGTC
GCACAGATAATGAGATAAAGAATTACTGGAATACTCGAATTAAACGACGTCAACGTGCTGG
CTTGCCTCTCTATCCTCCAGAAGTGTCTTTCCAAGCATTGGAGGAGAGTCAAAACCAAAAC
ATTGGTGGAATCAATAATGGGGATAAAGGTCATCATGATATCTTGCAGGCTAATGGTTATGAG
ATACCTGATGTTGTATTTGACAGTTTAAAAGGCAATCAGGGTGTCTTACCTTATGTTGCTGAA
CTTCCTGATATTTCCTGTAGCAACATGCTGATGAAAGGTCTGGGCTCTCCATTCTGTAGCTTC
ATGCCACCAACAATTCATCGCGAGAAGCGTCTTCGAGAATCTGTGGGATTATTCTCTGGTTA
TAGTGAAAGAGCAAAGAACGAGTTCTGCTCATTTGATCAGTTCCAGAATGATACTCCTGATA
AGATGGCTCCACCATTTGGGCTGCCTTTTCCTCTGGATCAAGATCCTACAACCAAAGCCCCT
GAGACTTTTGGGGTAATACAGGGTGGCCAGACCCTTTCAAATGGCAATTTCTCTGCTTCTAC
GCCCACTTCAGGGGCTGTGAAGTTGGAGCTCCCTTCACTCCAATATCCAGAAACTGATTTA
AGTAGCTGGGGTACATCATCTCCCCCACAACCTTTACTTGAAACAGTTGACTCTTTTATTCA
GTCTCCTCCAACTGGTACTGTTGAGTCAGATTGCCCTTCACCGCGCAACAGTGGCCTGCTT
GATGCTTTACTCCATGAGGCAAAAACTTTGAGCAGTGCAAAGAATCATTCCTCTGACAAGA
GTTCAAATTCATCTTCTGTTACTCCTGATATAGCTGATAGTTGCCTGCCGAATGTTTGTCAAA
ATGAATGGGAAGACTACTGTGACCCCCTTTCTCCACTGGGTCATTCTGCAACCTCTCTCTTC
AATGAGTGCAGTCCACTCAGTACCAGTGGAAGTTCATTGGATGAACAAGCTCCCATTGAGC
CATATGTTGGGTGCAAGGTGAAATTAGAACAGGTTGACCAGGCATGGACTCCTGATAGAGG
AAAAGAAAGCAGTAACCTGTATGATCTTAGTCAGGCTGATGCTTTACTTAGTTCAGACTGGC
TTGAGCAGGGGTCTGCTTATGTTAAGAACCCAACTGTGATTACTGATGCCATAGCAACCTTT
TTTGGAGATGATTTGGGCAGTGAATATAAGCAGATGACAACAGGAACTTCGACAGCAAGTC
AAGGATGGGGCTTTGGTTCTTCTGCATGGAATAACATGCCGGCAGTGTGTCAAATGTCTGA
ACTCCCTTGA

>MiMYB41

ATGCCAGCATTAATATCGGACAAAAAGGAGAAGATGGTGTCTAGGTTTATCTCTAGTAATGG
ATTGGTTGAAGATCCATGTGCTGTGGAGAAGGAAAGAGCTATGATCAATCCCTGGACTGTG
GAAGAGAGAGAAATTTTCATGGATAAATTAGCCACCTTTGGGAAAGATTTAGGAAAATTG
CTTCATTTCTTGATTACAAGACAACTGCTGATTGTGTTGAGTTCTACTATAAAAACCACAAG
TCTGATTGTTTTGAGAAAATAAAGAAAAGGCCTGTACCAGTGAAGCCGTGTTCTAATACGT
ACTTTATGAGACCAGGCAAAAAATGGGATCGGCAGATGAATGCAGCATCCCTTGACATTTT
GGGTGAAGCTACTGAGATGGCAGCTGCATTCCAAGCTGACAATGTCCGACAGATTTCTGGT
AGAATCTCTTTGGGAGGGTGCAGTGATTCAAAATTATCTTTTGGTGATGATGGCATGACAGA
AAGGTCAAGCAGTTTTGATGTACATGGGAATGAGAGAGAAACAGCTGCTGCAGATGTTTTA
GCGGGAATATGTGGTTCCTTGTCATCTGAGGCTATGAGTTCTTGTATCACAAGCTCTGTTGAT
CCTGGAGAGGGCCAACAGGAGTGGAAGCACCAGAAAATGGATTCTTTTAGGAGGCATTCA
ACTTCTGATGTTACTCAGAATGTTGATGATGACACTTGCTCTGATGAGAGTTGTGGGGAAAT
GGATCCTGCTGACTGGACAGATGAGGAGAAATCCATCTTTATACAGGCAGTGTCATCTTATG
GTAAGGATTTTATCATGATCTCACGATGTGTTAGAACGAGGTCTAGCGATCAGTGCAAGGTT
TTTTTTAGCAAGGCACGGAAGTGCCTTGGACTGGATTTAATACATACTGGACGTGGAACTGC
AGGAACCTGTGTAAGTGATGAGGCCAATGGTGGAGGGAGTGACACAGAAGATGCATGCGT
CTTGGAGACTAGCTCAGTTATCTCTAATGATAAGATGGACTCTAAAGTGGATGACTTGCCAT
CCTCTGGGGTCAACAAGACTCGGGACAAATCTGATTTGGGAACCATGAACTTGCCCACTGA
CCTGAATAAATCAGGGGATGAAAATGGAGAGAGGCTTCTTGATGATCAAGATTCTAAGGAT
GTCAGTCCTTTGGTTTCTGATGACTGTAAGAGACTGCATAATCCTGAGGTTTATTCTGAATTT
GAAAGAATGAGAATGGATAATGTTGATTGCCAGTCCGAGTCACTGCAGGCCCACAAAATTC
CTGTTGAATTTGTTGAGAAGGAAGGAGGAAAAGATAAATTAGCAGAACAAGCTGTTGGTCT
GCCGGTACCACTCACAAGTAGAGAAGCCATGAAATTGTGTTCGTCAGGCTTAAATGTTGTTA
TTGAGACTACTGAGGCTCCTGTTAAGGGATATGAAAATGGTTTGGAGGATAGAATTGAGCCT
AATAAAACATGCAAAGCTGATGAACATGGTGGCAGGGGTATGGTGCAAGGTTCAAATGTAT

TTGGGAATATTGTTGATCTGACTGTAGATACAAATTCTTGTTCTGTTCCTTCGAAGTCAGATA
GTGGGGATAAGCTTCCTGTTATGTCATTGCCACTGGAGAATTCTCTTGCTTCTGTAAGTTCTG
TGCCACAAGTTTCTGCTGCCAGTCAATGTGAAAAATCAGTTGAGCAAAATAGGCTGTCCTC
TACACTTGACTTTCAGGGGAATAAAGATAAAAGTGCTTGTATGTCTGTTGGCAATGAAGACT
ATCACCGTAATTTGTTGGGGCATTCTCTACTGAACCATGTTGAACACTCTCAGATTCTCAAG
GGTTATCCGTTGCAGATATCCTCAAAGAAAGAGATGAATGGCGATATTAGTTGCAGACAACT
TTCTGAGGTTCAAGGCATTTCTAAGTCAGACAGGAGTGATGGTGTGCCCTACATGGCTCAG
GATTGCTATATTCAGAAGTGTAGTAGTTCGATGTCTCATGCTTCAGTTCCGGAGCTTCCATTT
CTCGCTTCAAACTTGGACCAAAAGAATGATCCTCCAAGATCCCATTCACGGAGTTTGTCTG
ACACGGACAGACCACGCAAGAATGGTGATGTTAAATTGTTTGGCAAGATACTTAGTCGTCC
CTCGTCTTCACAGAAGCCAAATTCCAGTAGTCGGGAGAATGAAGAGAAGGGAGCCCATCA
TCATAAACAAAGCTGCAAGGTATCAAATTTGAAATTCACTGCTTCTCATCCAGCTGAGATGT
TGAAGTTTGATCGAAATAACTATCTAGGTCTTGAGAATGTTCCTATGAGAAGTTATGGATTTT
GGGATGGGAACAGAATTCAGACTGGTTATTCATCTTTGCCTGATTCCGCCATTTTGTTGGCC
AAGTATCCTACAGCCTTTGGCAATTATCCTGGCTCCTCATCCAAAATGGAGCATCAGGCTTT
GCAAGTCAAGAGTAGTGAGTGCAATTTGAAGGGTGTAGCAGTTCTTCCGCAAAGGGAAATT
AGTAGCAGTAATGGAGTGGTGGACTATCAGGTGTATAGGAGTAGTGATGTTACTAAAGTACA
GCCATTTGCCATAGATGTAAAGCAGAGGCAGGACTTGTTATTCTCCGAGATGCAGAGACGA
AATGCATTCGAAGCACTCTCAACTCTCCCACAGCCAGGAAGAGGGATGGTCGGAGTGAAT
GTTGTAGGACGAGGAGGTATACTTGTAGGTGGACCATGCACGGGCATTTCAGATCCAGTGG
CAGCGATTAAAAGGCACTATGCTAAAGCTGACCAGTATGGTGGGCAGAGCGGGAGCATTAT
TCGGGAAGAAGAATCCTGGAGAAGTAACGGGGACATAGGCAGGTCAGTTGACAGTTTCTC
AGCTCTTTTTGCCCGCTTTTATTCCTTTATTTAG

>MiMYB42

ATGGAAACTCTGTATCCAGCTTTGTATATGTCGGATGCAAGTTGGTTTCTTCAGGAGAGCCA
GAGCACAGGCTGGACTAAAGAAGAGAACAAGAAGTTTGAGAGTGCTCTTGCAATTTACAG
TGAGGGCACGCCGGATAGATGGATTAAAGTGGCGGCTCTAATTCCAGGAAAGACGGTGCTT
GATGTGATGAAACAGTACAAAGTATTGGAAGAAGATGTGAACGATATAGAAGCTGGAAAGG
TTCCGATTCCAGGTTATTATAGCTCTTCCTTTGCATTAGAGTTGGTTTCAGAACGTGATTTTG
ATGCTAATAGGAAGAGGTCATTGGTGAAAAGTTCAGATCAGGAGAGGAAGAAGGGTGTGC
CTTGGACTGAAGAAGAGCACAGGCTTTTTCTGAAGGGACTTCTTCAGTATGGTAAAGGAGA
CTGGAGAAACATCTCACGGCATTTTGTAATTACTAAGACTCCTACTCAAGTGGCAAGCCATG
CTCAGAAATACTTTATAAGGCAGCTTTCAGGAGGGAAAGACAAGAAGAGACCAAGTATCC
ACGACATCACCACTGTCAATCTAGCCGATACAAATTTACCAGACAATCAAAAACCTTGCTCT
GTTGACCAATCCAATGTGCTTCCACAGCAACAGAAGTCCTCTAGCTTGCCGAAAGTAGGAC
TTGAGTGGAATGACTCAAACAATGGAGTAGTGATTTTCAATCCATCTAATGGTAACAACCTG
TTTGTGCCGTCTGTTAATGATATTGGTTCAAATGGCCTTAAACTTCAGGGGAAAAATTTGTAT
GGCACTACTTATCATGGAGTTCATTTCAAACCCCGAAATTCAGTGTTCTGA

>MiMYB43

ATGAGTCAAACAACAAGTGATAGTGATGATAGGGTGCTCTCCAGGGACCAGACTGATTCGC
CGTTAATGGATGAAGGTGGCAATTCCGTTCAAGGAGTTATACTAAAAAAAGGACCGTGGAC
ATCTGCTGAAGATGCAATTTTAATTGACTATGTTAAGAAGCATGGAGAGGGGAACTGGAAT
GCTGTCCAGAAGAACTCAGGGCTGTTCCGTTGTGGCAAAAGCTGCCGACTGAGATGGGCC
AATCACCTGAGGCCAAACTTGAAGAAGGGGGCATTTACTCAAGAAGAAGAGCAGCTGATT
GTTGAGCTCCATGCCAAAATGGGAAACAAATGGGCACGCATGGCTGCACATTTGCCTGGTC
GTACAGATAATGAGATAAAGAATTACTGGAATACCCGAATTAAACGACGCCAACGTGCTGG
TTTACCTCTTTATCCTCCTGAAGTGTCTTTCCAAGCATTGCAGGAGAGTCAAAACCAAAACA
TTGGTGGAATCAATAATGGGGATAAAGGCCATCATGATATCTTGCAGGCTAATGGTTATGAGA
TACCTGATGTTGTGTTTGACAGTTTAAAAGCCAATCAGAGTGTCTTACCTTATGTCCCTGAA
CTTCCTGATATTTCTGGAAGCAACATGCTAATGAAAGGTCTGGGCTCTCCATTCTGTAGCTT
CATGCCGCCAACAATTCATCGCCAGAAGCGTCTTCGAGATTCAGTGGAATTATTCTCTGGTT
ATAGTGGAAGAGTAAAAAATGAGTTCCCCTCATTTGATCAGTTCCAGAATGATTCTCCTGAT

AAGATAGCTCAAACATTTGGGCTGTCTTTTCCACTTGATTCAGATCCTACGACCAAGGCCCC
TGAGTCTTTTGGGGTAATACAGGGTAGCCAGACCCTTTCAAATGGCAATTTCTCTGCTTCTA
AGCCCACTTCAGGGGCTGTGAAGTTGGAGCTCCCTTCACTCCAATATCCAGAAACTGATTT
AAGCAGCTGGGGTGCATCGTCTCCCCCGCAACCTTTACTTGAGACAGTTGACTCTTTTATTC
AGTCTCCTCCACCAACTGGAACTGTTGAGTCAGATTGCCCTTCACCACGCAACAGTGGCCT
GCTTGATGCTCTACTCTATGAGGCAAAAACTTTGAGCAGTGCAAAGAATCATTCCTCTGACA
AGAGTTCAAATTCTTCTTCTGTTTCTCCTGCTGATATAGCTGATAGTTCCACCTTGAATATTTG
TGAAACAGAATGGGAAGACTATGGTGACCGCCTTTCTCCACTGGGTCATTCTGCAACCTCT
TTCTTCAATGAGTGCACTCCTCTCAGTACCAGTGGAAGTTCCTTGGATGAACAGGCTCCTGT
TGAGCCATATGTTGGGTGCAAGGTGAAATCAGAACCAGTTGACCAGACATGGACTCCTGAT
AGAGGAAAAGTAAGCACTGATCTGTATGATATTACTCAGTCTGATGATTTACTTAGTTCAGAT
TGGCTTGAGCAGGGTTCTTCTTATGCTAAGAACCCAACTGTGATGAATGATGCCATAGCAAC
CCTTTTTGGAGATGATTTGGGCAATGAATATAAGCAGATGACAACAGGAACTTCGACAGTG
AGTCAAGGGTGGGGTTTTGGTTCTTGTGCATGGAATAACATGCCGCCGGTGTGTCAAATGT
CTGAACTGCCATGA

>MiMYB44

ATGTATCAAGACCCCAACGCCTTTCACTCGTCTTCGTCAACTCACTGGAACCGACTCGAGG
ACAAGCTCTTTGAACATGCCCTTGTTTTGTTCCCTGATGAAGTTCCCGATCGATGGCAGAGG
ATAGCCGATCAAATTCCCGGGAAGTCTCCCCGCGATGTCAAGGAACACTATGAGGTGCTCG
TTCATGACGTCACTGAGATTGACTCGGGCCGAGTTGAGTTGCCTTGTTATGCTGATGAGTCA
GACGATTGGGACTCCGCCGGTCAGATCTCGTTTGGGTCAAAGCCTGTTAAGCAGGGCGAGC
CTGAGAGGAAGAAGGGTACTCCCTGGACGGAAGAAGAACACAAGTTATTTCTGATTGGATT
GAGTAAATTTGGCAAGGGTGATTGGAGGAGTATTTCAAGGAATGTAGTGGTGTCAAGAACA
CCAACCCAGGTGGCTAGTCATGCCCAGAAGTATTTCCTTCGCCAAACTAACATGAAGAAAG
AGCGAAAAAGGGCGAGCATCCACGACATTACCACAGTGGATAGCAAGCCAATTCCACTGG
CTGTAGACCAGAACTCAATGGGTCTGGCTGTAGACCAGAACTCAATGGGTCTGCCTGTTGA
CCAGAACTGGAATCCTCCCCCTGCAGTTTCAGGTCACCAACAGCCACCTACATATCAACAG
TTTCCTCCACCAAATCAGTTTCTCAGTCAAGGAGGGTCAATGGGGTATCAAAACTATGGCTT
TCCCATGTAA

>MiMYB45

ATGAAGAAGAGTGGAGGTGCCAATGGAGAATCCAAGAAGAAGGAACGGCATATTGTTACG
TGGAGTCAACAGGAAGATGATATACTGCGGGAGCAGATTAGCAAACATGGAACAGACAATT
GGTCAATTATTGCATCAAAATTTAAAGATAAAACAACCAGACAGTGTAGAAGAAGATGGTA
CACATACTTGAATTCGGATTTCAAGAAAGGGGGATGGTCACCTGAGGAAGATATGCTGTTAT
GTGAGGCCCAGAAGATATTTGGTAATAGATGGACAGAAATAGCCAAGGTGGTTTCTGGGAG
AACGGATAATGCTGTCAAGAACCGGTTTTCTACATTGTGTAAGAAAAGAGCAAAATATGAA
GCCTTAGCTAAAGAGAATAACAATTCATACATGAACACAAACAAGAAGAGAATTTTATTCC
AAAACGCGTTCAATGCAGATGGAACACCAGAAAATACAGCGCCAGTTAAGAGAGTTAGAA
GGTCCCACATCCCTGATCTTGCACAAAGCTGCAACATTATAAACAGATCACATAGGCAGTCT
GGAACAACAATGAATCCGCAGTTAAGACCTCCATTTACAGTGTTGGTTCAAAACTTACCCA
GTGTCAACAGCTTGCCAGCCCAGCATCAGCATCGTGATATCAAGGAGATTTCAAATAATGCA
CCTCAGAACAATAAAACACAAGTAATGTTTCTTAAGAAGGATGATCCAAAGATAACTGCTTT
GATGCAACAAGCGGAACTTCTTAGCTCACTTGCACTGAAAGTTAATACAGAGAACACAGA
ACAGAGTTTGGAAAATGCTTGGAAGGTTGTTCAAGATTACCTGAACCGAAGCAAAGAAAA
TGATATCCTAAGATATACAATCTCTGATATCGGTTTTCAGTTCGAAAATTTTAAAGACTTGATA
GAGGACTTAAGGAGCAGTAATGAGGGAAGTCATCCATCCTGGAGGCAACCAGATCTATATG
AGGACTCTCCAGCTAGCTCTGAATATAGTACTGGATCAACTCTAATTCCTCATCTAGCCAGTG
AAAAACCAGATCAAATTCAAGCTGAGGTTGACATACTGCATCAGAATATTGGAGCTGAATT
ACAATTGATGAATAATGGAGAGCAACATTGCTTGGAGGAACAAAATAAAGGGCATGCAAAG
ACAAATGAAGTGGAGTTATTCCCCTCTGGTGGTGAAATCACAAACAACAATGGAATTGTTT
CTGTCTCATCAATTACAGAGTTCAGTTCCCCCATCCAGGTTACCCCACTATTCAGATCCTTAG
CAGCAGGAATCCCCAGCCCAAAATTCTCAGAAAGCGAGCGGAATTTCCTTCTAAGAACACT
TGGAATAGATTCCCCTTCTCCCAATCCAAGCACCAATCCTGTACAGCCACCTCCTTGCAAAA
GAGCCCTTCTTCAAAGTCTATAA

>MiMYB46

ATGATTTATGCAGATATTGCTTCTCTGTTTCTTAGTTCCACTCATCTAAGGGCAGGTTCAGTT
GCTTCTGCTTCTCTAGAGATTTATTTTTGCAGGAACTCAGATAGGGAAAATGGAAGAGGTAG
CCGGAATTTTCCTCCCATTGGCAACAACTCAACCAGAAGCTTTGAGGGACCACATAATATTG
AAGTTAATGTTGATGGTGAGGGCTCTAATTACAGAGATGGGTTTGGAGAAAACGAGACCGT
AATCCATAATGTCAACTTGAATGAAGAGTTTAACCATAACGAGACTATAGAGATGAGCGGG
AAGGAAACAGATAAGGGGCTGGCAAAGCTTTGTGCTAGAGGCCACTGGAGGCCTGCGGAA
GATACCAAGCTCAAGGAACTTGTAGCGCTTTATGGTCCCCAAAACTGGAACCTTATAGCCG
AGAAGTTAGAAGGTAGATCGGGCAAAAGCTGTAGGTTGAGGTGGTTTAACCAGTTGGATCC
AAGGATCAACAGAAGTGCTTTTACTGAAGAAGAAGAAGAAAGGTTAATGCAAGCTCATAG
ACTTTATGGCAACAAATGGGCCATGATAGCCAGGCTCTTTCCTGGAAGAACTGATAACGCA
GTCAAGAACCATTGGCATGTTATAATGGCTAGGAAGTATAGAAAACAATCCAGTGCGTATAG
AAGGCGGAAGCTGAGCCAGTCTGTCTACAAAAGAATGGAGCATATCCAGGGGATAATATGC
AGAGGTACAGCCGCCAGAACTGAACTGCAACCATATTGTTTTAGTGTCCCCAATGGCGGAT
TCAGCAACATCTCTCCCTTCTCTTATGGAACTTATAATGGTGGTGGTGGCGGGGTTAATTTTA
GCGTCAACGGTTCACCCCAGATGACCAGTGGGCAACAAACAACCTTAAGTAACAAGGAAC
CTTACAGTCCCAAGAGCAAAGATATTATGGGCATGTTCAGCCAGGTGAGGTCTTGCGATGG
GCCAATTGATGAAGGCCACGTGTCTGGCATCAATCTTCTCAACCATCGTCAGTACTTGATGG
CAATGCAACAGTCAAGCTTCCAACAGCACCACCCTCACAGTTTCTTTGATGCTATGGCATCT
ACACCTCAAGTTTCAGCCAGTGAAGCTTCATCATCGGTAGGCAGTCATTTTGATACCGTTCC
ACCACGCTTCTTTGATTTTCTCGGGGTAGGAGCCACGTGA

>MiMYB47

ATGAATCGGGTTTTAGAGACCGATCGGATCAAGGGGCCGTGGAGTCCTGAAGAGGACCAG
CTGTTGCACCAACTCGTGCAATGTAACGGCGCGAGGAACTGGTCCCTTATATCCAAATCGAT
TCCGGGCCGGTCCGGGAAATCTTGCCGGCTGCGGTGGTGCAATCAGCTATCCCCCGAGGTC
CAGCACCGTCCCTTCACCTCCGACGAGGACGAGATCATCATGCAAGCGCATGCTACGTTCG
GCAACAAGTGGGCGACCATAGCGAGACTCTTGAACGGCCGTACGGATAACTCCGTAAAAA
ACCACTGGAACTCAACCTTGAAACGGAAGTACGCGATGATAACCACGGAGGATGATAACG
ACGATATCAACGAAAGAGGAAGAGATAGTTTTTATCCAGAGAAGAAAAGATCTGCGCCTGG
TTCGTTTTCTGGTAATTCATCGAGTCCGTCTGGATCCGACGTGAGCGATTCTGGTCTACCGG
CGATGTCTAGTTCGGATATGTGTGGGTCTGTTGGGACGTTTGTTGAAACCGAGAATGGAAG
CGATAAAAACATCAATAATAATGGTGATGTTGCGTTCAATGTATCGACTGAACTTACTTTGAA
CTTACCTGGTAATGAATCGAACGATTTGACTCAGTGCTGGGTACCAAGGGAGGTTTCACAG
AAGGAAGAGAGTAGCACTGATTACAATAATAAAACGAGGGTAGTTTGGCCGGAGTTGATGG
CTTTGATGCAAGAAATGATAAGGAAAGAAGTGAGGAATTATATGGAAGAGGCAGTGACTAA
TAATGGGATTAAGGGTTACTAA

>MiMYB48

ATGACAGTGGATGAAGTAGGCAGTAGCTCTGTATGGACTAAAGAGCAGGATAAAGCTTTTG
AGAATGCTTTAGCAACTTATCCTGAAGATGCTTCTGACCGATGGGAGAAAATTGTATCTGAT
ATACCTGGGAAAACACTAGATGAGATTAAACATCACTATGAGCTTTTGGTGGATGATGTTAA
CGACATTGAATCTGGTTGTGTTCCTGTGCCTAGCTATAATTCATCATCTGATGGATCTACAGG
CCATGGTGGTGATGAAGGAACTGGAAAGAAAGATAGCCACTATGGGCATTATAACAATGAG
TCCAACCATGGAAGCAAGTCTTCAAGGTCAGATCAGGAACGCCGTAAAGGGATTGCTTGG
ACAGAGGATGAGCACAGGTTATTTCTTCTTGGTTTGGACAAATATGGTAAAGGTGACTGGC
GGAGTATATCTCGCAATTTTGTGGTTACCAGAACACCTACACAAGTGGCTAGCCATGCACAA
AAATACTTCATCCGTTTGAACTCAATGAACAAAGATAGGAGGCGATCGAGTATTCATGATAT
CACCAGTGTTAGCAATGGAGACATTTCTGCAGCACAAGGACCAATCACAGGTCAAACTAAT
GGTTCGGGTGCAGGAGCTTCCTCTGGCAAATCAGCCAAACAGCAACCCCAACATCCAGCT
GGGCCTCCAGCTGTTGGCATTTACAGTACTCCTACTATAGGTCAACCAATAGGAGGACCCCT
TGTCTCAGCTGTTGGCACCCCGGTGAATCTTCCTGCTCCAGTACACATGGCATATGTTGTTA
GAGCTCCAGTGCCCGGAGCAGTGGTTCCAGGTGCACCAATGAACGTTGGTGCTATGCCATA
TCCGATGCCCCCCACATCTGCTCATAGGAGTCCTGAAATGACTTCAACGCCTTCATCAATTC

TCTTTCTTACTAATAAAAACACCTTCATACACACTAAGAATCACATTCACACTTCCCTAAATA
CCTCTCAGCAGCATACTTCAAATTCACCGATTTGGACTGCCACTGAGATAGCTGACTCTGTC
AATGGCAAATTACTCAAAAGGTGTCCTCCAGGCATCATTTGTACGGACACCAGAGCCTTAC
AACCTAACACAAACCAGTGGTTTTTTGCCATCACAGGCCAACACTTTGATGCTCATGACTTC
ATTTCTCCCCAGTTGTATGGTAAAGGCTGTGTTGGCGTCATTGGGAACCGTGTTTGCGAGGG
CTGGGACAAGGGTTTTATTCGAATTGAAGGGAATGGCAATATTAACACTGTTGATTCATTGA
TAAATATGGCTTCTTATGCAAGAAATAAGTGGTTTAATGGTGTTTTAGTTGGGGTTACCGGG
AGTGTAGGAAAAAGTACAACTAAGAGTATGATAGCTTTCGTTCTTGAGAGTTTAGGAGTTAA
TGTGTTCAAAAGTTATGCGAATTGGAATAACAGAGTTGGTGTTGCTTTAAGTTTGATTAGGA
TGTTTAGGAATGTTGATATTGCCGTTTTGGAGATGGGAATGAGTAAAAAGGGGGAGATATTG
GAGCTAGCAAGGATGACAAGACCGGACATAAGGATGGTTTTGAATGTGGGTGCTTCGCATT
TTGGAGAGTTTGGGGAGTTTAGAGGAGGTAGCAATGGCCAAAGGCTGCAAAATCTACTGA
GAAATAGAAAGGTGTTGTTTGGCCAGAGAATGGGGTGTGATGTTCGTTTGGTTGCAGCTGA
AACTGCAGATGAAGGTCTTGGAGTTCGAGTTGTTTTGGACAAAGAGAAAGAGATTCCAGG
TCTGCATTTGGCCCTCGATGCATGTGCAGCAGCAGTGGCAACTCTCTTTGGGCTTTCTCTTG
CTGAAGTTGGAAATCTTTTTCGAAATTTGTTCCTGTGCACGAGGTCAGAGCTTCAAGTTGC
CAGTAATGGCATCAAGATAGTCAATGATGCTTACAATGCCAATCCCATGAGCACCAGAGCTG
CCATTGACTTGCTAAAAAGCATTGCTTGTGATGGTAAAAGAGTTGCTGTTTTAGGCGACAA
GTTGGAACTTGGCTCAATCAAGACAGAGTCTCATGAGGAAATATTACGTTACTGTTGTGATT
CCAATATTGATTTAGTTGGCCTTGCTGGAAACAGATTTCTTAAAGCAGCAAAGAATATGAAT
CTAAATAGAGTCAAAAAAATTGTACATGCCAATGATGCTGAAGTCCTTGCGCAGAAAATTG
TGAAGAGATTAAAATTTAATGATGTTATTCTGATGAAAGGTAGTTGTGCAATGCAGATGGAA
AAAGTGGTCGATGCAGTTAATGAAATGGATATATACATTCCACCACAAGAGTTATGA

>MiMYB49

ATGGTAATCCACACGAGTTATCCTCTGATTTCCTACTCAAAAGAGATTGTAGATGGACAGCC
AATTTTTGCTTGTTCAAATTGTCTTCCTGTAAAGGCTTCAAAATATGAACCTGCTGGCCATTC
TTTTCATGCTGCTGCACTTAAACTCCTTGGATGTGAGGAAGATACTAATGCTGATGATCATAA
AGTCTCCAATGATAAAGAACAGACATCTTTGCCACAATATGAGTCTTATAGTAGCAAAGGTA
AGAAGAAATCAGGTACAGCTAGCAATCAGCAGGATCATTATGCATTGCTGGGATTGAGTCAT
TTGAGATACCTTGCCACAGAGGATCAGATAAGAAAAGCTTACCGTGAGACTGCTTTGAAAT
ACCATCCTGACAAGCAGGCTGCACTTCTTCTTGCTGAGGAATCTGAAGCTGCAAAACAGGC
AAAGAAGGATGAAATAGAAAGCCACTTTAAGGCAGTCCAAGAAGCATATGAGGTTTTGATT
GACCCTGTGAAGAGAAGGATATATGACTCCACAGATGAGTTTGATGATGAAATCCCATCTGA
CTGTTCCCCACAAGATTTTTTCAAGGTCTTTGGTCCAGCTTTTATGAGGAACGGAAGGTGGT
CAGTTAATCAACCAGTACCATCTTTAGGTGATGAGAGTACTCCATTAAAGGAGGTCGATAAT
TTTTACAATTTTTGGTATAGCTTTAAAAGTTGGAGAGAGTTCCCACATGCAGACGAGTTTGA
TCTTGAACAGGCAGAGTCTCGTGATCATAAGAGATGGATGGAGAGGCAGAATTCTAAACTT
TCAGAAAGAGCTAGAAAGGAAGAATATGTACGAGTGCGCGCTCTTGTTGACAATGCGTACA
AACGAGACCCTAGAATTCTAAAGAGAAAGGAGGAGGAGAAAGCTGAGAAGCAAAGGAAA
AAGGAAGCTAAATTTCTGGCAAAGAAGTTGCAGGCAGAAGAAGCTGCTAGGGCTGCTGAA
GAGGAGAAACGCCAAAAAGAGGAGGAGGAAAAACGAGCTGCTGAAGCTGCATTACAACA
GAAGAAACTGAAGGAAAAAGAGAAGAAGCTCTTGCGCAAAGAGCGGACTCGTCTTAGAA
CACTTTCAGCATCTGTTATGGAAAGCAAGGAAGGGCTGGAACAAGCAAAAGTTATTAGGA
GTGCACTTGGAATTGCTGATAAATGTGAGGAAAAGAAACAAGATGAGAAGAATAATTTGCA
GCAGAATGGTTCTGTGGAGGCTAATGGAAGTATCCCTTTAAAAAGCTTTGAGAAGAAGGAG
AAGCCTTGGGAAAAGGAAGAGATTGAGCTTTTAAGAAAAGGAATGCTGAAATATCCCAAA
GGAACATCTAGAAGGTGGGAGGTTATTTCAGAGTACATTGGCACAGGAAGAACTGTGGAA
GAAATTCTAAAGGCAACCAAAACAGTCCTCCTCCAGAAGCCTGATGATTCCAAAGCTTTCA
ATTCTTTTCTTGAGAAGAGGAAACCTGCACAGTCCATTACTTCTCCCCTTACAACTAGGGAA
AATGTGGAAGGGGTATCGACTCCTCAGGGGGCTGAAAATACAGCTGCAAAGGTGGATATCC
CAGAAGAGTCTTCAAGTTCAAATAGTCCTATGGATGTGTCTGCTGCAAATGGGGTTTCTTTG
ATTTCTGACCAAGATGTGTGGTCTGCTGTACAAGAAAGGACATTGGTTCAGGCTCTGAAAA
CCTTCCCCAAGGAAACCAGTCAACGCTGGGAGCGAGTTGCAGCTGCTGTTCCTGGAAAGA
CTCTGAATCAGTGTAAGAAAAAGTTTGCACTGCTTAAGGAGAACTTTAGAAACAGGAAAA
CCGTGGCTTAG

>MiMYB50

ATGGAAGCAGAGAGGGAAAGAGCAATCCAAGTCGGCGGTGGAGAAGCTGCAGCCGAGAG
CTTATGTGACGGCGGCGGAAGCGGCGACGATTCTGTAGTAGTTGTCGGAGAGGGAAGCAG
AAAAGGCAGCAAAGACCGAGTGAAGGGGGCTTGGTCGCCCCAAGAGGACACAATATTGA
GCAACCTTGTTAGCAAGTTCGGCGCGAGGAATTGGAGCTTGATCGCCCGAGGAATTTCCGG
TCGCTCCGGCAAGTCTTGTCGGCTCAGGTGGTGTAACCAGCTTGACCCAGCCGTTAAACGC
AAGCCATTTACTGATGAGGAAGATCAAATTATTATTACAGCTCACGCTATCCATGGAAACAA
ATGGGCTGTCATTGCTAGGCTTCTACCTGGGAGGACAGATAATGCTATTAAGAACCACTGCA
ATTCCACTCTAAGGCGTCGGGCACTGGAACTTGGCAGGGTAAAGAAGTTCGAATCTGGAA
GTATCATGGAGGATACCAACATGGATAGGACCAAAGCATCATCTGAAGAAACTCTCTCTTGT
GGTGATGCAAATTCGTTTAAATCCTTGGAGGGAAAAGATGTATGCTCTTTGGAAATTTTGGA
TAATCAATGTGAAAACAACACCATATCAGAGGTTCCATCTAATCTTGAGCATGAACTAATAG
AACAACCTACTCTTTTCCGTCCACAGGCACGTGTATGTGCTTTCAATGTTTACAATACTGTA
GATGGCCCTGAGATTGCTTCACTGTATCCAAGGCAAACCCCAATGCAAGGACCTCTAGGTC
AAGCATCACTGTCAGATGCTGGGATCTGCAAAATGATTGAGGGAGGATACAGTGAGCGGAC
GGTGCCTCACTTGTGTGGCTATGGCTGTTGTGGGATACAATGTGGTGGAAATTGTCAAAACT
CTTTGTTGGGACCTGAATTTTTGGAATTCTCAGAACCTCCAAGTTTTCCAAGTTATGAATTG
GCTGCCATAGCCACAGACATAAGCAACCTTGCTTGGATGAAAAGTGGATTGGAGAATAGTA
ATATGAGAATGATGGATGATGCAGCAGGTAGGATAAGAACCAATGGATCAATTGCAAAGGG
TTGA

>MiMYB51

ATGAAGAAGAGACAGCGTTGGAAAGCTGAAGAGGATGTTTTATTACGTGCTTATGTAAAAC
AATATGGCCCGAGGGAGTGGAGCCTTGTATCACAGCGTATGAACATACCACTAAACAGGGA
CGCAAAATCGTGCTTAGAAAGGTGGAAGAACTACCTCAAACCTGGCATCAAGAAGGGATC
ACTTACTGAAGAGGAGCAGCATCTTGTCATACAACTTCAAGCCAAATACGGCAACAAGTGG
AAGAAAATTGCAGCTGAAGTCCCTGGCCGCACTGCCAAGAGACTTGGCAAGTGGTGGGAA
GTGTTCAAAGAGAAGCAGCGAAGGGAACAGAAAGATAACAGCACAACAGTTGATCCAAG
TGAGGAGCGCAAGTACGATCAGATCCTAGAGACTTTTGCGGAGAAGCTAGTGAAAGACGG
TGCATTTGTCATGGCTGCTTCGAATGGAGGGTTTCTTCACACTGAGTCTCCCACCCCATCAA
CTTTGCTACCCCCTTGGCTTTCCAATTCCAATGGAACCTCAGCTATCAGGCCGCCATCCCCTT
CTGTAACCCTAAGTCTGTCTCCCTCAACAGTGGCAACTACTCCTCCAATCCCATGGTTGCCT
GAGAGGGGGCCAGATAATGCCCACCTTGTTCTGGGAAATTTTCTGCCTCATGGTTCAGTTTC
CACTTGTGGAGAGAACCTATTGGTGTCTGAGCTGGTGGACTGCTGCCGAGAGTTGGAAGA
AGGGCATCGTGCTTGGGCAGCACATAAGAAGGAAGCAGCATGGAGGTTGAGAAGGGTCGA
GTTGCAGTTGGAATCCGAGAAGGCATGTCGAAGGAGGGAAAAAATGGAAGAGATAGAGGC
TAAGGTGAAAGCTCTTCGAGAAGAGCAGAGGGCTACTTTGGATAAAATTGAAGCTGAATAC
AGAGAACAAATTGCTGGACTGAGGAGGGATGCAGAAGCCAAGGAGCAGAAATTGGCCGA
GCAATGGGCAGCAAAGCACCTGCGTCTTTCTAAGTTTCTCGAGCAGATGGGGTGCAGGACT
CGGCTACCAGAGCCCAATAATCGGTGTCCATTAATGTTTGAAATTCTGTTATACTTTCATATTT
ATAATAACCGTAACCTTTACTTTCTTTCTGGATTGAATCTAATGTAG

>MiMYB52

ATGGCAGAGTTGAAAGTAGAGGAGTGTTGCCTTGAGAATAAACAATTAACAGCCGCTTCAA
GCTCTTCTGTATCTGAAGGTAGTGGCAGTGCTATTCTCAAGTCCCCTGTATCAAGCCCTGCA
ACTAAATCACCAACTCACAGGAGGACCACTGGCCCGATTAGGCGAGCTAAAGGAGGTTGG
ACACCAGAGGAGGATGAGACATTAAGAAATGCTGTGGCAACTTTTAAAGGGAAGAGTTGG
AAGAAAATTGCTGAGTTTTTTCCTGATAGATCAGAAGTGCAATGTCTGCATCGGTGGCAGA
AAGTTCTCAATCCAGACCTTGTCAAAGGACCTTGGACTCAAGAGGAGGATGATAAAATAAT
TGAACTTGTGTCAAGATATGGGCCCACAAAATGGTCTGTCATTGCGAAGTCTTTACCTGGTC
GCATAGGGAAACAATGCCGAGAGAGGTGGCACAATCATTTGAATCCTGATATAAAGAAGGA
TGCCTGGACGCTAGAGGAGGAATTAGCTCTAATGAATGCCCACCGGAGAAATGGGAACAAA
TGGGCTGAAATTGCAAAGGTTTTACCTGGAAGGACTGATAATTCCATAAAGAATCACTGGA
ATAGTTCCTTGAAGAAGAAATTGGATTTCTATTTGGCTACTGGGAAACTTCCTCCTGTTGCG
AAGAATGGTCCCCACAATGGCTCCAAAGATACAAATCAATCGACTGCTGCTACTAAACATTT

TTTTATTTATTCAACTAGAGACTCTGATTCAACTGCCCAAACTTCATCAGGAACTACAGACTT
AGGCAAGCTGGATGAAGACGGCAAGGATCAACTAGAGTCCTCAACTCCAGTTCTAGATATG
GCAGCTTCATCCTGTGTTCGTCCAAATGATTCTCTAGATTCTGAAGACGTTGAATGTAAGCT
AGAGTCACCTAATATAGATCTTAGCTGCTCAGAGTCAATGCCAAAGATTGAAAATTGTGCAA
TCAATTCTGAACATGTTGATGACAGAGTGATTGGATCACAACAGCAAATTGGAACTCCAAC
CTACGGCTCTATATACTATGTGCCGCCTCAGTTAAAAAGTAGTGTTCCATTAGATTCAGATCC
TTTAAACACATGTTCTCTGCAGCATGAATGCAACTCTACCCCCATTACTTCACCTATTAGTTA
CTTCACTCCACCTTGTGTGAAGGGCAGTGGTTTAAGTGCAAGAAGTCCTGAATCTATATTGA
AGATTGCTGCTAAGACCTTCCCCTATACTCCTTCTATTTTAAGAAAGAGAAAGTCAGAAGGT
CAAGTGTTGTCTGGTACTAAAATAACTGGAAAAGTAGATGGGGAGACAACAGATACTAGTA
GCCCTGTTGAGCCTACTGGAAAAGATTTCAATGCATCTCCTCCGTATCGGTTAAGATCCAAA
CGTACTGCTGTTTTCAAGTCTGTGGAGAGACAACTTGAATTCACTTTCAACAAGGAGAAGC
ATGATGATAATACCAAATCTCGGGAATTGTCTGTGAATGCAAGTTGTCCTGTTATCGAAGATT
TGCACATAACAAAACTCAGGGTGACCTAG

>MiMYB53

ATGAGGATTATGATTAAGGGAGGCGTGTGGAAGAACACGGAGGATGAGATCCTTAAAGCCG
CCGTTATGAAATATGGTAAAAACCAGTGGGCACGTATCTCCTCGCTTTTGGTTCGTAAATCC
GCTAAGCAGTGTAAGGCTCGTTGGTACGAATGGCTGGATCCCTCCATCAAAAAAACTGAAT
GGACAAGAGAAGAGGATGAGAAGTTGCTTCATCTTGCTAAGCTTATGCCCACACAATGGAG
AACAATTGCTCCAATTGTTGGCAGAACTCCATCTCAGTGTCTTGAGAGATATGAGAAACTCC
TTGATGCAGCTTGTGCCAAGGATGAGAACTATGAACCTGGTGATGATCCACGGAAATTGCG
TCCAGGAGAAATTGACCCAAACCCGGAATCTAAGCCTGCTCGTCCAGATCCAGTTGATATG
GATGAAGATGAGAAGGAAATGCTTTCTGAAGCACGAGCTCGTTTAGCTAACACTAGGGGTA
AAAAGGCAAAAAGAAAGGCTAGAGAGAAACAGCTTGAAGAGGCTAGGAGGCTTGCTTCT
TTGCAAAAAAGGAGAGAACTTAAAGCTGCTGGGATTGATACTAGGAAAAGGAAAAGGAAA
AGGAAGGGAATTGATTATAATGCTGAAATTCCCTTTGAGAAAAAGCCTCCTCCGGGTTTTTT
TGATGTTACTGATGAAGATAGGCCTGTAGAACAAGTTAAATTTCCAACTACCATTGAAGAAC
TGGAAGGGAAGAGGAGGATTGATGTAGAAGCTCAGTTAAGAAGACAAGATATTGCAAAGA
ACAAAATTGCACAAAGACAGGATGCTCCTTCGGCCATACTACAGGCAAACAAGATGAATGA
TCCAGAAACAGTTAGGAAGAGGTCAAAACTGATGCTTCCTGCACCTCAGATTACAGACCAT
GAATTGGAGGAAATTGCCAAAATGGGTTATGCTAGTGATCTTCTTTCTGGGAACGAGGAAC
TGACAGAAGGCAGTGGTGCAACGCGTGCTCTTCTTGCAAATTATGCACAGACACCGCAGC
AGGTTGAGAGAATCACAGACACCATTATTGGGAGGAGAGATCCAGAGTTGCACCCTTCAGA
TTTTTCTGGGGTCACTCCTAAGAAAAGGGAGATACAAACACCTAACCCCATGCTGACTCCT
TCTGCAACTCCTGGTGGTGTGGGACTTACTCCTAGAATTGGCATGACTCCTTCGACGGATGG
CTATTCTTTTGGTGTGACACCTAAAGGAACTCCCATCAGGGATGAGCTCCACATAAATGAAG
ACATGGATATACATGATAGTGCAAAGCTTGAACAAAGAAGACAAGCTGATCTGAGAAGAAA
CTTGCGCTCTGGTCTGAGTAATCTTCCACAGCCCAAGAATGAGTACCAAATAGTTATCCAGC
CACCTGCAGAAGATAATGAAGAACCAGAAGAGAAGATTGAAGAAGACATGTCTGATAGGT
TAGCCCGAGAAAGAGCTGAAGAAGAAGCAAGGCAACAGGCATTACTTCGTAAGAGATCAA
AAGTGCTGCAGAGGGAGCTTCCTCGACCCCCAGTTGCTTCATTGGAACTTATTAGAAATTCT
TTGCTGGGAGCTGATGGAGACAAGAGTTCCTTTGTTTCCCCCACTTCTACTGAACAGGCTG
ATGAATTGATCAGAAAGGAACTTTTAACACTACTAGAGCATGATAATGCTAAATATCCCTTTG
AGAAAAATGCGGAGAAGAAAAAGAAGAGCTCCAAGCGTTCTGCTAATGGATCTGCTGTTC
CTATTCCTGTGATTGAAGATTTTGAAGAAGAAGAGCTGACGGAGGCTGATAAAATGATTAA
GGAAGAAACTCAGTATCTTCGTGTATCAATGGGGCATGAAAATGAATCCCTTGATGAATTTG
TGGAAGCACACAATACCTGTTTAAACGATCTCATGTACTTCCCCACCCGAAATGCTTATGGT
CTCTCAAGTGTAGCTGTAAATGTGGAGAAACTTGCAGCCTTTCAGAGTGAATTTGAGATTG
TGAAGAAGAAGATGGATGATGATAAGGAGAAGGCTTTACATCTTGAGAAAAAAGTCAAAC
TTCTTACACAAGGTTATGAGAAACGGGCTGAAAATCTTCGAAACCAAATACAGTCGACTGT
CAAACAGATGGAGACCACTGGAACAGAACTTGAGTGTTTCCAAGCACTGCAGAAGCGAGA
GCTACTGGGAGCATCAAACAGAATAAATGGTCTGTGGGAAGAAGTTCAGAAGCAAAAGGA
GCTTGAGCAAACATTACAGAAGCGCTATGGAGATCAGCTGGCTGAACTGGAAAGAGTAAG
TCAATGCATAAATGAATACAGAATACAGGCACAAAAACAAGAAGAAATTGCTGCAGCGAA
GCGTGCACTTGAGGCAGCTGCAGCAAATAAAGTCATTGAGCAAAATTCAGAAGCCTCCAA
ATCTTTACCTTCAGGGGAACTTGGAAGGTCAGAGCCAGTTGAGCCATCTCATGGTGAAAAT
CCAGGCCCACAAGTGGGTGCTACTCACATAGATGATGATTCAGGGAAATATCATATAATGCC

TACCACAGATGTGAGTGAAAATGTCGTGGAAGCCAATCCAGATGCTGCTGTCCTGGAAACT
GTAAAAGATAGCGGCTCTGCTGATGAGCATGTCATAGAAGTTCCCAGTGCTGAAGGTTACA
ATGCATCCTTGACTTCCAAAGAGTCAGAGGCCAAGGAAAATTTGCCTATCATGAATGGAGT
TTCTTTGGACAAACAAACCGGAGATGATAGTGCTATTTCAGAACTGATCGACACAGAATTG
GATGGGAAAACAGAGAATGTGGCGATAGGTGATTAA

>MiMYB54

ATGAGTCTTCAGCACTTGGTGAGTGATTGTCATGGCTTCACTGTAGTTCATCAAGATATGAA
CTTTGTGCTTCCTCCTCCTCCTTCTGCTCCTCAACTCTCTCTGTCAAACTCTTTTGGAGTTAT
GCGTCATCATCCTATGGGGGAAGAGAGTAATAGTCCCGCTGCAATAATGGGGCTTCAGATTT
TGAGCCCTTCCAGTTTTAAGCCAGAGGAGAACAGGGGAGCAAAGAGAATAGTTGAAGAAA
GCGATGGGTTTTTAGGGATTGAAAAAAAGGGGCTTTCTTTGAACTTAGGTGAAGAAATAGA
GGAAGTGAAGACCTCTGTTTCTGTCAAAACTAAGCACACTAAACTTTGTGCTAGAGGCCAT
TGGAGACCGACTGAAGATGCTAAGCTCAAAGAGCTTGTTGCCCAATATGGTCCTCAAAACT
GGAACTTAATTGCTGAAAATCTTGAAGGAAGATCAGGGAAGAGTTGCAGATTGAGATGGTT
TAATCAGCTAGATCCAAGAATCAACAGGAGGGCATTCAATGAGGAAGAAGAAGAGAGGCT
GTTAGCGGCTCATAGATTGTATGGAAACAAATGGGCGTTAATTGCAAGGCTGTTTCCAGGGA
GAACCGATAATGCAGTGAAGAACCATTGGCATGTGATAATGGCTAGAAAACTCAGAGAACA
ATCAAGCATTTACAGAAGGAGAAAGCCAACTTCTGCCTCTCAGATTGGACTTGAAAATATG
ATTATCCCAAAAAATAATGCAGGCTGTGATTCAACAACAATCTCAAGCAATGAATCCGCCTC
AACAATCACTGATCTCTCTCTCACTCCCTCTTCAGCTAAAGCTCCACCTGCAATTTTCACTA
GGTTCTCACCTATGGGTAAATGTTCATCTGCAGACAAGGGGATAACAAGGGGAAGCACAGG
AGACGTTGACAAATTTCATGGATATTTCAAAGCAGGTAAAATGGAAGTGGCTATGGCAGTG
GACCAATCTGGGAACTCATCAGATTCAAATTCAGAAGTTTCAGCAACAGATTCAGTAACCA
ACATCAACCCCATCACCAATCTTTCTCTCTCTGGGGAAAATGAGAATGCTTATCAGAAGAAG
ACCCACATGCACTTCATTGATTTCCTTGGAGTAGGAGCCTCTTAG
