# Supplementary material for: Genome-Wide Characterization, Identification and Expression Profile of MYB Transcription Factor Gene Family during Abiotic and Biotic Stresses in Mango (Mangifera indica)
Source: Plants (Basel). 2022 Nov 16;11(22):3141. doi: 10.3390/plants11223141 (PMC9699602; doi:10.3390/plants11223141)
Supplement: Supplementary file 1 [file plants-11-03141-s001.zip › Text S2 Amino acid sequences of 54 MiMYB.pdf]

Text S2 Amino acid sequences of 54 MiMYB

>MiMYB1 GWHPABLA027279

MEDEIIIELVNKFGPKKWSTIAQHLPGRIGKQCRERWHNHLNPSINKEAWTQEEELALIRAHQIY
GNKWAELTKFLPGRTDNAIKNHWNSSVKKKLESYLASGLLEQFQGLPLVGHQNQPVPSSSLG
MQSSGDDSCPKGGTAAEEISECSQDSNVLGLSHSTGNAVMHTREQLLETEESNPPKDHSSSPAS
CSEQYYTSLEDVTFSIPEIPCEVGCSSKFLEQNFVHDAGTFAGDYQFNLQDLPNVSSLGWGHQS
LGLPANCMDSHESHEMVNVPYQNSTELSVPSSIGNLAAGSDKAEHILITDDECCRILFTEAMKD
GCFSLENSAQGLNMVESSLCRSLGIQISEAGRTSPSQAYCPSKPEVLGTSCSQSFLSAPMVLSTD
NGAFVYVGESSELNCHTYGTQDQGFITSGYDAFVYTNESSNSPSDDGKDVLGQQEPSHLSKDS
LKLVPVNSFSLRSDAMIAYSSEDAKPNTQTEQQDAGSLCYEPPRFPSLDIPFFSCDLIQSGSDTQ
QEYSPLGIRQLMMSSMSCITPFRLWDSPSLDDSPEAVLKSAAKTFTGTPSILKKRHRELLSPLSD
RRNDKKLEIDMTSSLARDFSRLDVMFDEAGGIHRASLLSPLSNQKRNSGAFIDEKENLGHAFD
GGQEKEKDLSAVSHDRTSEKDCLCSNSHANETKDSVDIDSKTNANAVATTKTEKQPAGVLVEH
NVNDLLFSPDQTGLKAERSLATAARTPRNQYFKSFGANQGAAPEQSPAIACSPAISKNNNGGNS
SGIKTVRPIPSLAPPGSTVLNAGSDVGIENYNIFGETPFKRSIESPSAWKSPWFMNAFMPGPRIDT
DITIEDIGYFLSPGERSYDAIGLMKQLSEHTAAAYADALEVLGNETSEKTVNEGNSKKSSLDEE
NNGVTHSEHKSDHLASTILVERRTLDFSECGTPGKGKENRKSSTATDFSSPSSYLLKGCR

>MiMYB2 GWHPABLA027993

MAFNRKEVDRIKGPWSPEEDEALQQLVQKHGPRNWSLISKSIPGRSGKSCRLRWCNQLSPQVE
HRSFTPEEDETILRAHARFGNKWATIARLLNGRTDNAIKNHWNSTLKRKCSGSMAEDGNFINC
NGYDGDLGHNNQNQPLKRSVSAGSGVPLTTGLYVNPSSPSGSDVSDSSVHVFSSNHNIFKPVA
RAGGVNVDVNVIPETTSSSNDPPTSLSLSLPGADSSEDTNQKAAESSKAINTKSLFPESGAKGG
GFLGFSQEFMGVMQEMIRQEVRNYMASLEQQRGGVCYQSSGGGGADGVFRNRGRGGQWMI
TELGALD

>MiMYB3 GWHPABLA028858

MRTVLFSAPQPTSSKSRISKLSKEDEIIIELVNKFGPKKWSTIAQHLPGRIGKQCRERWHNHLNP
SINKEAWTQEEELALIRAHQIYGNRWAELTKFLPGRTDNSIKNHWNSSVKKKLDSYLASGLLE
QFQGLPLAGHPNQPMPSSSSRMQSSGDDSCPKGGVEAEEISECSQDSNVLGPSHSTGNVVLHT
REQVLIPEECNPAKDQNSSPASCSEQYYTSLEDVTFSIPEIPCEVGGSSKFPSKILCMMLGLLRRV
TSSDKTENILITDDECCRVLFAEAMKDGCFSLENFGQELNMVESSHCHPLDIQISEGGRTSLSQA
YCPSKPEACKAFIYTNESANSPCDDGADNSGLQGPSYLSKDSFKLVPVNSFSSKSDAMIACPSE
DAKPNMQTEHQDAGSLCYEPPRFPSWDIPFFSCDLIQSGSDMQQEYSPLGIRQLMMSSTNCITP
FRLWDSPSRDDSPEAVLKSAAKTFTGTPSILKKRHRDLLSPLSDRRNDKKLEIDMTSSLAQDFS
RLLSPLSNQKRKSGAFVEDKENLGHAFDGGQEKDKDQSAVLHERTSEEDSHCSNSNANVKKD

NVDADTKTNANAGSTTKTEKQTAGVLVEHNVNDLLFSPDQAGLKDETALATTSPTPRNQHFK
SFGANQGVAPEHSPASACSPAISKKNNGSNSSAIKTVKPIPSLTPPGNTLLNVGSDVGFENYNLF
GETPFKRSIESPSAWKSPSAWKSPWFMNSFVPGPRIDTEITIEDMGYFMSPGDRSYDAIGLMKQ
LSEHTAAAYADAMEVLGNDTSGKSVNEGNTKTLSLDEESNNVTHNEPESDHLASNILIERRTLD
FSECGTPGKGIDKGKSSTAIDFSSPSSYLLKGCRRKVFIFSQRYLVCEEVLEYVNLGTGLKGILSL
RVFEV

>MiMYB4 GWHPABLA000111

MTASSDIYSTTTTCLNNSSMEVFSHMGSLHAAPTTTNSEKNSLSNLEDSDGFEENTYNLSEDHH
VVKEMHSGGQSKSSSRGHWRPAEDTKLKELVAFYGPQNWNLIAKKLQGRSGKSCRLRWFNQ
LDPKIDKRAFSEDEEERLMAAHRVYGNKWAMISRLFPGRTDNAVKNHWHVVMARKFREQSS
AYRRRKLNQAAVHNTDQNCAQNMSNVSFINPAKSLDSFAPPIAAGGGDLFLGSTSSCNNFNFLP
VHDDQNQRPNFAACGNQKPPFGYNGDYMSLGLEQDSSSFNIFKPYTVDNNFPMQQSNYQHHS
AAFSETMASASASAASPVSITEPSSSSLSLADTTATSHFETNISPPFIDFLGIGAAS

>MiMYB5 GWHPABLA000367

MVHNSSLFSSWTREEDKLFENALVLFPEETPERWEKIASKVPGKSSDDVQKHYEDLVRDLREI
DSGMVELPNYEDEMDSPRWSSESGTNQMWPGGKSKERETERRKGVPWTPEEHRLFLYGLDK
FGKGDWRSISRNAVVTRTPTQVASHAQKYFLRLNSMKKEKKRASIHDITGTVDSKSLGQSNDE
KWATEPRNQQYNKNHGTSIGYQGFGYPM

>MiMYB6 GWHPABLA000787

MKWETGVISPPTYLSSNNRALEDSKTTKWTAQENKMFENALAIYDKDTPDRWHKVAAMIPGK
TVVDVIKQYKELEDDVSNIEAGLIPIPGYNNTTNSQFTLEWVNNHNSYDGFKQSYGIGGKRSSS
VRPAEQERKKGVPWTEEEHKLFLLGLKKHGKGDWRNISRNFVTSRTPTQVASHAQKYFIRQLS
GGKDKRRASIHDITTVNLNDTRTPLDNQSPPSPDQSAGLSQQPSSGALSSSQFQWHQPNSGSA
MSFLSTQGNMLMASPYAMASYGLKMPGQNLQRGAVHEPYFGLQNLAFQMQYPHG

>MiMYB7 GWHPABLA001571

MNSDFITGLKFLIDNRNTGLRVSVLCRNLLREVTTTTLTLEMEFEILGLEGAFSETKEMTELQV
DECCLENKRLTIASSSSVSEGSGSAFLKSPGVSSPATTSRTHRRATGPIRRAKGGWTLEEDETLR
NTVATFKGKSWKKIAEYFPDRSEVQCLHRWQKVLNPDLVKGPWTQEEDDKIIELVSRYGPTKW
SVIAKSLPGRIGKQCRERWHNHLNPDIKKDAWTLEEELALMNAHRRYGNKWAEIAKVLPGRD
SDSTAQTSSESRDLGKLNEDGKDQVESSTLVLDMAASSSVRPNDSLNSDFVECKPQSAKIDLSC

SESMPKLENCAINSELVDNQVIGSQQQFGTPTYGSLCYVPLYSDPLNKCLLQHECNSTPITSPIS
YFSPPCVKGSGLSARSPESILKIASETFPYTPPILRKRKSEGQASSDTKIIGKVDGETTDTSTPIEST
GKAFNASPLYRLRSKRTAVFKSVERQLEFTFNKGKHHDNTKSRELSVNASCPITEDLHTTKLR

>MiMYB8 GWHPABLA001575

MKERQRWKAEEDALLCAYVKQYGPREWSLVSQRMNTPLNRDAKSCLERWKNYLKPGIKKGS
LTEEEQHLVIRLQAKHGNKWKKIAAEVPGRTAKRLGKWWEVFKEKQQREQKDNSTPVNPIEE
RKYDQILETFAEKLVKGGAFVMAASNAGFLHTDSPTPSTLLPPWLSNSNGSSTVRPPSPSITLSL
SPSTVATTPTIPWLQPERGPDSASLVLGNLPPHGSVPSYGENLFITELVECCRELEEGHHAWAAH
KKEATWRLRRLELQLESEKACRRREKMEEIEAKVKAIREEQRATLDRIEAEYREQIAGLRREAE
AKEQKLAEQWAAKHLHLAKFLEQMGCRPRLAEPNGRLANVKSSLKYAFCD

>MiMYB9 GWHPABLA001647

METQNITDIEVDGDDTEDGGEVEADGGGSGEDSAVVVGEGSKRGNKDRVKGPWSPEEDAILS
NLVSKFGARNWSLIARGISGRSGKSCRLRWCNQLDPAVKRKPFTEKLKLGLGRYRHTPSSIRQT
DRSKFISDEEDKIIISAHAIHGNKWAVIARLLPGRTDNAIKNHWNSTLRRRAMELDRVKKFESG
NIIEDTSMDRTKASSEETLSCGDVNSFKSLEGKDVCSLEILDNQCEDNTITEVPYNPDHELKEQP
TLFRPQACVSAFNVYKAVDGPEIASPNSRQTPMQRPLVQASLPDAGICKLIEGVYGERMVPHM
CGFGCSGTQCGGNGQNSLLGPEFLEFSEPPSFPSFELAAIATEISNLAWLKSGLENSNMRLMDD
AADRIRTNGSQVQMGHLEERKSKLTGQ

>MiMYB10 GWHPABLA001674

MNRGVEVLSPASYLQNSNWLFQESKGTRWTRQENKLFENALALYDKDTPDRWIKVAAMIPGK
TVGDVIKQYKELEEDVSDIEAGLIPIPVYCNDSFTLEWVNCDQEFDGLRQLYSPGGKRGTTTRP
SDQERKKGIPWTEEEHRQFLMGLKRFGKGDWRNISRNFVTTRTPTQVASHAQKYFNRQLTGG
KDKRRSSIHDITIVSLEVTGYSSPENSKPSSANSSTAVIQLQQQPKLTSIRNERFDWKPPNEGAA
MVFNPTNSNVFTPPFCGISSYAAKLQEQNLLGGTLHGSQCDMYSTFYPMQSMQRQ

>MiMYB11 GWHPABLA001867

MVIHASYRLISYSKEIVDGQQIFVSSNCLPVKSSKYEPAGHSFHAAALKLLGCEEYTDSDDQKV
SNDKEHTSLPQYESYSSKGKKKSGTGSNQQDHYALLGLSHLRYLATEDQIRKAYRETALKYHP
DKQAALLLAEETEAAKQARKDDIESHFKAVQEAYEVLIDPVKRRIYDSTDEFDDEIPSDCTPQD
FFKVFGPAFMRNGRCFKSWREFPHADEFDLEQAESRDHKRWMERQNAKLSEKARREEHSRVR
SLVDNAYKRDPRILKRKEEEKVEKQRKKEAKFLAKKLQEEEAARAAEEEKRRKEEEEKLAAE

AALQQKKLKEKEKKFLRRERTRLRTLSASIVSQHLLDLSEEVVESLCMSLDTEKLRSLCDKLEN
KEGLEQAKVIRNALGCVHECEGKNQDEKNNLQQNGSAEANGIIHLKSFEKKEKPWGKEEIELL
RKGIQKYPKGTSRRWEVISEYIGTGRIVEEILKATKTVLLQKPDGAKAFDFFLEKRKPAQSIASP
LTTREDVEGLSIPQGPQETAAKVDIPEGSTSSKSPVVSALDGVSSSSDQDVWSAVQERALVQAL
KTFPKETSQRWERVAAAVPGKTVNQCKKKFALLKENFRNKKSSV

>MiMYB12 GWHPABLA002362

MLIDDKSNDQQQQLENGSSGMNVEGGRDGGVQLKKGPWTAAEDSILTEYVRRHGEGNWNA
VQRNSGLARCGKSCRLRWANHLRPNLKKGAFSPEEERLVVELHAQLGNKWARMAAQCFSRS
RTQIPTSLSPTPPQLSPTIQSPTFPTLSLFNSTNGYQTNHPNSATNMSNAFIISRAPPILQNPIRFKR
FHPSNNSQNMENSHNTMNLSYQNKNFSPPFSPQFLRNPSSPILTPQSTNSNIFSNNSTLLSQPSFS
SLPVNFNAQNSSMIPSLVEKDGFLSSDSVGFSLNPELPSIQALSQQNGNGFDNEKLSVSSSGSGL
LEDLLEEAQAMASNYGNSSKQLSGVDSLEDQNKPEFGGFSQWSDSNSVNISSGMKPKEENEQ
GQMSTMHEDLSKYLNEIPSTQVPEWYNDSGEASNGQSPVVTDDNFGLDMQQIASLFRVDSNT
GQVSDNQGSCPFDNLLGIC

>MiMYB13 GWHPABLA004872

MPFHGEDDGEEFSGSENDDGFDEEEREAIRRACMISGTDPNDLDNTNKLQLTVAADSSGGVSA
DYWSSDSEDDRELVRKIQNRLALSDDSCQPLCALPPVLLDDDEEDDFETLRAIQLRFSAYNSAD
TTKGSWKDSLQTPNQVFASSVASKNETSNGLFVNRINSCEGFPDSEEACNNHHLSKVNVETLPS
GSIEWQQSEKYNLSMLSQNNSNFPKSAQMFIDAIKKNRSYQNFIRSKLTQIESRIEENKKLKERV
KILKDFQVSCRRITGRALSQKKDPRVQLISSQKLRNGNDSEVFDKKSSAIHYGPSENSHVSNYR
MALSRYPLSLHRKKWSKTEKENLGKGIRQQFQEMLLQVSMDRLSGSEGSSMDTNGLDNIFASI
KDLDVTPELIREFLPKVNWNQLASMYVKGRSGGECEAQWLNFEDPCINHNPWTIEEDKKLLLI
IQEKGISNWFDISVSLGTNRIPFQCLARYQRSLNASILKREWIEDEDEQLCMAVEAFGESNWQSV
ASTLKGRTGPQCSNRWKKTLHPMRQRVGKWTPDEDKRLTVAAILFGPKNWKKIAQFVPGRTQ
VQCRERWVNSLDPSVNRGEWTEEEDLMLEDAIKKHGFSWSKVAAALPARTDNQCWRRWKYL
HPDEVHVLQARRKMQKAALMSNFVDRERERPALGPNDFMPLALTNPASEHGDVIASQKHERK
SRRKAESGNKEDAAPCENQKTRKSQRTRKKTQICSEVLEITYGDDTETSNQRGVILKKKIVKLH
SGKKKVNSEPNTVKKVSKRCSKMPSCAELDEMSILLPPPESVEAEITFTDSSNTPCGNTNVSNC
DSIDPIWNSAGNLCSISCQEQDAPYCSEVGILRGTIDDIELLQNHSDYLDSTLSTTINSEDGDLLG
GIDASTKKRPSKLLPKKKSGMKSGKERTEGENISLESVCETVKRNNKRKICNEPSGKRQDVASI
SCEQAGSKTLLETSDGEDITLECFLCNKSKKRKLKAGSSSESLLLSKPVDQHISEGKICNEPSGE
LQDVASISCEQDGSKQCLETSVGEDITLACFLRNKLKKRKLKAGSSSKSLVSKLVNQHINEDHM
PAHLQNGEAETTNGSSDEPVTKLKSINLEGDGEVGEII

>MiMYB14 GWHPABLA004942

MCTRGHWRPAEDEKLRELVERYGPHNWNAIAEKLQGRSGKSCRLRWFNQLDPRINRSPFTEEE
EERLLASHRIHGNRWAVIARLFPGRTDNAVKNHWHVIMARRCRERSRLYARRAAQNLLNNEQ
KSPSNPEIFSAYCQKNLYSFAQNHNHLLHQCYPVQVFKEVEAENTSHCITMNQDNKNQRVEFY
DFLQVNTDSTKSEVIDNARRDDEEVDQEATEQQKTRAQFIDFLSVGGD

>MiMYB15 GWHPABLA005064

MSNNSASSWSRDEEKAFENAIAMHWNSSSSTATTTSEVGDSSSLEEQWQKIASMVPSKSIEELK
QHYQLLMEDLREIEAGHVPLPNYVGELEGASLATSSKDFLGFSGSVSGEKRSNCAYGSGFTGLS
HESSGHGGKGGSTADQERRKGIPWTEEEHRLFLLGLDKFGKGDWRSISRNFVISRTPTQVASHA
QKYFIRLNSMNRDRRRSSIHDITSVNNGEVSSHLHHHQTPGQQGDRNPGGGPAMGASVKHRG
GQPHLLGLGMYGAPVGHPIAAPGHMAVGTPVMLPPPGHHPHPPHVVPVTYPMAAPPAMHQ

>MiMYB16 GWHPABLA005313

MEFDSSFREDYPFLSSLLADNPLSNPEFKNGFSSLIDNTSSPSLNRGLFELDHDSLMNPESFNQL
NIEGSSKNPFRVSRPSFDPFEVNTNVFSADHVVDCNPLMANGLLHDSERSAFWAQSVPEAQIYE
PTKFQEFGSAAARLPDEVSCITADQNATYHQKVEQKRHKKIQTRRSGKAPKKHSLIKGQWTAQ
EDKTLVRLVGQYGTKKWAQISKMMNGRVGKQCRERWYNHLRPDIKKEAWSENEDIILIESHK
QLGNRWAEIARRLPGRTENTIKNHWNATKRRQQSKRKNKDSTPKSTLLQSYINTVTYSTSFIKK
GKKPMKKTNVQQLVSNAIMPTPQIHQMETSDFTADWQVPSAPAAYDQNEAMGYSFDASMLC
EGFNFGSMLDEATCGSMVDESNNEFELPLEIDSFSQEDQLRKEMEMMEMVCKGLAG

>MiMYB17 GWHPABLA006538

MDRVSSSDRIKGPWSPEEDQLLHQLVQRHGARNWSLLSKLIPGRSGKSCRLRWCNQLSPEVHH
RAFTSDEDEIIVKAHAKYGNKWATIARLLNGRTDNAIKNHWNSTLKRKYATMTTEDENDDTN
ERESFLHQEKKRLAGVSFSDISSCPSGSDVSDSGLPMMSGSDMCVPTGRFVGAETGNDGNNGN
DDAVFNVSTELTLNFPGNKSTELRENDFTRCWVRGEVSQKENNSEYDNHKTTAFGPELMALM
QEMIRKERLLIPSGEEPVLDLTHVGFIILSSETYLLCMISEKNRVPDSFEFAWVKYSAPYRAEEDL
RSEPNLKSTKKRITNSERALQREEMLPTSVSKGRSSSATSRSNSMLPQYLRRIIKVLAKNF

>MiMYB18 GWHPABLA007103

MPPEPQPWDRKDFFKERKHHNNNNHHDRTASDSLLGGGGGPLTRWKDYSSFSSHSRELPRWG
SVDSRRPTATGNLSLVSTTEIINFTSKLLSDSQIKVYRDFLKMPALILDKKEKMVSRFISSNGLVE
DPCAVEKERAMINPWTVEEREIFMNKLATFGKDFRKIASFLDYKTTADCVEFYYKNHKSDCFE
KIKKRPEQMKQCSNTYLIASGKKCDRQMNAASLDILGEASEMAATFQANNARQICSVRISLGG

RSDSKLSLGDDGMTERSSSFDVHGNERETAAADVLAGICGSLSSEAMSSCITSSVDPGEGQRE
WMHQKTDSVRRWHSTSDVSQNAADDTCSDDSCGEMDPADWTDEEKSIFIQAVSSYGKDFAMI
SRCVRTRSKDQCKVFYSKARRCLGLDLIHTGCGIAGTTVSDDANGGGSDCEDACVLEASSVIC
SDKVDSQMNDLPSSGVNKNQDPSCAGPMSLQTDLNKTEDDNGVRHFDDPRSKDVGPLVSDE
CKILHSPEVVSEFERRKMDDVDRQSKSLQAQKLPVLLVEKEVEKDKLTEQAVCVPVPAMIREA
LKSYPPGLNAVVETTEAPVKGFQNGLEEQNEHKTCSADSSDRDMMRGSNVGNIFDLAIETSSC
SVPLKLDSGDKLPVMSLPPENSLAPATSVAHDSAAIECKKTAKQNRLSTKLDFEGNKDKNADIS
VGSEDYHHNRMGHSIVNHVEHSQILKRYLSQISAKKGMNGDIGCRQLSEVQGISKPDRSNSVH
VAQGCHVQKGTSSLSHAAVTEFPLVLPNLDQKNDPPRSHLLGLTEVDRPCKNGDVKLFGKILS
HPSSSQKPNSCSHETEEKGTHHHKQSCKVSNMKLTAPRPAEMLKFDCNNYPGLENVPLGSYRF
WDGNRIQTGCSSLPDSAVLLAKYPAALGNYPAACSSKLEQQALQVKSSECNMNGVAVLPPREI
SSSSNGVVDYQVYRSRDNSKVQPFTVDVKQRQDLLFSEMQRRNGFEGLPTLPQTGRGMVALN
VVRRGGILVGGPCQGVSDPTAAIKMHYAKADQYAGQSGTIIREEESWRSNGDLGR

>MiMYB19 GWHPABLA007174

MQLFLDEVLENGCSLQEVFCHGKIKLLNMGTLYPALYMLDSSWILQESHSTSWTKEENKRFES
ALAIYNEGTPDRWIKVAALIPGKTVLDVIKQYKELEEDVKNIEAGKVPIPRYYGSSFTLEFVSER
DFDANKKRPLVKSLDQDRKKGVPWTEEEHRLFLKGLLKYGKGDWRNISRHFVITRTPTQVASY
AQKYFIRQLSGGKDKRRPSIHGITTINLADPNSSDNRKPCSFDLSDVLLQQQKSSDSPEVGLKW
NDSNKGAVNLIRRVITFLCRLPLTLVQMASNFREKLCMPLFIMGFSSTRKSVF

>MiMYB20 GWHPABLA007175

MSGSSWFLQESQSTSWTKEENKRFESALAIYSDGTPDRWLKVAALIPGKTVLDVMKQYKELE
EDVKDIEAGKFPIPGYYGSSFTLELVSERDFDADRKTPLVKSSDQERKKGVPWTEEEHRLFLKG
LLQYGKGDWRNISRHFVITKTPTQVASHAQKYFIRQLSGGKDKRRPSIHDITTVNLADPNLSDN
RKPCSFNLSHELPQQQKSSGLLKAGPEWNDSNNGAVIFDTSHDNLFVPSAFDIGSNGLKLQGKS
LYGTAYHGVQFNPEISVLNPI

>MiMYB21 GWHPABLA007189

MTDKENESNGDAEVVAEETVEVGCGGGGGGGGAEGSRNGGASRVKGPWSPEEDAVLSRLVS
QFGARNWGMIARGIPGRSGKSCRLRWCNQLDPCLKRKPFTDEEDRVIIQAHSIHGNKWAAIAR
QLPGRTDNAIKNHWNSTLRRRYAEIGRFKPGSGDMMEDGSHDRTKVSSEETLSAGDVNSFRPP
EGRDVMMDDSPNQHEDIAQTKECQLFAGPNHRPAFSRPVGDMSPISLPEGRDITMNDGPNMH
EGIAHRKETEGDSGPKHPTLPRPVPRVSAFSFYNPPSGPTSSSEIPWTIPTQGPLVQVPRPDFGLK
FVEDVHSEPVIPSQCGYGCCTSPLGHHSHGSLLGPEFVDYEEPPAFSSHELISIATDLNNIAWIKS
GLESCSVLVPGNAAASHKMSEGTTDSSQIRISERGVRKDMHSDEGRNKLMGMMTDVLSVQVP
AQTYAGRPEVEGLS

>MiMYB22 GWHPABLA008377

MDDSGAASGDDATKTCPRGHWRPAEDEKLRQLVEQYGAQNWNSIAEKLQGRSGKSCRLRWF
NQLDPRINRRPFTEEEEERLLAAHRIHGNKWALIARLFPGRTDNAVKNHWHVIMARKQREQSK
LCGKRTYSESLSSSNDISFDHFSSNHISRKLRPQDMFIGSRNIGFAASPAVSQLSWTFAPPMVATS
NSNISSSAVEFKKKEGRDYLTSTSNYYSSSSVRSSSSIIGLTNSRRIVSPFGSFRVGDHLDYESHGK
KKELIKFSDNSMRVSAVQQAQGDESIHHKTVPFIDFLGVGISS

>MiMYB23 GWHPABLA009654

MCSSSTSSSDSSSSESSFSGNNRIPRETHKAERIKGPWSAEEDRILTRLVERYGPRNWSLISRYIK
GRSGKSCRLRWCNQLSPNVEHRPFSPAEDETILAAHARFGNRWATIARLLPGRTDNAVKNHWN
STLKRRAKEQQLQQQQQPLEQQQMDFFDNGNNNIGHAASGSGQQCVGLEDDSLTALTLGPPG
SSSAAVTERRTEGFPAGFWDAMRGVIAREESGRPGI

>MiMYB24 GWHPABLA009746

MAVLMESDYTCKLGRPGDDACLFDSCILVTALNGYDRFFFLKNLNSMASTRKDVDRIKGPWS
PEEDEALQRLVQSYGPRNWSLISKSIPGRSGKSCRLRWCNQLSPEVEHRPFTAEEDDTIIRAHAH
FGNKWATIARLLNGRTDNAVKNHWNSTLKRKCSSLSDDLNEDANNIQPLKRSASVGPANNISG
LCLNPSSPSGSDLSDSSLPGVQSPVYRPLLRTGSLAPVEASSSTTDPPTYLSLSLPGSGLDSCEFS
NHGSGSASRPVLSSNNMVQLQVAQPPLVQSHSEGFEKQFFSAEFMAVMQEMIRKEVRNYMSGI
EQNGLCIHTEAIRNAVVKHMGVSKID

>MiMYB25 GWHPABLA010233

MDDSGAASSDDATKTCPRGHWRPAEDEKLRQLVEQYGAQNWNSIAEKLQGRSGKSCRLRWF
NQLDPRINRRPFTEEEEERLLAAHRIHGNKWALIARLFPGRTDNAVKNHWHVIMARKQREQSK
LCGKRTYSDTLTNSNQFSNNHISRKSRPEDMFFSSRNIGFQNSRIFDFRNTNSDINRTFAVSPSVS
QLSWSFAPPMVATSNSIISPSSVDFSRREGRDYLTYPSSSVYGSNWSSSIIGLTNNRRIVQRPFGSF
RVGDLHYDSHGKKELIKFSDNSSLRVSAVQQDQGDESIKHDSVPFIDFLGVGISS

>MiMYB26 GWHPABLA011031

MQEITKKIGGANEDSKKKERHIEDNILREQITIHGTDNWSIIASKFKDKTTRQCRRRWYTYLNS
DFKKGGWSPEEDMLLCEAQKIFGNRWTEIAKVVSGRTDNAVKNRFSTLCKKRAKYEALAKEN
NNPYMNQNNKRVLFQNGFNADGTRENTELIKRARRSHIPDLAESHNIANRSHRESGTTMNPRF

RPPFTVLVQNLPSVNGLPASHCDMKEVSNNAAQNNKTQGMFLRKDDPKITALMQQAELLSSL
ALKVNTEDTEQSLEDAWKVLQDFLNRSKENDILRYTISDIDFQFEDFKDLIEDLRSTNEESHLS
WRQPDLYEDSPASSEYSTGATLIPHPACDKPDQIQAEVDALHRDIVAELQSIHIGEQHCLGQQNK
GSANTNEVVLFPSGGEDTNNTGAVSVSSSTAFSSPLQVTPLFRSLAAGIPSPKFSESERNFLLRTL
GIESPSPNPSTNSVQPPPCKRALLQSL

>MiMYB27 GWHPABLA011054

MASLSLSPNYGSAVSVVSASLETHICRNTDMENAGACWGFPFMGNSSTRSFEGQHNIDANAEV
KCSDYDDRLGEASETIIHNVNLNEEFNPNEKEIDSGQAKLCARGHWRPAEDTKLKELVALYGP
QNWNLIAEKLEGRSGKSCRLRWFNQLDPRINRRAFTEEEEERLMQAHRLYGNKWAMIARLFP
GRTDNAVKNHWHVIMARKYREQSNAYRRRKMSQSIYKRMEDIPGVICRDATARTEPQPYCFSV
PNGGFGNTSSFCGSGGVEFAFNGSPHMTSGQETILSNKDPYSGFCVQQTPSDFFPGPKGSDMM
AMFSQVRSWDGPIDEPHVYGFNLHHHPQNLMAMQQSNFQHHQLHSFFGSTASTPQVSASEAS
SSVDNHFDPVPPRFFDFLGVGAT

>MiMYB28 GWHPABLA011331

MTVDEVGSSSEWTREQDKAFENALATYPEDASDRWEKIVADIPGKTLEEIKHHYELLVDDVNR
IESGCIPLPSYKSSSDGSTGHGGDEGTGKKGGHYGHYNSESNHGSKSSRSDQERRKGIAWTED
EHRLFLLGLDKYGKGDWRSISRNFVVTRTPTQVASHAQKYFIRLNSMNKDRRRSSIHDITSVSN
GDITAAQGPITGQTNGLASGASSGKSAKQQPQHPAGPPAVGMYATPTIGQPIGGPLVSTVGTPVN
LPAPAHMAYRNRAPGPGAVVPGAPMNAGAMPYPMPHTSAHRSCKEPVSHKSIYITVFELPCMI
GGLSGIIFSIELGISTTRFLTIKNTFRFH

>MiMYB29 GWHPABLA012350

MEAHMGGFSSSTCDQRSEEEVDVRKGPWTFEEDSLLMNYVTIHGEGRWNSVARCAGLKRTG
KSCRLRWLNYLRPNVRRGNITLQEQLMILELHSRWGNRWSKIAQYLPGRTDNEIKNYWRTRV
QKQAKQLKCDVNSKQFRDAMRYVWMPRLLERIRATTTDSSTIQPTSNLTTTTESTSTHEDKSTS
QVHHSTELSCSSWDSVDAHVSPASDANLDNSQNWSELYLNNMSGCVSGSWDWSKPGLDING
FETQNNNEWIGSDESFESLWNDENNWLWQQQQYDH

>MiMYB30 GWHPABLA012843

MEGRREEIRKGPWKAEEDEVLINHVKKYGPRDWSSIRSKGLLQRTGKSCRLRWVNKLRPNLK
NGCKFSLEEERVVIELQAQFGNKWARIATYLPGRTDNDVKNFWSSRQKRLARILQNSATPSSSS
NSKSHRATREVSTFNDVPTLLAPKLSSSMDEESSSKDHSCSSSNREKAGTIKMVPVPALVNPKL
LHFDAHSVLTPFENNPCTDSQLQIPFPDITQTQHDLTFSPESQELLAGLEDPHFFGVFGPVDASQ

LSNEVPLPVEEPFLKPMRSFRNAVKDENDNLVNPDAFFEDFPTDMFDHIEPLPSPSDW

>MiMYB31 GWHPABLA013290

MKPCIKTEIPFDEGPSSKGYLQDFHHLDTHFRANGCSSNPMFGVQTPYLDSLEAFSAAYPCSLS
NFDVYECNPFADNFHSGGSLEHHQVINPQLVDITGSSQSRMPISYQEIKPVNFAGSDEVSCVSA
DDIASYYKKANMDTINSKAYLSTRSLKAGKKYKVVKGQRTIDEDRLLIRLVEQHGMKKWSHI
AQMLPGRIGKQCRERWHNHLRPDSKFTFFHLQHGIRGGALSFLLKDTWSEEEDKALIEAHAEI
GNKWAEIAKRLPGRTENSIKNHWNATKRRQFSKRKCHSKNQTTSLLQDYIKTLNFTSTSTKHQ
AKTSSKLVLEKTPADDDFCLSDHLVSCAPVDADKKSLEMELPLIDTEYQVKKDLDLVEMIVEA
NNL

>MiMYB32 GWHPABLA013390

MAPKNDGSAKKVMNKGAWTAEEDRKLAEYIEIHGAKRWKTVATKSGLNRCGKSCRLRWLNY
LRPNIKRGNISDEEEDLILRLHKLLGNRWALIAGRLPGRTDNEIKNYWNSHLSKKINKVKPPQPE
IVQEAALQDTSNINEFKEEGEDSKGIGNFEVDFDVNELFDFSTEGSYGLEWVNEFLELGEESWL
TENKMSI

>MiMYB33 GWHPABLA013713

MSNNSASSWSKEEEKAFENAIAMHWNSSTSIATSEEDSSSSSSEEQWQKIASMVPTKGIEELKQ
HYQFLMEDLQAIEAGRVPLPDYVGELEGATMATTSSSKDFHGFSGSVGGDKRSNCGYGSGFTG
LSHESSGHGGKGGSKSDQERRKGIPWTEEEHRLFLLGLDKFGKGDWRSISRNLVITRTPTQVAS
HAQKYFIRLNSMNRDRRRSSIHDITSVNNREVSSHHHQALITGQQGNTNLGEGSAMGASGKHR
GGQPHMPGLGIFGAPVGHPIAAPVHMASAVGTPVMLPPPGHHPHTSYVLPVAYPMAPPTTTHQ

>MiMYB34 GWHPABLA020355

MTLQQFHKSSFCDQDNDSSNIYSTSSIPNNSSMEVFSHMGSLHAAPTNTNSVKNSLSNLKDPD
GFEENVHSLSSDSNGVKEMTVSGGQSKICSRGHWRPAEDIKLKELVALYGPQNWNLIAEKLQG
RSGKSCRLRWFNQLDPKINKGAFSEEEEEKLMAAHRVYGNKWAMIARLFPGRTDNAVKNHW
HVVMARKFREQSSAYRRRKLSQVVVHNTAVSFLNPSSKSFNLFAPPIDGNGADDGFSDHHHQP
ANLMDGRDLFLGSTSSCNFNFLPLHDQNQTPNCASCGNQKAHIDLLSGYKHCDMEKDSSSFNI
FKPYTLNNNFPMQQPNHHHHSATFSDTMALASASASASSSLSLADATATSHFETNISPPFIDFLG
TGAAS

>MiMYB35 GWHPABLA020548

MQIHKDPKAPPGNDNDEMEWQRWQPEEEAILREYVKQYGPKEWNLISQRMPKPLNRDPKSC
LERCKNYLKPGIRKGSLTPEEQSLVISLQAKCGNKWKKIAGEVPGRTAKRLGKWWEVFKEKQ
WEQLQKQRRDYSDEEGNNVVRVTSASVSSPEKVAQGRYDHILETFAEKYVQPKLSLPDPEPILS
LGSGPSSASRNVLPLWMNSHSTCSSLSSSASSTTTASPSVSLSLFPSEPPTLDPVDMTWFIPGQQ
MGTLIQWCKEVKEGEQSRLQHKKEAKWRLSRLEQQLESEKARKRREKTEEAEAKIRSLREEE
MAFLGKLESEYREQSTSLQRDAESKEAKLMKAWCNKHVKLVTLIEQFGVHSCHGNGFCTEKE
GKKDATLAIQRQANATSYSEMTNHWALG

>MiMYB36 GWHPABLA022091

MSLQRLMGDAHGFSVVHQDLNFVPPPTSPPQLSLSESFGVLGHHAMGIETNNPAIMGLQILNPS
GLKPEENRGAKKTVEERDGLFGSEKKGLSLNLDEEIEEVNTSVSVKTKHTKLCTRGHWRPAED
AKLKELVAQYGPQNWNLIAENLEGRSGKSCRLRWFNQLDPRINRRTFNEEEEERLLAAHRFYG
NKWALIARLFPGRTDNAVKNHWHVIMARKLREQSSIYRRRKPNSASQTGLENMIINKNATSES
TNSSNESASTITDLSLTPSSTKFSPAIFTRFSLMGSSKEKEITRGSIGDVDKFYGYYKAGKVEVV
MGVDQSVHSSDSNSEVSATDSVTNNNTNTNTNLSLAGQNTSQKNISMAFIDFLGVGAS

>MiMYB37 GWHPABLA022650

MKWETEVISPSPYLSTNNWVPEDGQTTKWTAQENKMFENALAIYDKDAPDRWHKVAAMIPG
KTAVDVIKQYKELEADVSNIEAGLIPIPGYNNTTSSPFTLDWVNNHNSYDGFKQSYGIGGKRSS
LVRPAEQERKKGVPWTEEEHKLFLLGLKKHGKGDWRNISRNFVTSRTPTQVASHAQKYFIRQL
SGGKDKRRASIHDITTVNLNDTRTPSPDNQSSLSPDQSTGLSQQPGSGGLSRTQFQWHQPNHGS
TTSFSSTQGSMLMTSPYGMNSYGLKMPGHNLQRSAVHEPFFGLQNLAFQMQYPHG

>MiMYB38 GWHPABLA023284

MRIMIKGGVWKNTEDEILKAAVMKYGKNQWARISSLLVRKSAKQCKARWYEWLDPSIKKTE
WTREEDEKLLHLAKLMPTQWRTIAPIVGRTPSQCLERYEKLLDAACAKDENYEPGDDPRKLRP
GEIDPNPESKPARPDPVDMDEDEKEMLSEARARLANTRGKKAKRKAREKQLEEARRLASLQK
RRELKAAGIDTRQRKRKRRGIDYNAEIPFEKKPPPGFFDVTDEDRPVEQVKFPTTIEELEGKRRI
DVEAQLRRQDIAKNKIAQRQDAPLAILQANKMNDPESVRKRSKLMLPAPQISDHELEEIAKMG
YASDLLAGNEDLSEGSGATRALLANYAQTPQQGMTPLRTPQRTPAGKGDAIMMEAENLARLR
ESQTPLLGGENPELHPSDFSGVTPKKREIQTPNPMLTPSATPGGVGLTPRIGMTPSRDGYSFGVT
PKGTPIRDELHINEEMDMHDSAKLEQRRQADLRRNLRSGLSNLPQPKNEYQIVIQPPAEDSEEP
EEKIEEDMSDRLARERAEEEARQQALLRKRSKVLQRELPRPPVASLELIRNSLLRADGDKSSFV
PPMSIEQADEMIRKELLTLLEHDNAKYPLEKVEKKKKGSKRSANGSAAPIPVIEDFEEEELKEA

DNLIKEETQYLRVAMGHENESLDEFVEAHNTCLNDLMYFPTRNAYGLSSVAGSMDKLAALQS
EFEVVKRKMDDDKEKALHLEKKVKLLTQGYEKRAENLRNQIQSTVKQMETAGTELECFQAL
QKQEQLAASNRINGLWEEVQKQKKLEQTLQKRYGDVLAELERLSQRIDEYRVQAQKQEEIAA
EKLALKAAEENHIIEQNSGASEALRSEKLGSSVPAEQSHDENPGPQIDAVHMDVDSGKDHITID
VDVRQNVVEANPDAVAPETVIHRGSSANEDVMEVPSAEVDNASLASKEAEANDNLSILNGDST
NKQTGEDVAISEPVNTELDGKQDNQENTVILADDD

>MiMYB39 GWHPABLA024160

MELETGTNLKQNHAPFFFSHENYIKSFIKSEILFDEGSSSKGYLQDFHHLSHHFNANGCSSNPM
FGVHIPCFDSLDVSAHAYSSLNFDFHEYKFQSGGYLENHHQIMNPLVDITGSNQSHMPLSFIEPV
NFVVSDEVSSVSADNISSYHKKAGMNKKNRPYLSTRTGKVGKKYNVVKGQWTIDEDRLLIRL
VEQHGMKKWSHIAQMLPGRIGSSVERDGITICGLISSWSEEEDKALIEAHAEIGNKWAEIAKRL
PGRTENSIKNHWNATKRRQFSKRKCRSKNQRASVLQDYIKTLNLTSTATKHRTKTSLKVNNKT
KILSNQAPVDNDEVSPCDHLVLDFDFSEVPDFDFDDDKLFEDDCSLDSLIEQMPCGSAVGDDD
KKSFEMELSPLDEKSQVGDMDLVEMICQANNM

>MiMYB40 GWHPABLA030438

MSHTTSDSDDRVLSRGQTDLPLVDEGGNSVQGVILKKGPWTSAEDAILIDYVKKHGEGNWNA
VQKNSGLFRCGKSCRLRWANHLRPNLKKGTFTQEEEQLIVELHAKMGNKWARMAAHLPGRT
DNEIKNYWNTRIKRRQRAGLPLYPPEVSFQALEESQNQNIGGINNGDKGHHDILQANGYEIPDV
VFDSLKGNQGVLPYVAELPDISCSNMLMKGLGSPFCSFMPPTIHREKRLRESVGLFSGYSERAK
NEFCSFDQFQNDTPDKMAPPFGLPFPLDQDPTTKAPETFGVIQGGQTLSNGNFSASTPTSGAVK
LELPSLQYPETDLSSWGTSSPPQPLLETVDSFIQSPPTGTVESDCPSPRNSGLLDALLHEAKTLSS
AKNHSSDKSSNSSSVTPDIADSCLPNVCQNEWEDYCDPLSPLGHSATSLFNECSPLSTSGSSLDE
QAPIEPYVGCKVKLEQVDQAWTPDRGKESSNLYDLSQADALLSSDWLEQGSAYVKNPTVITD
AIATFFGDDLGSEYKQMTTGTSTASQGWGFGSSAWNNMPAVCQMSELP

>MiMYB41 GWHPABLA030681

MPALISDKKEKMVSRFISSNGLVEDPCAVEKERAMINPWTVEEREIFMDKLATFGKDFRKIASF
LDYKTTADCVEFYYKNHKSDCFEKIKKRPVPVKPCSNTYFMRPGKKWDRQMNAASLDILGEA
TEMAAAFQADNVRQISGRISLGGCSDSKLSFGDDGMTERSSSFDVHGNERETAAADVLAGICG
SLSSEAMSSCITSSVDPGEGQQEWKHQKMDSFRRHSTSDVTQNVDDDTCSDESCGEMDPADW
TDEEKSIFIQAVSSYGKDFIMISRCVRTRSSDQCKVFFSKARKCLGLDLIHTGRGTAGTCVSDEA
NGGGSDTEDACVLETSSVISNDKMDSKVDDLPSSGVNKTRDKSDLGTMNLPTDLNKSGDENG
ERLLDDQDSKDVSPLVSDDCKRLHNPEVYSEFERMRMDNVDCQSESLQAHKIPVEFVEKEGG

KDKLAEQAVGLPVPLTSREAMKLCSSGLNVVIETTEAPVKGYENGLEDRIEPNKTCKADEHGG
RGMVQGSNVFGNIVDLTVDTNSCSVPSKSDSGDKLPVMSLPLENSLASVSSVPQVSAASQCEK
SVEQNRLSSTLDFQGNKDKSACMSVGNEDYHRNLLGHSLLNHVEHSQILKGYPLQISSKKEM
NGDISCRQLSEVQGISKSDRSDGVPYMAQDCYIQKCSSSMSHASVPELPFLASNLDQKNDPPRS
HSRSLSDTDRPRKNGDVKLFGKILSRPSSSQKPNSSSRENEEKGAHHHKQSCKVSNLKFTASHP
AEMLKFDRNNYLGLENVPMRSYGFWDGNRIQTGYSSLPDSAILLAKYPTAFGNYPGSSSKME
HQALQVKSSECNLKGVAVLPQREISSSNGVVDYQVYRSSDVTKVQPFAIDVKQRQDLLFSEMQ
RRNAFEALSTLPQPGRGMVGVNVVGRGGILVGGPCTGISDPVAAIKRHYAKADQYGGQSGSII
REEESWRSNGDIGRSVDSFSALFARFYSFI

>MiMYB42 GWHPABLA030846

METLYPALYMSDASWFLQESQSTGWTKEENKKFESALAIYSEGTPDRWIKVAALIPGKTVLDV
MKQYKVLEEDVNDIEAGKVPIPGYYSSSFALELVSERDFDANRKRSLVKSSDQERKKGVPWTE
EEHRLFLKGLLQYGKGDWRNISRHFVITKTPTQVASHAQKYFIRQLSGGKDKKRPSIHDITTVN
LADTNLPDNQKPCSVDQSNVLPQQQKSSSLPKVGLEWNDSNNGVVIFNPSNGNNLFVPSVNDI
GSNGLKLQGKNLYGTTYHGVHFKPRNSVF

>MiMYB43 GWHPABLA031111

MSQTTSDSDDRVLSRDQTDSPLMDEGGNSVQGVILKKGPWTSAEDAILIDYVKKHGEGNWNA
VQKNSGLFRCGKSCRLRWANHLRPNLKKGAFTQEEEQLIVELHAKMGNKWARMAAHLPGRT
DNEIKNYWNTRIKRRQRAGLPLYPPEVSFQALQESQNQNIGGINNGDKGHHDILQANGYEIPD
VVFDSLKANQSVLPYVPELPDISGSNMLMKGLGSPFCSFMPPTIHRQKRLRDSVELFSGYSGRV
KNEFPSFDQFQNDSPDKIAQTFGLSFPLDSDPTTKAPESFGVIQGSQTLSNGNFSASKPTSGAVK
LELPSLQYPETDLSSWGASSPPQPLLETVDSFIQSPPPTGTVESDCPSPRNSGLLDALLYEAKTLS
SAKNHSSDKSSNSSSVSPADIADSSTLNICETEWEDYGDRLSPLGHSATSFFNECTPLSTSGSSLD
EQAPVEPYVGCKVKSEPVDQTWTPDRGKVSTDLYDITQSDDLLSSDWLEQGSSYAKNPTVMN
DAIATLFGDDLGNEYKQMTTGTSTVSQGWGFGSCAWNNMPPVCQMSELP

>MiMYB44 GWHPABLA031168

MYQDPNAFHSSSSTHWNRLEDKLFEHALVLFPDEVPDRWQRIADQIPGKSPRDVKEHYEVLVH
DVTEIDSGRVELPCYADESDDWDSAGQISFGSKPVKQGEPERKKGTPWTEEEHKLFLIGLSKFG
KGDWRSISRNVVVSRTPTQVASHAQKYFLRQTNMKKERKRASIHDITTVDSKPIPLAVDQNSM
GLAVDQNSMGLPVDQNWNPPPAVSGHQQPPTYQQFPPPNQFLSQGGSMGYQNYGFPM

>MiMYB45 GWHPABLA032359

MKKSGGANGESKKKERHIVTWSQQEDDILREQISKHGTDNWSIIASKFKDKTTRQCRRRWYT
YLNSDFKKGGWSPEEDMLLCEAQKIFGNRWTEIAKVVSGRTDNAVKNRFSTLCKKRAKYEAL
AKENNNSYMNTNKKRILFQNAFNADGTPENTAPVKRVRRSHIPDLAQSCNIINRSHRQSGTTM
NPQLRPPFTVLVQNLPSVNSLPAQHQHRDIKEISNNAPQNNKTQVMFLKKDDPKITALMQQAE
LLSSLALKVNTENTEQSLENAWKVVQDYLNRSKENDILRYTISDIGFQFENFKDLIEDLRSSNEG
SHPSWRQPDLYEDSPASSEYSTGSTLIPHLASEKPDQIQAEVDILHQNIGAELQLMNNGEQHCL
EEQNKGHAKTNEVELFPSGGEITNNNGIVSVSSITEFSSPIQVTPLFRSLAAGIPSPKFSESERNFL
LRTLGIDSPSPNPSTNPVQPPPCKRALLQSL

>MiMYB46 GWHPABLA032392

MIYADIASLFLSSTHLRAGSVASASLEIYFCRNSDRENGRGSRNFPPIGNNSTRSFEGPHNIEVNV
DGEGSNYRDGFGENETVIHNVNLNEEFNHNETIEMSGKETDKGLAKLCARGHWRPAEDTKLK
ELVALYGPQNWNLIAEKLEGRSGKSCRLRWFNQLDPRINRSAFTEEEEERLMQAHRLYGNKWA
MIARLFPGRTDNAVKNHWHVIMARKYRKQSSAYRRRKLSQSVYKRMEHIQGIICRGTAARTEL
QPYCFSVPNGGFSNISPFSYGTYNGGGGGVNFSVNGSPQMTSGQQTTLSNKEPYSPKSKDIMG
MFSQVRSCDGPIDEGHVSGINLLNHRQYLMAMQQSSFQQHHPHSFFDAMASTPQVSASEASSS
VGSHFDTVPPRFFDFLGVGAT

>MiMYB47 GWHPABLA032959

MNRVLETDRIKGPWSPEEDQLLHQLVQCNGARNWSLISKSIPGRSGKSCRLRWCNQLSPEVQH
RPFTSDEDEIIMQAHATFGNKWATIARLLNGRTDNSVKNHWNSTLKRKYAMITTEDDNDDINE
RGRDSFYPEKKRSAPGSFSGNSSSPSGSDVSDSGLPAMSSSDMCGSVGTFVETENGSDKNINNN
GDVAFNVSTELTLNLPGNESNDLTQCWVPREVSQKEESSTDYNNKTRVVWPELMALMQEMIR
KEVRNYMEEAVTNNGIKGY

>MiMYB48 GWHPABLA033543

MTVDEVGSSSVWTKEQDKAFENALATYPEDASDRWEKIVSDIPGKTLDEIKHHYELLVDDVN
DIESGCVPVPSYNSSSDGSTGHGGDEGTGKKDSHYGHYNNESNHGSKSSRSDQERRKGIAWTE
DEHRLFLLGLDKYGKGDWRSISRNFVVTRTPTQVASHAQKYFIRLNSMNKDRRRSSIHDITSVS
NGDISAAQGPITGQTNGSGAGASSGKSAKQQPQHPAGPPAVGIYSTPTIGQPIGGPLVSAVGTPV
NLPAPVHMAYVVRAPVPGAVVPGAPMNVGAMPYPMPPTSAHRSPEMTSTPSSILFLTNKNTFI
HTKNHIHTSLNTSQQHTSNSPIWTATEIADSVNGKLLKRCPPGIICTDTRALQPNTNQWFFAITG
QHFDAHDFISPQLYGKGCVGVIGNRVCEGWDKGFIRIEGNGNINTVDSLINMASYARNKWFNG
VLVGVTGSVGKSTTKSMIAFVLESLGVNVFKSYANWNNRVGVALSLIRMFRNVDIAVLEMGM
SKKGEILELARMTRPDIRMVLNVGASHFGEFGEFRGGSNGQRLQNLLRNRKVLFGQRMGCDV
RLVAAETADEGLGVRVVLDKEKEIPGLHLALDACAAAVATLFGLSLAEVGNLFRNLFLCTRSEL
QVASNGIKIVNDAYNANPMSTRAAIDLLKSIACDGKRVAVLGDKLELGSIKTESHEEILRYCCDS

NIDLVGLAGNRFLKAAKNMNLNRVKKIVHANDAEVLAQKIVKRLKFNDVILMKGSCAMQME
KVVDAVNEMDIYIPPQEL

>MiMYB49 GWHPABLA015853

MVIHTSYPLISYSKEIVDGQPIFACSNCLPVKASKYEPAGHSFHAAALKLLGCEEDTNADDHKV
SNDKEQTSLPQYESYSSKGKKKSGTASNQQDHYALLGLSHLRYLATEDQIRKAYRETALKYHP
DKQAALLLAEESEAAKQAKKDEIESHFKAVQEAYEVLIDPVKRRIYDSTDEFDDEIPSDCSPQD
FFKVFGPAFMRNGRWSVNQPVPSLGDESTPLKEVDNFYNFWYSFKSWREFPHADEFDLEQAE
SRDHKRWMERQNSKLSERARKEEYVRVRALVDNAYKRDPRILKRKEEEKAEKQRKKEAKFL
AKKLQAEEAARAAEEEKRQKEEEEKRAAEAALQQKKLKEKEKKLLRKERTRLRTLSASVMES
KEGLEQAKVIRSALGIADKCEEKKQDEKNNLQQNGSVEANGSIPLKSFEKKEKPWEKEEIELL
RKGMLKYPKGTSRRWEVISEYIGTGRTVEEILKATKTVLLQKPDDSKAFNSFLEKRKPAQSITSP
LTTRENVEGVSTPQGAENTAAKVDIPEESSSSNSPMDVSAANGVSLISDQDVWSAVQERTLVQ
ALKTFPKETSQRWERVAAAVPGKTLNQCKKKFALLKENFRNRKTVA

>MiMYB50 GWHPABLA015906

MEAERERAIQVGGGEAAAESLCDGGGSGDDSVVVVGEGSRKGSKDRVKGAWSPQEDTILSNL
VSKFGARNWSLIARGISGRSGKSCRLRWCNQLDPAVKRKPFTDEEDQIIITAHAIHGNKWAVIAR
LLPGRTDNAIKNHCNSTLRRRALELGRVKKFESGSIMEDTNMDRTKASSEETLSCGDANSFKSL
EGKDVCSLEILDNQCENNTISEVPSNLEHELIEQPTLFRPQARVCAFNVYNTVDGPEIASLYPRQ
TPMQGPLGQASLSDAGICKMIEGGYSERTVPHLCGYGCCGIQCGGNCQNSLLGPEFLEFSEPPS
FPSYELAAIATDISNLAWMKSGLENSNMRMMDDAAGRIRTNGSIAKG

>MiMYB51 GWHPABLA016167

MKKRQRWKAEEDVLLRAYVKQYGPREWSLVSQRMNIPLNRDAKSCLERWKNYLKPGIKKGS
LTEEEQHLVIQLQAKYGNKWKKIAAEVPGRTAKRLGKWWEVFKEKQRREQKDNSTTVDPSEE
RKYDQILETFAEKLVKDGAFVMAASNGGFLHTESPTPSTLLPPWLSNSNGTSAIRPPSPSVTLSL
SPSTVATTPPIPWLPERGPDNAHLVLGNFLPHGSVSTCGENLLVSELVDCCRELEEGHRAWAAH
KKEAAWRLRRVELQLESEKACRRREKMEEIEAKVKALREEQRATLDKIEAEYREQIAGLRRDA
EAKEQKLAEQWAAKHLRLSKFLEQMGCRTRLPEPNNRCPLMFEILLYFHIYNNRNLYFLSGLN
LM

>MiMYB52 GWHPABLA016177

MAELKVEECCLENKQLTAASSSSVSEGSGSAILKSPVSSPATKSPTHRRTTGPIRRAKGGWTPEE
DETLRNAVATFKGKSWKKIAEFFPDRSEVQCLHRWQKVLNPDLVKGPWTQEEDDKIIELVSRY

GPTKWSVIAKSLPGRIGKQCRERWHNHLNPDIKKDAWTLEEELALMNAHRRNGNKWAEIAKV
LPGRTDNSIKNHWNSSLKKKLDFYLATGKLPPVAKNGPHNGSKDTNQSTAATKHFFIYSTRDSD
STAQTSSGTTDLGKLDEDGKDQLESSTPVLDMAASSCVRPNDSLDSEDVECKLESPNIDLSCSE
SMPKIENCAINSEHVDDRVIGSQQQIGTPTYGSIYYVPPQLKSSVPLDSDPLNTCSLQHECNSTPI
TSPISYFTPPCVKGSGLSARSPESILKIAAKTFPYTPSILRKRKSEGQVLSGTKITGKVDGETTDTS
SPVEPTGKDFNASPPYRLRSKRTAVFKSVERQLEFTFNKEKHDDNTKSRELSVNASCPVIEDLHI
TKLRVT

>MiMYB53 GWHPABLA019417

MRIMIKGGVWKNTEDEILKAAVMKYGKNQWARISSLLVRKSAKQCKARWYEWLDPSIKKTE
WTREEDEKLLHLAKLMPTQWRTIAPIVGRTPSQCLERYEKLLDAACAKDENYEPGDDPRKLRP
GEIDPNPESKPARPDPVDMDEDEKEMLSEARARLANTRGKKAKRKAREKQLEEARRLASLQK
RRELKAAGIDTRKRKRKRKGIDYNAEIPFEKKPPPGFFDVTDEDRPVEQVKFPTTIEELEGKRRI
DVEAQLRRQDIAKNKIAQRQDAPSAILQANKMNDPETVRKRSKLMLPAPQITDHELEEIAKMG
YASDLLSGNEELTEGSGATRALLANYAQTPQQVERITDTIIGRRDPELHPSDFSGVTPKKREIQTP
NPMLTPSATPGGVGLTPRIGMTPSTDGYSFGVTPKGTPIRDELHINEDMDIHDSAKLEQRRQAD
LRRNLRSGLSNLPQPKNEYQIVIQPPAEDNEEPEEKIEEDMSDRLARERAEEEARQQALLRKRS
KVLQRELPRPPVASLELIRNSLLGADGDKSSFVSPTSTEQADELIRKELLTLLEHDNAKYPFEKN
AEKKKKSSKRSANGSAVPIPVIEDFEEEELTEADKMIKEETQYLRVSMGHENESLDEFVEAHNT
CLNDLMYFPTRNAYGLSSVAVNVEKLAAFQSEFEIVKKKMDDDKEKALHLEKKVKLLTQGYE
KRAENLRNQIQSTVKQMETTGTELECFQALQKRELLGASNRINGLWEEVQKQKELEQTLQKR
YGDQLAELERVSQCINEYRIQAQKQEEIAAAKRALEAAAANKVIEQNSEASKSLPSGELGRSEP
VEPSHGENPGPQVGATHIDDDSGKYHIMPTTDVSENVVEANPDAAVLETVKDSGSADEHVIEV
PSAEGYNASLTSKESEAKENLPIMNGVSLDKQTGDDSAISELIDTELDGKTENVAIGD

>MiMYB54 GWHPABLA019508

MSLQHLVSDCHGFTVVHQDMNFVLPPPPSAPQLSLSNSFGVMRHHPMGEESNSPAAIMGLQIL
SPSSFKPEENRGAKRIVEESDGFLGIEKKGLSLNLGEEIEEVKTSVSVKTKHTKLCARGHWRPT
EDAKLKELVAQYGPQNWNLIAENLEGRSGKSCRLRWFNQLDPRINRRAFNEEEEERLLAAHRL
YGNKWALIARLFPGRTDNAVKNHWHVIMARKLREQSSIYRRRKPTSASQIGLENMIIPKNNAG
CDSTTISSNESASTITDLSLTPSSAKAPPAIFTRFSPMGKCSSADKGITRGSTGDVDKFHGYFKAG
KMEVAMAVDQSGNSSDSNSEVSATDSVTNINPITNLSLSGENENAYQKKTHMHFIDFLGVGAS
